# CFTR interactome mapping using the mammalian membrane two‐hybrid high‐throughput screening system

**DOI:** 10.15252/msb.202110629

**Published:** 2022-02-14

**Authors:** Sang Hyun Lim, Jamie Snider, Liron Birimberg‐Schwartz, Wan Ip, Joana C Serralha, Hugo M Botelho, Miquéias Lopes‐Pacheco, Madalena C Pinto, Mohamed Taha Moutaoufik, Mara Zilocchi, Onofrio Laselva, Mohsen Esmaeili, Max Kotlyar, Anna Lyakisheva, Priscilla Tang, Lucía López Vázquez, Indira Akula, Farzaneh Aboualizadeh, Victoria Wong, Ingrid Grozavu, Teuta Opacak‐Bernardi, Zhong Yao, Meg Mendoza, Mohan Babu, Igor Jurisica, Tanja Gonska, Christine E Bear, Margarida D Amaral, Igor Stagljar

**Affiliations:** ^1^ Donnelly Centre University of Toronto Toronto ON Canada; ^2^ Department of Biochemistry University of Toronto Toronto ON Canada; ^3^ Programme in Translational Medicine The Hospital for Sick Children Toronto ON Canada; ^4^ Division of Gastroenterology, Hepatology and Nutrition Department of Pediatrics University of Toronto Toronto ON Canada; ^5^ Faculty of Sciences BioISI‐Biosystems and Integrative Sciences Institute University of Lisboa Lisboa Portugal; ^6^ Faculty of Life Sciences and Medicine School of Bioscience Education King’s College London London UK; ^7^ Department of Biochemistry, Research and Innovation Centre University of Regina Regina SK Canada; ^8^ Department of Physiology University of Toronto Toronto ON Canada; ^9^ Program in Genetics and Genome Biology The Hospital for Sick Children Toronto ON Canada; ^10^ Osteoarthritis Research Program Division of Orthopedic Surgery Schroeder Arthritis Institute University Health Network Toronto ON Canada; ^11^ Data Science Discovery Centre for Chronic Diseases Krembil Research Institute University Health Network Toronto ON Canada; ^12^ Faculty of Medicine Josip Juraj Strossmayer University of Osijek Osijek Croatia; ^13^ Department of Molecular Genetics University of Toronto Toronto ON Canada; ^14^ Departments of Medical Biophysics and Computer Science University of Toronto Toronto ON Canada; ^15^ Institute of Neuroimmunology Slovak Academy of Sciences Bratislava Slovakia; ^16^ Mediterranean Institute for Life Sciences Split Croatia; ^17^ School of Medicine University of Split Split Croatia

**Keywords:** cystic fibrosis, high‐throughput screening, integrative computational biology, interactome, mammalian membrane two‐hybrid, Membranes & Trafficking, Methods & Resources

## Abstract

Cystic Fibrosis Transmembrane Conductance Regulator (CFTR) is a chloride and bicarbonate channel in secretory epithelia with a critical role in maintaining fluid homeostasis. Mutations in CFTR are associated with Cystic Fibrosis (CF), the most common lethal autosomal recessive disorder in Caucasians. While remarkable treatment advances have been made recently in the form of modulator drugs directly rescuing CFTR dysfunction, there is still considerable scope for improvement of therapeutic effectiveness. Here, we report the application of a high‐throughput screening variant of the Mammalian Membrane Two‐Hybrid (MaMTH‐HTS) to map the protein–protein interactions of wild‐type (wt) and mutant CFTR (F508del), in an effort to better understand CF cellular effects and identify new drug targets for patient‐specific treatments. Combined with functional validation in multiple disease models, we have uncovered candidate proteins with potential roles in CFTR function/CF pathophysiology, including Fibrinogen Like 2 (FGL2), which we demonstrate in patient‐derived intestinal organoids has a significant effect on CFTR functional expression.

## Graphical abstract

A new MaMTH‐HTS platform is used with a Human ORFeome library to map the protein‐protein interactions of full‐length wildtype and F508del CFTR. Functional validations in multiple disease models uncovered proteins with potential roles in CFTR function and cystic fibrosis.

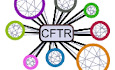

## Introduction

Thought to have originated in Europe (Morral *et al*, 1994) and spread to other regions with heterozygote advantage (Knudson *et al*, 1967; Gabriel *et al*, 1994; Schroeder *et al*, 1995; Molinski, 2016), Cystic Fibrosis (CF) is the most common autosomal recessive disease in the Caucasian population, occurring in approximately 1/3,500 births (Mehta *et al*, 2010) and affecting over 90,000 individuals worldwide. CF affects multiple organs with epithelial tissues including the lungs, pancreas, liver, intestines and the reproductive tract, resulting in significant life‐shortening and physical challenges. The most prevalent symptoms develop in the airways of CF patients with accretion of viscous mucus causing obstruction and an increased susceptibility to bacterial infection and neutrophilic inflammation. Development of CF‐related diabetes, pancreatic insufficiency, intestinal obstruction and liver disease are also present in individuals affected by CF, albeit with considerable phenotypic variation. Despite the recent approvals of drug therapies that pave the way for personalized medicine treatments of CF with possible disease‐modifying effects (Fajac & Wainwright, 2017; Amaral *et al*, 2019; Lopes‐Pacheco, 2020), pulmonary function of CF patients progressively decreases due to mucus obstruction/bronchiectasis, recurrent bacterial infection and subsequent inflammation causing high morbidity and mortality (Cutting, 2014). In addition, variation in survival and disease severity is extremely large and this heterogeneity is mostly attributed to differences in environmental factors and patients’ genetic background (i.e. modifier genes).

Pathophysiology of CF is caused by mutations in the Cystic Fibrosis Transmembrane Conductance Regulator (*CFTR*) (Riordan *et al*, 1989) gene, whose protein product is localized primarily in the apical membrane of secretory epithelial cells. The CFTR protein, regulated by PKA‐phosphorylation and nucleotide binding, functions as an anion channel that mediates the flux of chloride (Cl^−^) and bicarbonate (HCO_3_^−^) ions (Riordan, 2008), which provide the driving force for fluid transport and maintenance of ion and fluid homeostasis. As its name suggests, CFTR also acts as a regulator influencing the activity of a variety of other channels and transporters and consequently, fluxes of other ions (Li & Naren, 2010). The protein is a member of the human ATP‐Binding Cassette (ABC) transporter superfamily and thus has a conserved core structure consisting of two membrane‐spanning domains (MSD1 & MSD2) forming the pore of the channel, and two cytosolic nucleotide binding domains (NBD1 & NBD2) for ATP binding/hydrolysis. However, it also contains a unique regulatory domain (RD) that controls the channel’s activity based on its phosphorylation state. Notably, CFTR’s involvement in promoting the transmembrane flow of anions down their electrochemical gradient (thereby regulating electrolyte and water homeostasis across epithelial cell membranes) is distinct from the activity of other ABC membrane transporters, which direct the movement of organic substrates across cell membranes (Riordan *et al*, 1989; Bear *et al*, 1992; Molinski, 2016).

Being a twelve‐pass transmembrane protein, CFTR requires a multitude of accessory proteins and cooperative interdomain assembly to progress through a sequence of translational folding events (Riordan, 2005; Wang *et al*, 2006; Du & Lukacs, 2009; Pitonzo *et al*, 2009). As a result, successful folding and processing of the 1,480 amino acid‐long CFTR polypeptidic chain into a functional channel is a naturally inefficient process and a significant amount of wild‐type (wt) CFTR is rapidly degraded before reaching the plasma membrane (PM) (Ward & Kopito, 1994). This intrinsic property of CFTR biogenesis leads to the unsurprising observation that mutations which affect this process, or the quantity/quality of CFTR protein, are responsible for the majority of CF cases. Such mutations are ultimately associated with the loss of CFTR function, most prominently at the surface of the airways, which leads to depletion of the airway surface fluid and accumulation of dehydrated and persistent mucus, the hallmark feature of CF (Boucher, 2007).

There are currently over 2,100 variants in the *CFTR* gene recorded in the CF Mutation Database (http://www.genet.sickkids.on.ca/cftr/StatisticsPage.html), however F508del is by far the predominant mutation, present in ~80% of all individuals with CF worldwide (Cystic Fibrosis Foundation, 2020; Zolin *et al*, 2020). This mutation causes CFTR misfolding and subsequent retention in the endoplasmic reticulum (ER) from where it is targeted for degradation (Cheng *et al*, 1990). Interestingly, proper folding and function of this mutant protein can be partially restored by low temperature (Denning *et al*, 1992), which revealed that post‐translational processes play important roles in the manifestation of CF (Hutt *et al*, 2010). However, even upon rescue, F508del‐CFTR displays altered channel activity and reduced protein stability at the cell surface (Okiyoneda *et al*, 2010; Lukacs & Verkman, 2012), suggesting that there are many interacting proteins involved in its cell surface tethering and functionality (Riordan, 2005). Although the genetic cause of CF by CFTR mutations has been well studied in the past (Zielenski & Tsui, 1995), considerably less is known about the cellular protein environment of CFTR protein in CF.

In an effort to better understand CFTR protein regulation and biology, as well as the cellular changes associated with the F508del mutation and CF, we set out to map the shared and differential protein interactions of wt‐ and the F508del‐CFTR mutant in mammalian cells. To accomplish this, we applied a newly modified, high‐throughput screening (HTS) variant of our previously reported Mammalian Membrane Two‐Hybrid (MaMTH) technology (Petschnigg *et al*, 2014, 2017; Saraon *et al*, 2017, 2020, 2021; Yao *et al*, 2017; Aboualizadeh *et al*, 2021) called MaMTH‐HTS, to screen the Human Open Reading Frame (ORF)eome V8.1 collection (Yang *et al*, 2011). Subsequent computational and functional analysis of our interactomes identified several interactors with potentially significant roles in CFTR regulation and trafficking, furthering our understanding of CFTR/CF biology and identifying potential new avenues for future exploration.

## Results

### Design of the mammalian membrane two‐hybrid high‐throughput screening (MaMTH‐HTS) platform

The MaMTH technology is a split ubiquitin‐based assay for measurement of the protein–protein interactions (PPIs) of full‐length integral membrane proteins directly in the context of living mammalian cells (Johnsson & Varshavsky, 1994; Stagljar *et al*, 1998; Scheper *et al*, 2003; Paumi *et al*, 2008; Petschnigg *et al*, 2014, 2017; Sokolina *et al*, 2017; Yao *et al*, 2017) (Appendix Fig S1). It is highly sensitive and capable of detecting subtle alterations in PPIs in response to environmental factors and mutation state, making it an ideal tool for monitoring dynamic changes between the interactomes of wt‐ and F508del‐CFTR proteins in their native plasma membrane environment (Petschnigg *et al*, 2014, 2017; Yao *et al*, 2017).

In its original format, MaMTH functions as an “array‐based” assay, whereby a target “bait” protein of interest is tested against candidate interacting “prey” protein partners individually in the wells of microtitre plates (Petschnigg *et al*, 2014, 2017; Aboualizadeh *et al*, 2021; Grozavu *et al*, 2022) While suitable for medium throughput screens of small cDNA libraries (i.e. hundreds) of candidate interactors, the approach becomes less efficient as library size increases. In order to perform our CFTR MaMTH screens against an ORF library representing a significant portion of human genome (generated from the Human ORFeome V8.1, a large and relatively comprehensive collection consisting of ~12,000 sequence verified ORFs), we therefore needed to modify MaMTH to increase its throughput.

**Figure 1:**
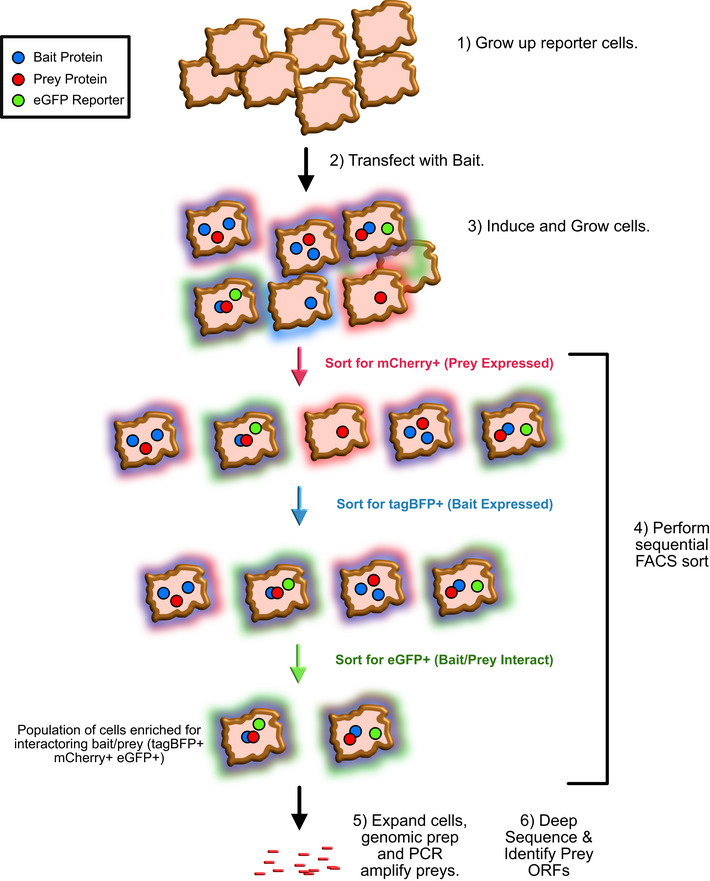
The MaMTH‐HTS workflow 1) Grow up cell lines containing stably integrated prey and reporter. 2) Transfect cells with Bait of Interest. 3) Induce the cells with tetracycline to express Bait and Prey. Continue to grow the cells for 48 h. At this point, we will have a mixed population containing the cells expressing combinations of Bait (along with tagBFP/blue fluorescence) and Prey (along with mCherry/red fluorescence). The cells expressing interacting Bait/Prey pairs will induce eGFP reporter and have additional green fluorescence. 4) Carry out sequential FACS sort, first selecting the cells with red fluorescence (indicating proper Prey expression), then blue fluorescence (indicating proper Bait expression) and finally green fluorescence (indicating Bait/Prey interaction). 5) Expand/grow the cells to increase overall cell number, isolate genomic DNA and PCR amplify using prey cassette specific primers. 6) Deep sequence amplified PCR products to identify prey ORFs enriched in population (indicating potential interactors).

To accomplish this, we assembled a new MaMTH‐HTS workflow. In this approach, we employ fluorescence, instead of luminescence, as a readout for PPIs, using an eGFP reporter in place of luciferase (Petschnigg *et al*, 2014) (Appendix Fig S1). This allows for easy enrichment of cells expressing interacting protein pairs using Fluorescence‐Activated Cell Sorting (FACS). To help minimize background, tagBFP (a monomeric blue fluorescent protein) and mCherry (a monomeric red fluorescent protein) tags are also attached to MaMTH baits and preys, respectively, via a P2A linker. In this way, the cells can first be FACS‐sorted based on the proper expression of “prey” protein of interest (indicated by red fluorescence) and “bait” (indicated by blue fluorescence) prior to selection of the cells containing interacting bait‐prey pairs (indicated by green fluorescence). Sorted populations are then expanded, followed by genomic DNA isolation, PCR amplification of prey ORFs and deep sequencing, to determine which “prey” ORFs are enriched in the final population, providing a list of candidate protein interactors. While still new and awaiting substantial benchmarking, in its current format our MaMTH‐HTS platform presents a relatively fast and simple means of screening large numbers of interactions involving full‐length membrane proteins directly in the environment of living mammalian cells. This potentially allows for detection of interactions that may be missed using systems with more artificial screening conditions or that require the use of only the soluble regions of membrane proteins. A schematic overview of the MaMTH‐HTS platform can be found in Fig 1.

### MaMTH‐HTS Screening of wt‐ and F508del‐CFTR

In order to map wt‐ and F508del‐CFTR interactomes using the MaMTH‐HTS system, we first generated CFTR “bait” constructs and an appropriately tagged “prey” library for high‐throughput PPI screening. “Baits” were prepared by cloning wt‐ and F508del‐CFTR ORFs into MaMTH plasmid expressing (under the control of a tetracycline‐inducible promoter) the full‐length proteins as fusions to a C‐terminal bait tag consisting of the C‐terminus of ubiquitin (Cub) and a GAL4 transcription factor linked by a P2A sequence to tagBFP (Appendix Fig S2A).

For ORF library generation, we cloned the entire Human ORFeome V8.1 (Yang *et al*, 2011), consisting of ~12,000 ORFs divided into 33 distinct pools, into a CRISPR‐compatible MaMTH‐HTS “prey” vector. This vector allowed for tetracycline‐controlled expression of proteins fused at their N‐terminus to the N‐terminal fragment of ubiquitin (Nub) and at their C‐terminus to a P2A‐mCherry tag (Appendix Fig S2B). Following cloning, ORF representation in pools was verified by deep sequencing and individual pools were then stably integrated (using a variant of the *S. pyogenes* CRISPR/Cas9 system (Ran *et al*, 2013) developed in our lab) into the AAVS site of MaMTH HEK293 reporter cells stably expressing eGFP under the control of a 5XGAL4‐UAS. To determine representation of ORFs in these cell lines, integrated preys were amplified by PCR using MaMTH prey‐cassette specific primers and deep sequenced, verifying the presence of 10,228 ORFs in the final cell library population (Dataset EV1).

For screens, all 33 ORF “prey” cell pools were combined into a single “master pool” and transfected with either wt‐ or F508del‐CFTR MaMTH bait plasmid. Note that we previously demonstrated that transient transfection of MaMTH CFTR baits does not affect their expected localization (Appendix Fig S3), making this approach ideal for streamlining the screening process. The cells were then grown for 2 days in the presence of 0.5 µg/ml of tetracycline to allow for significant expression of baits, preys and associated fluorophores/fluorophore reporter. Following growth, the cells were harvested and sorted by flow cytometry using a BD FACS Melody (BD Biosciences). Sorting was performed sequentially based on the presence of mCherry fluorescence (indicating cellular expression of prey), tagBFP fluorescence (indicating cellular expression of bait) and finally eGFP fluorescence (indicating a bait/prey interaction). The collected cells were resuspended in appropriate media and grown/expanded for 10 days to increase overall cell number. Genomic DNA was then harvested from the cells and prey ORFs amplified using two distinct sets of MaMTH cassette‐specific primers (Table EV1). Purified PCR products were then prepared and deep sequenced separately, providing us with two technical replicates and resulting data analysed using in house bioinformatics software to assemble a preliminary list of putative interactors (Dataset EV2).

### Identification of the wt‐ and F508del‐CFTR Interactomes

Our initial mapping identified a total of 494 unique candidate PPIs across both wt‐ and F508del‐CFTR, from which we were able to flag and filter out 47 as “frequent fliers” based on repeated occurrence across MaMTH‐HTS screens of 9 control “baits”, belonging to distinct classes of membrane proteins, as determined using the rank product method (Breitling *et al*, 2004) (Datasets EV2 and EV3).

**Figure 2:**
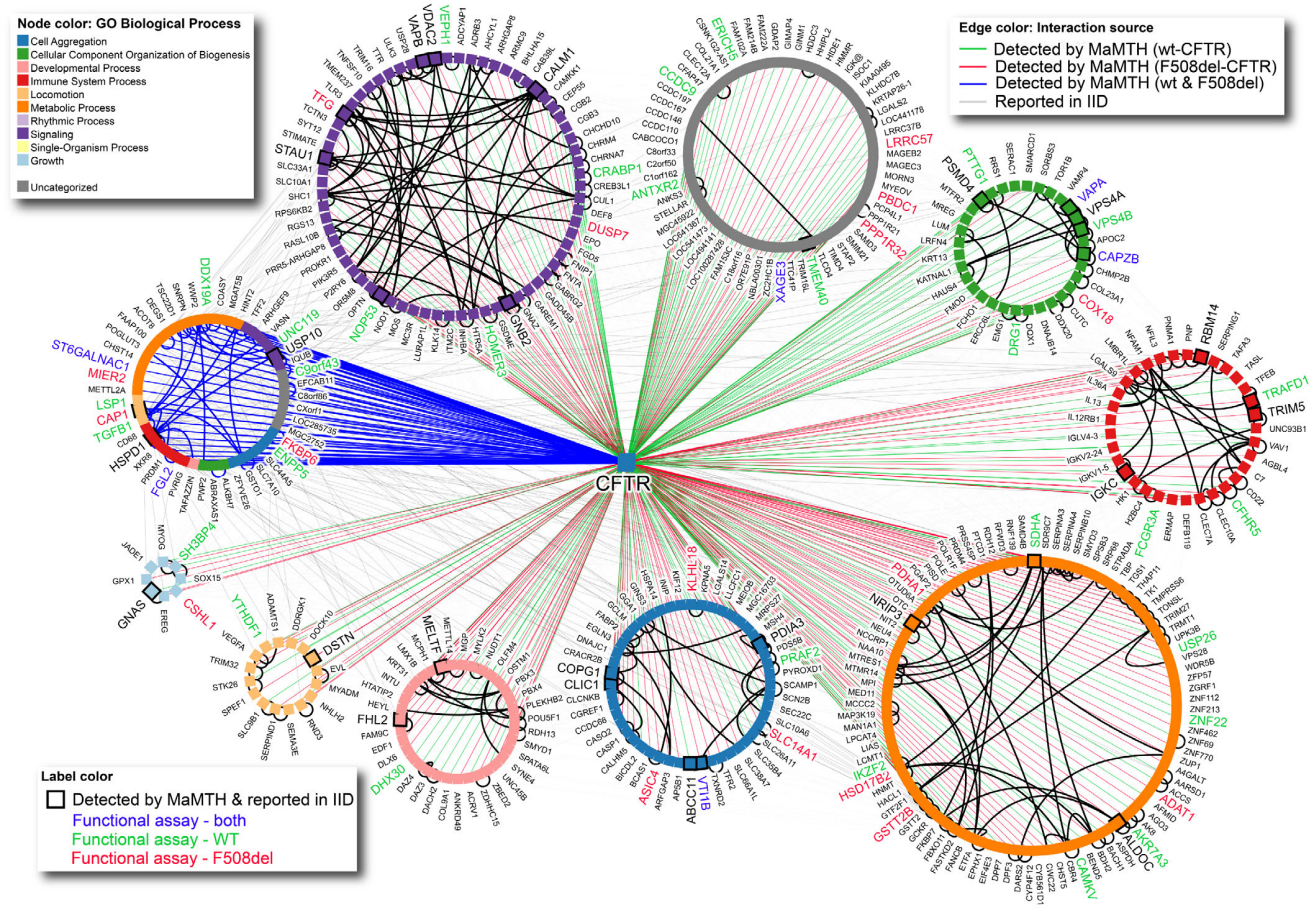
MaMTH‐HTS interactome for wt‐ and F508del‐CFTR Annotated MaMTH‐HTS interactome for wt‐ and F508del‐CFTR (green, red and blue edges) expanded with known experimental and predicted interactions from IID v 2021‐05 (black/grey edges). Interactions observed in MaMTH‐HTS to be shared by both wt and F508del CFTR are indicated by blue edges, while those observed with only wt or F508del are indicated by green and red edges, respectively. Node colour represents Gene Ontology Biological process. Black square outline and larger node label indicate CFTR interactions overlapping with IID. Node label colouring highlights interactors functionally validated in this work.

Of the remaining 447 candidate interactions, a total of 224 interacted with wt‐CFTR, with a robust overlap of 209 (~93%) between both technical replicates (Appendix Fig S4A, Dataset EV2). Similarly, a total of 269 unique ORFs interacted with F508del‐CFTR, with an overlap of 250 (~93%) between technical replicates (Appendix Fig S4B, Dataset EV2). Collectively, these overlaps demonstrate that our data do not appear to be greatly influenced by technical issues such as primer‐specific PCR bias or inconsistencies across sequencing runs. Comparison of the wt‐ and F508del‐CFTR interactomes revealed an overlap of 46 interactors (~10.2%), with wt‐ and F508del‐CFTR having 178 and 223 unique interactions, respectively (Appendix Fig S4C, Dataset EV2). A complete, annotated version of our interactome, sorted by GO Biological process and showing shared/differential interactions and interaction novelty based on functional and predicted PPIs reported in the IID database (Kotlyar *et al*, 2019) is provided in Fig 2 (with additional annotation details provided in Dataset EV4). Notably, our interactors cover a broad range of functions, with some of the largest groups corresponding to signalling, locomotion and, intriguingly, uncharacterized processes. A more focused sub‐interactome highlighting previously reported interactions is also provided in Appendix Fig S5.

### Evaluation of CFTR Interactomes using traditional MaMTH

While MaMTH‐HTS is based on the same general principles as our originally published array‐based MaMTH assay (Petschnigg *et al*, 2014, 2017; Aboualizadeh *et al*, 2021), the important changes introduced into the format (including the use of a multi‐step, fluorescence‐based selection/reporter system, stably versus transiently expressed preys, pooled screening that involves testing thousands of potential interactions simultaneously in a mixed population and a deep sequencing readout) suggest that the systems may behave differently in terms of interaction mapping. To further explore this, we re‐tested a large subset of 240 identified interactions using the original MaMTH assay with transiently expressed bait‐prey pairs in HEK293 cells containing a *Gaussia princeps* luciferase reporter system.

Each interaction was tested in three independent trials with three technical replicates. From a total of 138 wt‐CFTR interactions, 32 (~23%) produced a statistically significant luminescence signal (with respect to background) in our original MaMTH (Dataset EV5 and Appendix Fig S6A). The proportion of F508del‐CFTR interactions was slightly lower, with 26 out of 154 tested (~17%) testing positive (Dataset EV5 and Appendix Fig S6B). These results confirm that while both MaMTH versions can robustly detect certain interactions, MaMTH‐HTS was able to detect a considerable number of PPIs not identified by the originally described MaMTH system (Petschnigg *et al*, 2014).

Similarly, our traditional MaMTH format was able to detect interactions missed using our HTS format. Specifically, array‐based counter‐screening of 86 interactors, identified in MaMTH‐HTS as uniquely interacting with wt‐CFTR, against F508del‐CFTR bait found that 18 (~21%) also interacted with the F508del mutant (Dataset EV5 and Appendix Fig S6C). Conversely, testing of 102 interactors, identified in MaMTH‐HTS as uniquely interacting with F508del‐CFTR, against wt‐CFTR bait revealed that 23 (~23%) also interacted with wt‐CFTR (Dataset EV5 and Appendix Fig S6D).

Collectively, while some of these discrepancies are likely attributable to variation/technical issues during screening, our results still suggest that the platforms have highly complementary behaviours suitable for detection of distinct types of interactions.

### Fluorescence‐based assay to monitor CFTR channel activity

To explore the biological relevance of the identified PPIs, we performed a previously reported membrane potential assay (Ahmadi *et al*, 2017) using FLIPR^®^ dye to monitor the ability of a subset of 26 interactors, selected based on performance in MaMTH‐HTS and traditional MaMTH, to modulate the activity of CFTR baits (Dataset EV6).

**Figure 3:**
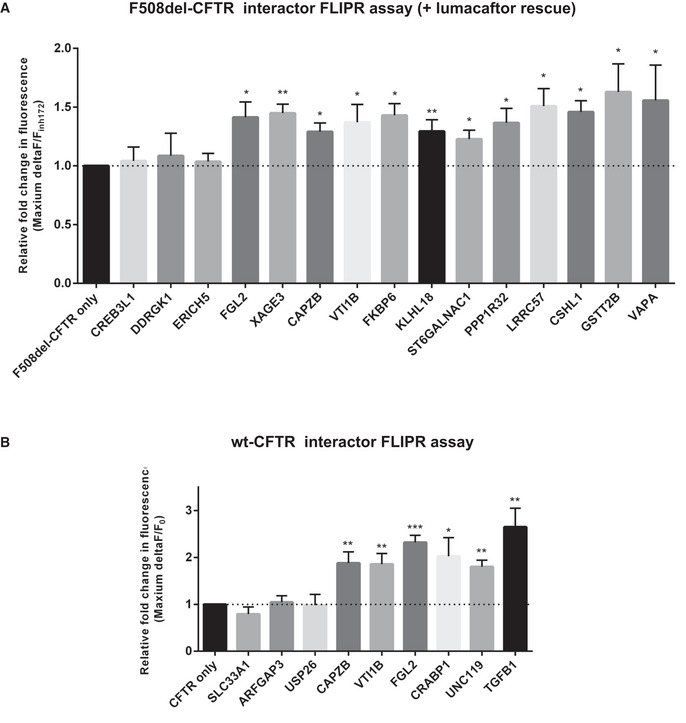
FLIPR assay results for interactors positively affecting CFTR Cl^‐^ channel activity A, BAssays were performed in HEK293 cells for both A) F508del‐CFTR and B) wt‐CFTR. For the wild‐type measurements, we compared the maximum Fsk response (Maximum *F/*F_0_; where *F*_0_ represents baseline RFU right before the stimulation). For the F508del‐CFTR measurements, since this mutant does not respond well to Fsk alone (Fsk + VX‐770 potentiator is required to obtain a detectable signal), we compared the CFTR inh172 (inhibitor) response (F_1_/“F‐_CFTRinh172_”; where F_1_ represents peak RFU immediately before inhibitor addition and “F‐_CFTRinh172_” is the minimum RFU reached at the end of the CFTR inh172 measurement cycle). For each of wt‐CFTR and F508del‐CFTR, data for three proteins not detected as interactors (first three bars following CFTR only) are shown as negative controls. Fluorescence signals were normalized to the CFTR‐only control and the statistical significance of differences assed by unpaired *t*‐test (*P < 0.05, **P < 0.01 and ***P < 0.001). Samples run in *n* = 3 replicates, and all values are mean ± SD.

For these experiments, each interactor was transiently expressed in HEK 293 cells stably expressing either wt‐ or F508del‐CFTR bait. Activation of CFTR channels in these cells can be detected as membrane depolarization using the FLIPR^®^ membrane potential‐sensitive dye after stimulation by forskolin (Fsk) and ivacaftor (VX‐770), while CFTR Inh_172_ inhibitor can impede this fluorescence response (Ahmadi *et al*, 2017). For the wt‐CFTR measurements, we compared the maximum Fsk response before and after stimulation. For F508del‐CFTR, since it does not produce a detectable signal upon addition of Fsk alone (Fsk plus a potentiator drug such as VX‐770 or genistein are required), we compared the fully stimulated response immediately before the addition of CFTR Inh_172_ inhibitor to the minimum response reached at the end of each measurement cycle. Using this approach, we detected a total of 15 interactors (of 26 tested in total) whose transient expression appeared to significantly affect wt‐ and/or F508del‐CFTR functional channel activity in response to Fsk and VX‐770 potentiator stimulation compared to that of the dimethyl sulfoxide (DMSO) control. Specifically, 12 interactors increased F508del‐CFTR channel activity (Fig 3A) and 6 interactors increased wt‐CFTR activity (Fig 3B). Of these, 3 interactors (FGL2‐Fibrinogen Like 2, VTI1B‐Vesicle transport through interaction with t‐SNAREs homolog 1B and CAPZB‐F‐actin‐capping protein subunit beta) were “shared” between the wt‐CFTR and F508del‐CFTR, leading to an increase in the channel activity of both forms of CFTR (Fig 3A and B). Assay traces for the experiments with these interactors can be found in Appendix Fig S7.

### Traffic‐based siRNA high‐content microscopy screen of top MaMTH‐HTS interactors

To determine whether interaction partners identified via MaMTH‐HTS regulate CFTR (wt‐ and F508del‐) plasma membrane expression, we performed a high‐content siRNA‐based microscopy assay (Botelho *et al*, 2015) to measure CFTR trafficking upon knocking down the genes encoding for selected interactors. Specifically, a total of 208 genes expressing interaction partners identified with MaMTH‐HTS were targeted (using 414 siRNAs, 2 for most gene targets) and the effects of their knock‐down on an mCherry‐Flag‐CFTR traffic reporter expressed in CF bronchial epithelial (CFBE) cells were assessed (Dataset EV7).

**Figure 4:**
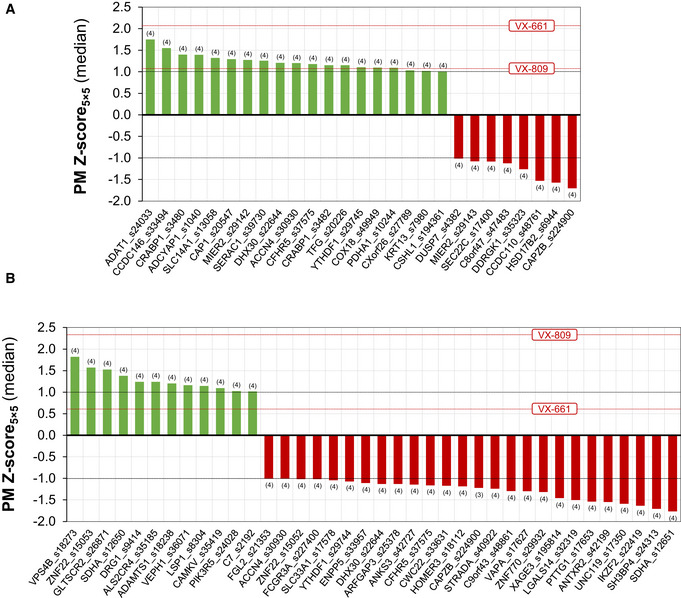
High‐content trafficking assay coupled with systematic siRNA library screening A, BInteractors identified in MaMTH‐HTS that, when knocked‐down in CFBE cells, affect CFTR membrane trafficking. Interactors affecting A) F508del‐CFTR and B) wt CFTR are indicated. Green bars (all with a *Z*‐score greater than or equal to 1) indicate a significant enhancement of CFTR trafficking to the PM. Red bars (all with a *Z*‐score of less than or equal to −1) indicate a significant reduction in CFTR PM trafficking. The “rescued” trafficking effects of the corrector compounds lumacaftor (VX‐809) and tezacaftor (VX‐661) are shown, as dotted red lines, for comparison. All conditions were measured across *n* = 4 biological replicates and the number of valid replicates is indicated between parenthesis for each condition.

By taking the cells treated with a non‐targeting siRNA (siNeg1) as a reference and considering the siRNAs generating a significant increase or decrease in PM localization as those with *Z*‐score > +1 or *Z*‐score < −1, respectively, (a strategy successfully followed in several previous related screening assays (Botelho *et al*, 2015; Lérias *et al*, 2018; Santos *et al*, 2020)), we identified 25 genes whose knockdown by at least one siRNA leads to modulation of F508del‐CFTR PM expression (Fig 4A, Dataset EV7). Of these, 17 rescued F508del‐CFTR PM expression, while 7 worsened its PM trafficking (Fig 4A, Dataset EV7). One gene, MIER2 (Mesoderm induction early response protein 2), produced an ambiguous result, with an increase or decrease in F508del‐CFTR PM level observed depending on the siRNA used (Fig 4A, Dataset EV7).

Using the same thresholds, we also identified 36 genes whose siRNA‐mediated knockdown by at least one siRNA affected wt‐CFTR PM expression. These included 10 genes resulting in an increase in wt‐CFTR PM expression and 24 that lead to a decrease (Fig 4B, Dataset EV7). There were also two interactors, SDHA1 (Succinate dehydrogenase [ubiquinone] flavoprotein subunit, mitochondrial) and ZNF22 (Zinc finger protein 22), which produced an ambiguous result similar to that observed with MIER2 siRNA for F508del‐CFTR, leading to an increase or decrease in wt‐CFTR PM trafficking depending on the siRNA used (Fig 4B, Dataset EV7).

Interestingly, comparison of results revealed that the siRNA targeting one particular gene, CAPZB, led to a significant decrease in trafficking of both wt‐ and F508del‐CFTR (Fig 4A and B and Dataset EV7), highlighting a potentially important role in general localization of CFTR channels to the PM. Disruption of some candidate genes also lead to opposing effects on the CFTR variants. For instance, siRNAs targeting DHX30 (ATP‐dependent RNA helicase DHX30), ACCN4 (Acid‐sensing ion channel 4), CFHR5 (Complement factor H‐related protein 5), YTHDF1 (YTH domain‐containing family protein 1) and CSHL1 (Chorionic somatomammotropin hormone‐like 1) all lead to a decrease in wt‐CFTR trafficking, while that of F508del‐CFTR was increased (Fig 4A and B and Dataset EV7). These results suggest a specific role of these genes in intracellular retention of F508del‐CFTR, which is alleviated with the knockdown.

Representative images of wt‐CFTR localization and F508del‐CFTR trafficking rescued in response to siRNA hits (alongside associated controls) are provided in Appendix Fig S8.

### Validation of traffic‐based siRNA high‐content microscopy screens

**Figure 5:**
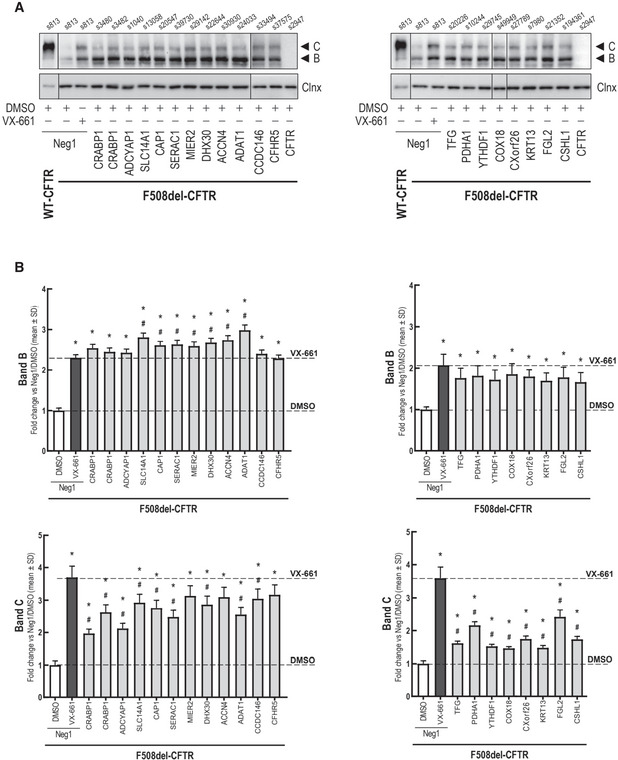
Western blot analysis of siRNA‐mediated knockdown of selected MaMTH‐HTS interactors on CFTR glycosylation in CFBE cells Blot images showing bands B and C as well as the calnexin internal control.Blot quantification showing relative fold change of band B and band C versus treatment with a non‐targeting siRNA (Neg1). One‐way Anova followed by Dunnett's post hoc test was performed to determine significance versus baseline (siNeg1 + DMSO treatment, *P < 0.05) and 5 µM VX‐661 (^#^ *P* < 0.05). Measurements were obtained from biological triplicates (*n* = 3) and all values are mean ± SD.

To validate the candidates affecting CFTR trafficking identified from our high content microscopy screen, we performed siRNA knockdowns of these genes and used Western blot (WB) to assess the abundance of post‐ER CFTR, that is fully glycosylated forms (Fig 5A). These experiments included siRNAs targeting the 18 candidate genes whose knockdown rescued F508del‐CFTR PM trafficking as well as FGL2, as overexpression of this protein had a significant effect on F508del‐CFTR channel activity in our FLIPR assays. While FGL2 knock‐down did not meet the traffic *Z*‐score threshold (>+1), it did produce consistently positive Z‐score values of 0.46 and 0.75 across both siRNAs.

Notably, we observed that down‐regulation of each of the 19 candidates resulted in significantly increased amounts of the fully glycosylated F508del‐CFTR form (“band C”) versus baseline, in some instances reaching levels comparable to those obtained with the pharmacological corrector VX‐661 (Fig 5A and B). Interestingly, all knockdowns also stabilized the core‐glycosylated “band B” form—characteristic of the ER—to levels comparable to VX‐661. These observations confirm the hypothesis that these genes may be involved in retaining F508del‐CFTR in the ER or reducing CFTR biogenesis efficacy. Knockdown of FGL2 also led to an increase in the amount of the fully glycosylated form of CFTR, indicating an improvement in CFTR processing. This result is particularly intriguing, as overexpression of the protein was observed to lead to an increase of CFTR channel activity in our FLIPR assay (Fig 3), suggesting a potentially complex or cell line‐dependent role of the protein in CFTR regulation.

### Orthogonal validation using traditional co‐immunoprecipitation (co‐IP)

To further validate the quality of our interactome, we performed tag‐based co‐IP assays on a selected subset of 30 CFTR‐interacting proteins identified from the MaMTH‐HTS pipeline (Table EV2). For these experiments, we used transiently expressed, V5‐tagged wt‐CFTR alongside transiently expressed, FLAG‐tagged preys in HEK293 cells. Of the 30 interactions tested, we were able to successfully confirm 17 (56%), a substantial proportion providing support for the biological relevance of our identified interactions (Appendix Fig S9A). Based upon our chloride conductance (Fig 3, Dataset EV6) and trafficking validation results (Fig 5), we also specifically examined the interaction of FGL2 with both wt‐ and F508del‐CFTR. Notably, we successfully confirmed the interaction by Co‐IP for both protein variants, providing further evidence that FGL2 is an important functional interactor of CFTR (Appendix Fig S9B).

Control blots for the co‐IP assays presented in Appendix Fig S9A and B, performed using unrelated GAPDH antibody, are presented in Appendix Fig S9C.

### Knockdown of FGL2 using lentivirus‐induced shRNA in patient‐derived organoid cultures

**Figure 6:**
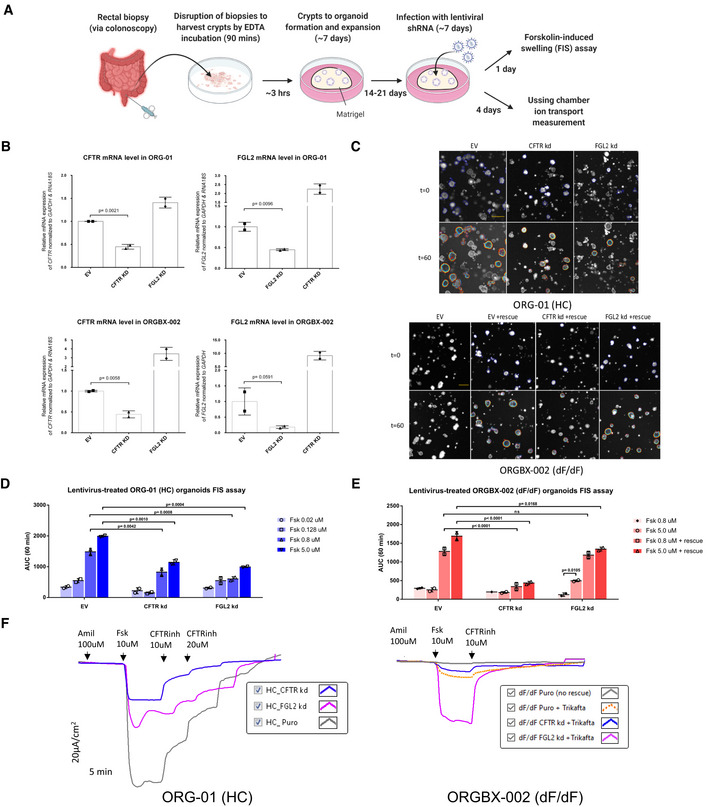
Effects of lentivirus‐mediated shRNA knockdown of the FGL2 and CFTR genes in intestinal organoids Schematic of patient‐derived intestinal organoid acquisition and expansion followed by two functional assays. Created with BioRender.com.qPCR Validation of lentivirus‐mediated shRNA knockdown of FGL2 and CFTR in intestinal organoids. Significance of sample differences was calculated using the one‐tailed student *t*‐test (*n* = 2 biological replicates). All values are mean ± SD. EV = empty vector.Representative confocal microscopy images of calcein green labelled FIS organoids of a healthy individual and a CF patient, before and 60 min after stimulation with Fsk (5 µM). Scale bar, 200 μm.Quantification of FIS response in wt‐CFTR organoids (ORG‐01(HC)) shown in (C) as area under the curve at 60 min. Significance of sample differences was calculated using two‐way ANOVA (*n* = 2 biological replicates). All values are mean ± SD.Quantification of FIS response in F508del‐CFTR organoids (ORG‐BX‐002 (dF/dF)) shown in (C) as area under the curve at 60 min. Significance of sample differences was calculated using two‐way ANOVA (*n* = 2 biological replicates). All values are mean ± SD.Tracing of Ussing chamber measurements of the transepithelial current produced by 2D monolayer cultures of specified organoids. The F508del‐homozygous monolayers with the knockdowns have been rescued with Trikafta.

In order to further explore the importance of FGL2 in CFTR biogenesis, traffic and function we performed lentivirus‐mediated shRNA knockdown of the FGL2 and CFTR genes in patient‐derived intestinal organoids and examined their effects using two functional readouts: the 3D‐organoid Fsk‐induced swelling (FIS) assay (Dekkers *et al*, 2013) and Ussing chamber measurements of organoid‐derived 2D‐monolayers (Li *et al*, 2004; Molinski *et al*, 2017; Cao *et al*, 2018) (Fig 6A).

Intestinal epithelial organoids were embedded into an extracellular matrix (e.g. Matrigel) that reconstitutes a basement membrane and is indispensable for cell survival, proliferation and maintenance of the 3D structure (Dekkers *et al*, 2013). However, this extracellular matrix prevents lentiviruses from binding to intestinal cells. We therefore applied an optimized version of an efficient gene transduction method reported previously (Van Lidth de Jeude *et al*, 2015) to infect 3D‐organoid cultures with lentivirus containing shRNAs targeting CFTR or FGL2. Using this approach, we were able to successfully knockdown each target gene in intestinal organoids derived from either healthy subjects or F508del homozygous CF patients (Fig 6B).

We next performed a Fsk‐induced swelling (FIS) assay on these 3D organoids to measure CFTR function (Dekkers *et al*, 2013). The FIS assay is well established as a relatively simple yet robust approach for the quantification of CFTR function using primary intestinal culture models that recapitulate essential features of the *in vivo* tissue architecture. Exposure of organoids to Fsk leads to a rapid increase in the levels of intracellular cAMP, resulting in opening of the CFTR channel, movement of chloride and fluid into the lumen and a consequent increase in organoid size (due to flow of water following the osmotic gradient) which is tracked in time‐course, live‐cell microscopy (Dekkers *et al*, 2013).

Using the FIS assay, we noticed a significant drop in the ability of organoids expressing wt‐CFTR to swell upon FGL2 knockdown, relative to empty vector control, comparable to the effects observed upon knockdown of CFTR (Fig 6C and D). Interestingly, this effect was not as strong in CF mutant organoids, where FGL2 knockdown only mildly reduced swelling (relative to empty vector control) upon Fsk stimulation in the cells treated with a combination of modulators VX‐661+VX‐445+VX‐770 (Trikafta^TM^) to rescue mutant CFTR activity (Fig 6C and E). This was in strong contrast to CFTR knockdown, which resulted in minimal swelling in CF mutant organoids in response to Fsk in the presence of Trikafta (Fig 6C and E). Additionally, FGL2 knockdown in CF mutant organoids led to increased swelling in response to Fsk in the absence of Trikafta rescue, an effect not observed in either empty vector or CFTR knockdown organoids (Fig 6C and E).

Finally, we examined the transepithelial currents in organoids grown in 2D‐monolayer using Ussing chambers, a well‐established method for studying epithelial ion transport (Li *et al*, 2004; Molinski *et al*, 2017; Cao *et al*, 2018) (Fig 6F). Consistent with the FIS data, knockdown of both CFTR and FGL2 in wt organoids led to a substantial decrease in the amount of Fsk‐stimulated CFTR channel activity (measured as a change in transepithelial current), relative to empty vector control (Fig 6F, left panel). In CF organoids, the effect of FGL2 knockdown was reversed, leading to greater CFTR channel activity in the presence of Trikafta than observed with Trikafta rescue alone (Fig 6F, right panel).

Overall, the results of both of these assays are intriguing, providing further evidence of a role for FGL2 in CFTR function and expression.

## Discussion

In this work, we focused on the systematic, high‐throughput mapping and characterization of the protein interactomes of wt‐ and F508del‐CFTR, with the goal of better understanding CFTR biogenesis/function and the cellular changes associated with the CF disease state. To accomplish this, we employed a modified form of our previously reported MaMTH assay (Petschnigg *et al*, 2014, 2017; Aboualizadeh *et al*, 2021). While the original MaMTH assay is well suited for mapping the interactions of the full‐length forms of complex membrane proteins (such as CFTR) directly in living cells, the arrayed nature of its screening format limits the number of interactions that can be screened in an efficient and cost‐effective manner. By introducing a number of changes, including a new fluorophore reporter system, we were able to adapt the system for effective screening of thousands of potential interactors simultaneously, in a pooled manner.

Using this new format, which we term MaMTH‐HTS, we were able to screen for PPIs using full‐length wt‐ and F508del‐CFTR “baits” against a library of close to 10,000 ORF “preys”, which we constructed from the Human ORFeome V8.1 collection (Yang *et al*, 2011). Between both baits, we identified a total of 447 candidate interactors, with 224 mapping to wt‐CFTR and 269 to F508del‐CFTR. Thirty‐one of these interactions were previously reported, with the remaining 416 representing new interactions with potential roles in CFTR function. A total of 46 interactors were shared between wt‐ and F508del‐CFTR. This overlap (~10.2%), is lower than that observed in other large‐scale CFTR interactome studies, which report a higher percentage of shared proteins (~60% overlap) constituting a “core interactome” containing many ER‐, Golgi‐ and trafficking‐associated members involved in CFTR biogenesis (Wang *et al*, 2006; Pankow *et al*, 2015). The differences in overlap observed are likely due to the distinct methodologies used, as the CFTR interactome has been mapped heavily by mass spectrometry‐based approaches in previous large scale studies (Lim *et al*, 2018), while our approach looks at interactions of full‐length CFTR directly in the membrane of live cells (an environment and context potentially allowing detection of distinct PPIs). Nevertheless, the 31 previously reported interactions confirmed by the MaMTH‐HTS help demonstrate the validity of our approach while expanding the scope of the CFTR interactome. Among the detected PPIs, we identified a subgroup of novel interactions for both for wt‐ and F508del‐CFTR (grey circle in Fig 2), which would not likely have been detected by hypothesis‐driven approaches. Not surprisingly, these are the proteins with the lowest density of connections, many corresponding to uncharacterized Open Reading Frames (ORFs), that is the so‐called dark matter of the genome (Guan & Lazar, 2019). Notwithstanding, these are also conceivably the most interesting PPIs, as they represent still unexplored pathways with the potential to lead to the discovery of novel druggable targets and biomarkers.

Based upon the differences between the traditional MaMTH and our modified MaMTH‐HTS format, we suspected that there may be variation in the types of interactions effectively mapped by each method. Comparison of the two approaches using a subset of wt‐ and F508del‐CFTR interactors identified in our MaMTH‐HTS screens revealed that this does appear to be the case, with only 20–25% of interactions reproducible in the traditional arrayed format. While some of these differential interactions could be reflective of background inherent in our MaMTH‐HTS method, functional testing of several of these revealed that they have a putative role in CFTR trafficking and, in the case of ZNF22, physical interaction was further confirmed by Co‐IP, suggesting that these differences are not always due to methodological specificities (summarized in Dataset EV8). Rather, it may indicate that the two methods may better identify different types of interactions, something which could result from steric differences in constructs, the use of stably expressed preys in MaMTH‐HTS versus transiently expressed preys in MaMTH, or even to sensitivity variations resulting from the use of two different reporter systems (i.e. a single *Gaussia princeps* luciferase readout versus the tagBFP/mCherry/eGFP selection), which could lead to detection of stronger versus weaker or stable versus transient interactions. We also observed that in traditional MaMTH testing of a subset of preys identified in MaMTH‐HTS as uniquely binding to one CFTR bait (wt‐ or F508del‐CFTR), about 20% also bound to the other CFTR bait. This may provide further support for complementary behaviour between the two assay formats, although technical issues with respect to prey coverage in MaMTH‐HTS cannot be ruled out and will be the target of future investigation as we continue to refine and benchmark the format.

To provide preliminary functional characterization of our interactome, we deployed a number of genetic and biochemical assays to address CFTR trafficking and channel activity. These included the FLIPR assay with membrane potential‐sensitive dyes (Ahmadi *et al*, 2017) to measure the effects of interactors on CFTR ion channel activity in cultured cells, targeted siRNA knockdown coupled with high‐content microscopy (Botelho *et al*, 2015) or western blotting to study the role of interactors in CFTR trafficking, co‐IP to orthogonally validate selected interactions, and FIS assays (Dekkers *et al*, 2013) and Ussing chamber (Li *et al*, 2004) measurements to examine effects of interactor knockdown in patient derived organoids. Although these functional assays only begin to address the potential wealth of information obtainable from our new interactome, we did identify evidence supporting interesting links between some interactors and CFTR function. This dataset also constitutes a valuable resource for multiple future studies to unravel mechanisms of disease in CF and CFTR‐related conditions.

The fact that some siRNAs targeting the same gene gave ambiguous results (one with a positive, another with a negative effect) can be due to several reasons. In fact, not all siRNAs target the gene they are aimed at with the same efficiency and some lack specificity. Based on data from multiple high‐content screens, it has been estimated that only ~30% of siRNAs effects can be confirmed (Neumann *et al*, 2010; Simpson *et al*, 2012; Almaça *et al*, 2013).

One interesting example of a CFTR PPI is ST6GALNAC1 (ST6 N‐Acetylgalactosaminide alpha‐2,6‐sialyltransferase 1), which was identified as an interactor of both wt‐ and F508del‐CFTR in MaMTH‐HTS. Notably, these interactions were also detected in our traditional arrayed MaMTH luciferase assay and the interaction with wt‐CFTR was validated by co‐IP. Overexpression of ST6GALNAC1 also had a modest effect on improving the functional expression of the F508del‐CFTR as seen in the FLIPR assay. ST6GALNAC1 is a sialyltransferase involved in the Golgi transport pathway and plays a role in post‐translational modification of lung mucins. A recent study reports an increase in the sialylation of lung mucins in CF pigs, and suggests that this could be linked to an increase in subsequent *Pseudomonas aeruginosa* adhesion and impaired mucociliary transport (Caballero *et al*, 2021). The authors suggest that lowering the amount of sialylated molecule linked to the lung mucin is worth investigating as a new CF therapeutic strategy for treating bacterial infections. Since we detected a physical interaction between CFTR and ST6GALNAC1, it is interesting to speculate that the altered molecular properties of mucins in the CF pig model could be due to a disruption or modulation of this interaction, something which should be investigated in future studies.

We also identified FKBP6 as a novel interactor of both wt‐ and F508del‐CFTR and found that FKBP6 overexpression led to a modest increase in the function of F508del‐CFTR in the FLIPR assay. This finding further supports the growing evidence of a regulatory module formed between ABC transporters and FKBP/HSP90 chaperones responsible for an early step of ER quality control and post‐ER trafficking (Wang *et al*, 2006; Geisler & Hegedűs, 2020).

Interestingly, siRNA that targets CAPZB led to a significant decrease in trafficking of both wt‐ and F508del‐CFTR. CAPZB encodes the beta subunit of the barbed‐end actin‐binding protein, and it functions in regulating actin filament dynamics and stabilization. Although we only found it interacting with mutant CFTR in MaMTH‐HTS, it did interact with both wt‐ and F508del‐CFTR in the traditional arrayed MaMTH assay, and overexpression of CAPZB led to an increase in the functional expression of both CFTR variants in FLIPR assays. These results support a role for CAPZB as a key player involved in CFTR and cytoskeletal interaction and acting as a promoter of wt‐CFTR trafficking to the PM under EPAC1 activation (Santos *et al*, 2020). Maintaining the CFTR interaction with CAPZB protein thus appears to be critical in promoting CFTR stability at the PM.

Another interesting interactor is VAPA, a type IV integral ER membrane protein in the vesicle‐associated membrane protein (VAP) protein family. VAPA is involved in membrane vesicle trafficking by interacting with the SNARE complex. It is also known to be involved in the unfolded protein response (UPR) and to also associate with the cytoskeleton. VAPA interacted only with wt‐CFTR in our MaMTH‐HTS screen, though it did show an interaction with the mutant in arrayed MaMTH. Interestingly, its siRNA led to a significant decrease in wt‐CFTR trafficking, while its overexpression led to increased function of F508del‐CFTR in the FLIPR assay, indicating a potentially important role in CFTR trafficking and suggesting that enhancement of VAPA interaction with mutant CFTR could serve as an additional therapeutic avenue to be further explored. It is worth mentioning that one of VAPA’s protein family members, VAP, has been reported to play a role in CFTR biogenesis serving as intracellular receptors that couple lipid homeostasis to proteostasis regulation (Ernst *et al*, 2016; Häsler *et al*, 2020). XAGE3 is another wt‐CFTR interactor detected in MaMTH‐HTS that displays a functional profile in our assays similar to VAPA. Although not much is known about its function aside from its involvement in lung cancer (Zendman *et al*, 2002; Nakagawa *et al*, 2005), it will be interesting to further explore this interaction given the putative role that wt‐CFTR plays in protection from epithelial‐mesenchymal transition (EMT) and cancer (Amaral *et al*, 2020).

Perhaps the most important functional interactor uncovered from this study is FGL2 (Fibrinogen Like 2, also known as Fibroleukin), whose interaction with both wt‐ and F508del‐CFTR we orthogonally validated using co‐IP. This is a 439 amino acid protein belonging to the fibrinogen‐related superfamily. It exists as both a type II transmembrane protein found on the surface of macrophages and endothelial cells, and a secreted protein by T cells (Marazzi *et al*, 1998) and it has been associated with liver, interstitium and renal fibrosis by facilitating macrophage polarization (Foerster *et al*, 2010; de Ridder *et al*, 2016; Wu *et al*, 2020). The membrane bound FGL2 has been shown to possess a serine protease activity, cleaving prothrombin to thrombin leading to fibrin deposition and thrombosis and was originally found to be implicated in the pathogenesis of fulminant hepatitis in humans (Levy *et al*, 2000) and has been used as a biomarker of severe liver diseases, including cancer (Foerster *et al*, 2010; Manns *et al*, 2017; Liu *et al*, 2021). Though little is known about the immunoregulatory properties of FGL2, its expression also seems to play a critical role in the pathogenesis of allograft rejection (Ning *et al*, 2005; Bézie *et al*, 2015) and thus in innate immunity. The soluble FGL2, on the other hand, has been shown to inhibit both T cell proliferation and maturation of bone marrow‐derived dendritic cells (Chan *et al*, 2003). Most interestingly, FGL2 transcripts showed a 1.6‐fold increase in CFTR‐KO mice versus their littermate controls (Guilbault *et al*, 2006).

In our current study, the PPI we observe is most likely between CFTR and the membrane‐bound form of FGL2, as the MaMTH system is not suited for detection of secreted proteins (although association with the secreted protein form during trafficking cannot be ruled out). Overexpression of FGL2 in our validation assays led to an enhancement of both wt‐ and F508del‐CFTR activity as measured in the FLIPR membrane potential assays. Additionally, knockdown of the gene led to a decrease in trafficking of wt‐CFTR to the PM, though an effect on the mutant was not observed, given it is already absent from the PM. In our intestinal organoid model, knockdown of FGL2 also resulted in a decrease in wt‐CFTR channel activity (as measured in both FIS and Ussing chamber assays), consistent with the observed reduction in trafficking observed in our siRNA analysis. However, our results in CF patient organoids were more complex, since FGL2 knockdown apparently did not significantly affect the activity of Trikafta‐rescued F508del‐CFTR in 3D organoids (as measured in the FIS assay) but lead to a substantial increase in activity in 2D monolayers (as measured in using Ussing chamber). Though we cannot rule out the possibility of the difference between 2D monolayer and 3D organoid models, this observed differential effect could be due to the occurrence of FGL2 transcripts in CF versus non‐CF tissues (Guilbault *et al*, 2006). It is worth noting that the effects on CFTR transcript levels upon knockdown of FGL2 and vice versa (Fig 6B) suggests some interplay at the transcriptional level is also present.

Altogether, these results suggest a role of the physical interaction of FGL2 with CFTR in its biogenesis and function, and it appears to be clearly important for proper expression of wt‐CFTR at the PM. The biology underlying its pronounced effects on mutant CFTR is less clear; however, and it will need to be further investigated. Overall, however, FGL2’s reported role in fibrosis and its growing interest as a therapeutic target in treating autoimmune disorders and transplant rejection make it one of the more interesting novel interactors of CFTR uncovered in this study and additional research to determine its potential for use as a therapeutic target in CF seems warranted.

In summary, through the use of MaMTH‐HTS, a newly developed high‐throughput variant of our MaMTH protein interaction screen designed for integral‐ and membrane‐associated proteins, we were able to successfully map interactomes of wt‐CFTR and its primary mutant F508del‐CFTR using a library consisting of > 10,000 human ORFs derived from the Human ORFeome V8.1 collection. Alongside the previous study on mapping of the F508del‐CFTR interactome using a deep proteomic analysis method (CoPIT)(Pankow *et al*, 2015), the results shown in this work represent one of the largest interactome studies of the wt‐CFTR and F508del‐CFTR proteins and should be a valuable resource for CF research. The MaMTH‐HTS approach revealed a wide range of new interactions not previously detected by other methods, some of which we demonstrate to have potentially important functional roles in CFTR regulation and biogenesis. Hopefully, the data provided here will prove to be useful for further studies into CF pathophysiology and for the development of innovative biomarkers and better treatment options. It is important to stress, however, that MaMTH‐HTS is still new, and that possible issues with background and sensitivity must be acknowledged when evaluating screening results, though such issues will continue to be explored and addressed as the method undergoes further benchmarking, refinement and application to a greater range of targets. Nevertheless, our results with CFTR clearly demonstrate the ability of the assay to identify novel interactions of biological importance, and we strongly believe that network biology approaches, such as MaMTH‐HTS, are increasingly important tools for obtaining unbiased insight into the aetiology of disease. We look forward to the future application of these technologies to further research and drive advancements in human health and disease.

## Materials and Methods

### Reagents and Tools table

**Table:** 

Reagent/Resource	Reference or Source	Identifier or catalogue number
**Experimental models**		
HEK‐293 Flp‐In cells (*H. sapiens*)	ThermoFisher	R75007
HEK‐293T cells (*H. sapiens*)	ATCC	CRL‐3216
Electrocompetent E. cloni cells (10G ELITE)	Lucigen	60052‐4
CFBE cells (*H. sapiens*)	Molinski *et al* (2015) Botelho *et al* (2015)	N/A
**Recombinant DNA**		
MaMTH reporter vector using the pcDNA3.1(−) backbone	Gibson *et al* (2009) and Saraon *et al* (2020)	N/A
MaMTH bait destination vector	Gateway cloning technology (Thermo Fisher) and Saraon *et al* (2020)	N/A
wt‐ and F508del‐CFTR cDNA entry constructs	Dr. Johanna Rommens lab	Ref Seq: NM_000492.3
MaMTH prey destination vector	Saraon *et al* (2020)	N/A
Plasmid library generation	Human ORFeome V8.1 collection (Yang *et al*, 2011)	N/A
**Antibodies**		
Anti‐Flag antibody	Sigma	F‐1804
Anti‐mouse IgG antibody conjugated with Alexa 647	Invitrogen	A‐31571
Anti‐CFTR 596	Cystic Fibrosis Foundation	N/A
Anti‐calnexin	BD Transduction Laboratories	610523
HRP‐conjugated goat anti‐mouse IgG	Bio‐Rad	170‐6516
Anti‐FLAG antibody	Millipore Sigma	F3165
Anti‐V5 antibody	Cell Signaling Technology	13202S
**Oligonucleotides and other sequence‐based reagents**		
PCR primers	This study	Table EV1
Custom library of 421 Ambion Silencer Select siRNAs	This study	Table EV3
Non‐targeting Neg1 negative control siRNA	Ambion	4390844
**Chemicals, enzymes and other reagents**		
DMEM	Wisent	319‐005‐CL
DMEM high glucose	Corning	10‐013‐CV
Opti‐MEM	Gibco	31985070
IntestiCult^TM^ Human OGM	STEMCELL Technologies	06010
FBS	Gibco	10270
Hygromycin	Bioshop	HYG003.10
Blasticidin	Invivogen	ant‐bl
Puromycin	Invivogen	ant‐pr
Doxycycline	Sigma	9891
Primocin	Invivogen	ant‐pm
Vancomycin	Sigma	SBR00001
Gentamycin	Sigma	G1272
Y‐2632 (ROCK pathway inhibitor)	Sigma	SCM075
Forskolin	Sigma Aldrich	F3917
VX‐661	Selleckchem	S7059
VX‐809	Selleckchem	S1565
VX‐445	Selleckchem	S8851
CFTRinh‐172	Cystic Fibrosis Foundation EMD Millipore Corp	N/A
Amiloride	Spectrum Chemical	TCI‐A2599
Phire Tissue Direct Dilution Buffer	Thermo Scientific	N/A
Phire Tissue Direct PCR Master Mix	Thermo Scientific	N/A
QIAquick PCR Purification Kit	Qiagen	N/A
Qiagen Mini Kit	Qiagen	27106
Qiagen Micro Kit	Qiagen	74004
iSCRIPT cDNA synthesis kit	Bio‐Rad	1708890
Ssofast^TM^ EvaGreen fluorophore	Bio‐Rad	172‐5200
CellMask	Invitrogen	H32712
ER‐Tracker	Invitrogen	E34250
Hoechst 33342	Sigma	B2261
Calcein green	Invitrogen	C34852
X‐tremeGENE 9 DNA Transfection Reagent	Roche	6365787001
Lipofectamine 2000	Invitrogen	11668019
Lipofectamine LTX	Thermo Fisher Scientific	15338100
Laemmli sample buffer	Bio‐Rad	161‐0747
Protease inhibitor cocktail	Roche	11873580001
Clarity Western ECL Substrate	Bio‐Rad	1705061
Blue membrane potential dye	Molecular devices	R8034
384 well plates	Falcon	736‐2044
96 well plates	Sarstedt	83.3924
12 well plates	SPL Life Sciences	30012
PureCol^®^‐coated Transwell	Corning	Costar^TM^ 3470
Non‐perfused Ussing chamber	Physiologic Instruments	N/A
Drying pearls	Sigma‐Aldrich	94098
μMACS^TM^ FLAG magnetic microbeads and columns	Miltenyi Biotec	N/A
Matrigel	BD Biosciences	N/A
**Software**		
GraphPad Prism	https://www.graphpad.com	N/A
Integrated Interactions Database (IID)	PMID: 34755877 http://ophid.utoronto.ca/iid	Version 2021‐05
NaViGaTOR	http://navigator/ophid.utoronto.ca/navigatorwp	Version 3.0.15
R programming language/Bowtie 2 Alignment Tool	R Core Team, Bowtie2 alignment tool (Langmead & Salzberg, 2012)	N/A
CellProfiler	McQuin *et al* (2018)	N/A
Image Lab	Bio‐Rad	N/A
MATLAB‐based organoid swelling capturing program	MATLAB	N/A
**Other**		
NexteraXT library preparation kit	Illumina	N/A
NextSeq500 System	Illumina	N/A
HiSeq2500 System	Illumina	N/A
BD FACS Melody	BD Biosciences	N/A
Rank product method	Breitling *et al* (2004)	N/A
Genevac miVac Duo	Thermo Fisher Scientific	N/A
Multidrop^TM^ Combi peristaltic dispenser	Thermo Scientific	5840300
Leica DMI 6000B	Leica	N/A
ChemiDoc XRS	Bio‐Rad	N/A
Cellomics ArrayScan, VTI HCS Reader	Thermo Fisher Scientific	N/A
NanoDrop 2000	Thermo Fisher Scientific	N/A

### Methods and Protocols

#### Generation of MaMTH‐HTS reporter cell line

Reporter vector was generated in a pcDNA3.1(−) backbone using ORFs expressing eGFP under the control of a 5xGAL4 UAS and puromycin resistance marker under the control of a constitutive PGK promoter, via Gibson assembly (Gibson *et al*, 2009). This vector was then stably integrated into FLP‐compatible HEK293 cells and the candidate line displaying the best MaMTH‐responsive reporter activity (with minimal background) was selected, following our previously described protocol used for generation of *Gaussia princeps* luciferase reporter lines (Saraon *et al*, 2020).

#### Generation of MaMTH CFTR bait constructs

CFTR bait constructs were generated using Gateway cloning technology (Thermo Fisher) and destination vector designed in our lab, modified from our previously reported A1160 vector(Saraon *et al*, 2020). These new constructs retained the general structural features of the original vector (including tetracycline induction and Flp‐In T‐REx compatibility) but also expressed P2A “linked” tagBFP to allow for fluorescence‐based detection of bait expression (Appendix Fig S2A). wt‐ and F508del‐CFTR cDNA entry constructs (Ref Seq: NM_000492.3) used in cloning were generously gifted from Dr. Johanna Rommens.

#### Generation of MaMTH Prey construct and library

Gateway‐ and CRISPR‐compatible MaMTH prey destination vector was designed, modified from our previously reported A1245 vector (Saraon *et al*, 2020). This new construct contained the primary features of the earlier plasmid (including P2A “linked” mCherry to allow for fluorescence‐based detection of prey expression), alongside a tetracycline‐inducible promoter, hygromycin resistance for selection upon stable integration and CRISPR‐targeting sites (Appendix Fig S2B).

For plasmid library generation, 33 ORF entry clone pools [generated from the Human ORFeome V8.1 collection (Yang *et al*, 2011), were transferred into this prey vector construct using Gateway cloning (Thermo Fisher). LR reactions were carried out on a per pool basis and transformed into commercial electrocompetent E. cloni cells (Lucigen) to maximize efficiency. Colony counts from transformations were used to estimate coverage of individual pools, which were determined to be a minimum of 1,000× in all cases. ORF representation in pools was verified by deep sequencing using NexteraXT library preparation and sequencing on a NextSeq500 system (150 bp single read).

#### Generation of MaMTH prey cell line library pools

For generation of MaMTH‐HTS cell line library stably expressing preys, MaMTH‐ORF‐expressing prey plasmids were transfected, on a per pool basis, into HEK293 MaMTH‐HTS eGFP reporter cells using X‐tremeGene 9 (Roche) alongside plasmids expressing Cas9, AAVS targeting gRNA and prey‐plasmid targeting gRNA. The cells were grown for 3 days at 37°C /5% CO_2_ in DMEM/10% FBS/1% PenStrep prior to expansion into DMEM/10% FBS/1% PenStrep media containing 100 µg/ml of hygromycin. Plates were grown further until foci appeared (approximately 7 days), at which point all foci were harvested collectively per pool, expanded further and frozen/stored in N_2_(l). Foci counts were used to ensure that pool coverage was at least 30× or greater. To establish ORF representation in cell line pools, genomic DNA was isolated on a per pool basis, amplified by PCR using cassette‐specific primers (Table EV1, Primer Set 1) and deep sequenced using NexteraXT library prep and an Illumina HiSeq2500 system (150 bp, single read, Dataset EV1).

#### MaMTH‐HTS screens

1. Plasmid expressing wt‐CFTR or F508del‐CFTR “Bait” protein fused at its C‐terminus to MaMTH‐HTS Bait tag (Cub‐GAL4TF‐P2A‐tagBFP) was transfected into a pooled “Prey” library of HEK293 MaMTH reporter cell lines (containing chromosomally integrated GFP reporter under the control of a GAL4TF promoter) stably expressing members of the Human ORFeome V8.1 collection (~10,000 ORFs) fused at their N‐terminus to MaMTH‐HTS Prey tag (Nub) and at their C‐terminus to P2A‐mCherry. Transfections were performed using Xtreme gene 9 transfection reagent (Roche), following manufacturer’s protocols, in 6 cm dishes containing 1,000,000 seeded cells per plate (grown overnight to ~80% confluency) with 750 ng of bait DNA.
2. Five hours post transfection 0.5 µg/ml Tetracycline was added (to induce Bait and Prey expression) and the cells were grown for 2 days at 37°C/5% CO_2_ in DMEM/10% FBS/1% PenStrep media.
3. Following growth, the cells were harvested by trypsinization and resuspended in Basic Sorting Buffer (1 × PBS, 5 mM EDTA, 25 mM HEPES pH 7.0, 1% BSA) at a concentration of 1–2 × 10^6^ cells/ml.
4. The cells were then sorted by Flow Cytometry using a BD FACS Melody (BD Biosciences). Sorts were performed such that the cells were sequentially selected based on the presence of mCherry fluorescence (indicating Prey expression), tagBFP fluorescence (indicating Bait expression) and finally GFP fluorescence (indicating Bait‐Prey interaction). The cells were collected in DMEM/25% FBS.
5. Collected cells were centrifuged at 200 *g* for 5 min and pellets resuspended/processed in Phire Tissue Direct Dilution Buffer (Thermo Scientific).
6. ORFs were then amplified using Phire Tissue Direct PCR Master Mix (Thermo Scientific) following manufacturer’s instructions with the primer sets shown in Table EV1, generating two technical replicates (to use for determining possible primer‐specific PCR biases or inconsistencies across sequencing samples).
7. PCR products were then purified using a QIAquick PCR Purification Kit (Qiagen).
8. Purified PCR products were subjected to Nextera XT library preparation (following manufacturer’s instructions) and deep sequenced on an Illumina HiSeq2500 system (150 bp single read).
9. Sequencing data was then processed with in‐house software scripts developed using the R programming (R Core Team) language and integrating the Bowtie2 alignment tool (Langmead & Salzberg, 2012), removing any ORFs with total counts less than 3 in both replicates, to generate lists of candidate interactors.

#### Identifying frequent fliers

Frequent fliers were identified by using the rank product method (Breitling *et al*, 2004) to analyse results of 9 MaMTH‐HTS screens of control baits from distinct classes of membrane proteins. The main idea was that frequent fliers would have strong interaction signals from multiple screens; the rank product method (Breitling *et al*, 2004) would calculate the probability of such signals occurring by chance—thus providing a *P*‐value for the hypothesis that a prey is a frequent flier. Interaction signal was considered proportional to read counts per million (CPM) from deep sequencing.

The rank product method (Breitling *et al*, 2004) comprised calculation of rank products, *P*‐values, and adjusted *P*‐values for all preys. Rank products were calculated by ranking CPMs in each screen (the highest CPM receiving a rank of 1) and then for each prey, calculating the geometric mean of its 9 CPM ranks.

*P*‐values were calculated by determining how often rank products were better than from randomized data. This was done through 100,000 iterations of the following steps: CPM rank data were randomized (i.e. CPM ranks in each screen were randomly permuted), rank products were calculated from the randomized data and compared against observed rank products. *P*‐values were calculated as the number of times rank products from randomized data were smaller than from observed data, divided by 100,000.

*P*‐values were adjusted for multiple testing by the Benjamini‐Hochberg method (Benjamini & Hochberg, 1995).

#### Annotation of CFTR interactions with known and predicted interactions

Previously detected and predicted CFTR interactions were retrieved from the Integrated Interactions Database (IID) (Kotlyar *et al*, 2021) version 2021‐05 (http://ophid.utoronto.ca/iid). CFTR interaction partners were annotated with GO Slim cellular components and molecular functions from the GOA database (Huntley *et al*, 2015) (downloaded 2021‐06‐16), diseases from DisGeNET (Piñero *et al*, 2020) version 7.0, tissues from IID (Kotlyar *et al*, 2021) version 2021‐05, and pathways from pathDIP (Rahmati *et al*, 2019) version 4.0 (http://ophid.utoronto.ca/pathDIP). CFTR interactions were annotated with mutations from IntAct (Del‐Toro *et al*, 2019; Porras *et al*, 2020) version 4.2.17. Annotated interaction data are presented in Dataset EV4.

Network figures were created using the integrated interaction data in NAViGaTOR ver. 3.0.15 (Brown *et al*, 2009); http://navigator.ophid.utoronto.ca/navigatorwp). Final networks were exported in SVG file format, finalized in Adobe Illustrator ver. 26.0.1 to include legends, and exported in 300 DPI PNG format for publication.

#### Fluorescence ‐microscopy to monitor CFTR localization

HEK293 cells were transiently transfected with plasmid expressing wt‐CFTR or F508‐del CFTR fused to MaMTH tag (C‐terminus of ubiquitin and LexA artificial transcription factor) and GFP and grown at 37°C/5% CO_2_ in DMEM/10% FBS/1% PenStrep media. Following growth, the cells were visualized by fluorescence microscopy using CellMask (Invitrogen) to stain the plasma membrane and ER‐Tracker (Invitrogen) to stain the endoplasmic reticulum, following standard protocols.

#### Traditional MaMTH assay

Protocols for preparing and performing the MaMTH assay have been previously published (Saraon *et al*, 2017). All the MaMTH data presented are from three independent trials each with at least three technical replicates in a 96‐well plate format, unless otherwise stated.

#### Membrane potential assay

HEK293 cells were grown on 96 well tissue culture plates until confluent. The blue membrane potential dye (Molecular devices) was dissolved at a concentration of 0.5 mg/ml as reported previously (Molinski *et al*, 2015; Ahmadi *et al*, 2017). After 15–20 min of loading the dye at 37°C, 5% CO_2_ and humidified air, the plate was transferred to the microplate reader (Molecular devices). Briefly, the reader was heated to 37°C and the settings for fluorescence whole well scan were turned on, with multiple points being read each well. Upon start of the experiment, baseline reads of at least 4–5 scans were made, followed by addition of drugs (2.5 µl/well). After addition of each drug again 4–5 scans were done. The experiment ended by addition of inhibitor (CFTRinh‐172 10 μM). Upon completion of experiment, the data were exported and analysed.

#### Cell lines for trafficking assays and western blots

CFTR traffic measurements were performed on Cystic Fibrosis Bronchial Epithelial (CFBE) cells expressing inducible (TET‐ON) double‐tagged CFTR (Molinski *et al*, 2015) traffic reporters (mCherry‐Flag‐CFTR) previously described (Botelho *et al*, 2015) and representing either the WT‐ or F508del‐CFTR variants. Western blot validation experiments were performed on CFBE cell lines constitutively expressing wt‐ or F508del‐CFTR.

#### Cell culture for trafficking assays and western blots

CFBE mCherry‐Flag‐CFTR cells (WT or F508del variants) were cultured in DMEM high glucose (Corning 10‐013‐CV) supplemented with 10% fetal bovine serum (Gibco 10270), 10 μg/ml of blasticidin (Invivogen #ant‐bl) and 2 μg/ml of puromycin (Invivogen #ant‐pr‐1) at 37°C and 5% CO_2_. CFBE cells constitutively expressing WT‐/F508del‐CFTR were cultured in equivalent conditions but excluding blasticidin. When seeded to siRNA coated multi‐well plates culture media was DMEM supplemented with FBS (0.1% when using VX‐661, 10% elsewhere).

#### Preparation of siRNA‐coated multi‐well plates

Multi‐well plates were coated with a custom library of 421 Ambion Silencer Select siRNAs (Table EV3) The library targeted 210 MaMTH‐HTS hits (2 siRNAs for most genes) and included the non‐targeting Neg1 negative control (Ambion 4390844) and CFTR (s2947) and INCENP (s7424) transfection controls. The library was used to coat multi‐well plates for reverse transfection, according to a previously reported protocol (Botelho *et al*, 2015). For the CFTR traffic screen 384 well plates (Falcon 736–2,044) and for western blot assays 12 well plates (SPL Life Sciences 30012) were used. Briefly, siRNAs were diluted to 3 µM with RNase free water. In parallel, a transfection mix comprised of 0.4 M of sucrose in Opti‐MEM (Gibco 31985070), RNase‐free water and Lipofectamine 2000 (Invitrogen 11668019) in the proportions 1.715:1:1 and a 2 g/l of gelatin solution were prepared. Then, each siRNA solution was mixed with the transfection mix, incubated 20 min at room temperature and mixed with the gelatin solution (proportions 1:1.4:1.4). The resulting solution was then diluted 50‐fold in RNase‐free water. Finally, the solution was added to each well of the multi‐well plates (384 well: 15 µl/well, 0.24 pmol siRNA/well; 12 well: 720 µl/well, 11.4 pmol siRNA/well). Plates were immediately lyophilized on a speedvac (Genevac miVac Duo) at 37°C for about 11 h and then stored in air‐tight boxes containing drying pearls (Sigma‐Aldrich 94098) at room temperature until needed for bioassays. Traffic assay plates contained 7–18 negative control wells (Neg1). Western blot plates contained 1 negative control sample.

#### CFTR traffic assay

The assay was performed as previously described (Botelho *et al*, 2015) CFBE cells expressing the inducible mCherry‐Flag‐CFTR reporter (WT‐ or F508del‐CFTR variants) were grown to confluence and split to 50% confluency. Twenty‐four hours later, the cells were trypsinized to antibiotic‐free medium and seeded in siRNA coated 384‐well plates (1,000 cells/well) using a Multidrop™ Combi peristaltic dispenser (Thermo Scientific 5840300). CFTR expression was induced for 48 h (24 h after seeding) by supplementing the medium with 1 μg/ml of doxycycline (Sigma 9891). At this point, VX‐809 (3 µM) or VX‐661 (5 µM) (Selleckchem (S7059)) were added to selected wells containing Neg1 siRNA as a positive control for F508del‐CFTR traffic rescue. Extracellular Flag tags were immunostained in non‐permeabilized cells 72 h after seeding. The primary anti‐Flag antibody (Sigma F‐1804, 1:500) was incubated 1 h at 4°C, the cells were fixed with PFA 3% for 20 min at 4°C, an anti‐mouse IgG antibody conjugated with Alexa 647 (Invitrogen A‐31571. 1:500) was incubated 1 h at room temperature and Hoechst 33342 (200 ng/ml, Sigma B2261) was incubated 1 h at room temperature. Four independent biological replicates were performed.

#### High‐content image acquisition and analysis

Automated imaging was performed on a Leica DMI 6000B widefield fluorescence microscope using a 10×/0.40 objective, EL6000 light source, Hamamatsu Orca‐Flash4.0 camera and suitable filter cubes for Hoechst, mCherry and Alexa 647. Each well was imaged at 4 sub‐positions. Images were annotated with experimental metadata using the htmrenamer R package (Hagemeijer *et al*, 2020). Cell‐based fluorescence quantification was performed with CellProfiler (McQuin *et al*, 2018) in order to quantify CFTR expression (mCherry) or PM localization (Alexa 647), excluding the cells presenting apoptotic or otherwise aberrant nuclei. Numerical data was processed and normalized with a custom R script, which excluded the cells with negligible CFTR expression, out‐of‐focus images or images with less than 100 cells. For each image, the median Alexa 647 fluorescence intensity across all the cells was computed and converted into a *Z*‐score using the median 5 × 5 well neighbourhood fluorescence as reference. The reported *Z*‐score is the median value across replicate wells and experimental replicates. Screen hits were the siRNAs, which yielded an increase in PM CFTR of *Z*‐score > +1 or decrease in PM CFTR of *Z*‐score < −1.

#### Western blotting (WB)

CFBE cells constitutively expressing WT‐ or F508del‐CFTR were grown to confluence and split to 50% confluency. Twenty‐four hours later, the cells were trypsinized to antibiotic‐free medium and seeded in siRNA coated 12‐well plates (87,500 cells/well). Twenty‐four hours later the media was changed to DMEM supplemented with 0.1% FBS containing wither 0.05% DMSO or 5 µM of VX‐661. After an additional twenty‐four hours, the cells were lysed and subjected to SDS‐PAGE. A sample buffer supplemented with reducing Laemmli sample buffer (Bio‐Rad 161‐0747), protease inhibitor cocktail (Roche 11873580001), benzonase and MgCl_2_ was used. Samples were resolved in a 10% SDS‐PAGE gel and transferred into a PVDF membrane. Membrane was blocked with 3% (w/v) non‐fat milk (NFM) in tris buffered saline with 0.1% Tween 20 (TBS‐T) for 1 h at room temperature and incubated overnight at 4°C with anti‐CFTR 596 (Cystic Fibrosis Foundation) or anti‐calnexin (BD Transduction Laboratories 610523) diluted 1:3,000 in 3% (w/v) NFM/TBS‐T. Membranes were then incubated with HRP‐conjugated goat anti‐mouse IgG (Bio‐Rad 170‐6516) diluted 1:3,000 in 3% NFM/TBS‐T for 2 h at room temperature. Subsequently, the immunoreactive signals were detected using a Clarity Western ECL Substrate (Bio‐Rad 1705061) and recorded with a ChemiDoc XRS (Bio‐Rad). Densitometric analysis was performed on Image Lab (Bio‐Rad). The intensity of CFTR bands was always expressed as a fold change versus the calnexin internal control. Statistical analysis consisted of a one‐way ANOVA versus the siNeg1 negative control treated with DMSO followed by Dunnett’s post hoc test. An equivalent analysis was performed for F508del‐CFTR cells treated with siNeg1 and VX‐661. The significance level was 0.05.

#### Co‐immunoprecipitation (Co‐IP)

Co‐IP validation was performed on a subset of top CFTR interactors uncovered via MaMTH‐HTS (*n* ≥ 2 biological replicates). Briefly, 293T cells were grown in standard high‐glucose Dulbecco’s modified Eagle medium (DMEM) containing 4 mM of l‐glutamine, 1 mM of sodium pyruvate, 10% fetal bovine serum (FBS), penicillin (100 U/ml) and streptomycin (100 μg/ml) under standard conditions (37°C, 5% CO_2_) as previously reported (PMID: 31536960, 29128334). The cells were co‐transfected with V5‐tagged CFTR and FLAG‐tagged preys using Lipofectamine LTX (Thermo Fisher Scientific #15338100), and incubated for 48 h prior to harvesting the cells for Co‐IP. Afterwards, the cells were crosslinked for 30 min at room temperature using 0.5 mM of dithiobis succinimidyl propionate (DSP) diluted in PBS. After quenching the crosslinked reaction with the addition of 100 mM of Tris‐HCl (pH 7.5) for 10 min at room temperature, the cells were collected by gentle pipetting and centrifuged at 1,300 *g* for 5 min. The crosslinked pellets were then lysed in 1 ml of RIPA buffer (50 mM of Tris HCl pH7.5, 150 mM of NaCl, 1% Sodium deoxycholate, 0.1% SDS, 1% NP‐40, 1 mM of EDTA), containing 1× protease inhibitor cocktail and agitated for 1 h at 4°C. About 100 μl of μMACS DYKDDDDK (also known as FLAG) magnetic microbeads (Miltenyi) were added to the samples and then incubated for additional 4 h at 4°C in agitation. Microbeads suspension was passed through μMACS columns (Miltenyi), washed two times with 1 ml of 0.1% RIPA and 1× protease inhibitor cocktail, followed by another wash with 1 ml detergent free RIPA. Proteins were eluted using 100 μl of 2× Laemmli buffer heated at 95°C. Eluates were then analysed by immunoblotting using anti‐FLAG antibody (Millipore Sigma #F3165) directed against overexpressed FLAG‐tagged protein corresponding to the interactors, as well as using anti‐V5 antibody (Cell Signalling Technology #13202S) directed against the V5‐tagged CFTR.

#### Lentivirus‐mediated knockdown in the organoids

Lentiviral particles containing shRNA sequences specific for the target proteins were generated in HEK293T cells using the Mission shRNA system with validated shRNA sequences (Sigma‐Aldrich) following standard protocols from The RNAi Consortium (TRC) Broad Institute.

The protocol for lentivirus packaging, modified from a previously published method (Hotta *et al*, 2009) was generously provided by Nicolaes Min from Dr. Xi Huang’s Lab at The Hospital for Sick Children. Briefly, the virus‐containing supernatant media were harvested and filtered through a 0.45‐µM syringe filter to remove cell debris and to prevent contamination with virus producer cells. Viruses were then ultracentrifuged for 2 h at 90,000–100,000 *g* at 4°C. After the centrifugation and discarding of the supernatant, the viral pellets were resuspended in approximately 1 ml of Growth medium (IntestiCult^TM^ Human OGM, STEMCELL Technologies, CAN) and stored in −80°C freezer.

The protocol for lentiviral transduction of the patient‐derived intestinal organoids was based on previously published method (Van Lidth de Jeude *et al*, 2015) with minor modifications of scaling down the reagents from the original 48‐wells plate to the 24‐wells plate format.

Lentivirus production and infection is covered under approval from The Hospital of Sick Children (REB number: 1000058992) and all steps were performed in a biosafety level 2/3‐certified laboratory.

#### Quantitative real‐time PCR

RNA extraction was performed according to the manufacturer’s protocol (Qiagen Micro or Mini Kit) (Hilden, Germany). Briefly, the cells were lysed, RNA extracted and then RNA concentration was measured while using a NanoDrop 2000 instrument (Thermo Fisher Scientific, Waltham, MA, USA). Only samples with a concentration > 100 ng/µl were used, with a 260/280 ratio between 1.8 and 2.1. cDNA synthesis was performed while using reverse transcriptase (iSCRIPT cDNA synthesis kit, Bio‐Rad, Hercules, CA, USA) or without reverse transcriptase (negative control). Quantitative real‐time PCR was performed while using Eva green (Ssofast Evagreen, Bio‐Rad, Hercules, CA, USA) fluorophore in 96‐well plates (Bio‐Rad, Hercules, CA, USA) and then normalized to GAPDH or RNA18S.

#### Forskolin‐induced swelling (FIS) assay

The protocols for FIS assay including the crypt isolation and culturing of human intestinal cells in Matrigel have been described previously (Dekkers *et al*, 2013, 2016; Eckford *et al*, 2014). In short, intestinal crypts were isolated from rectal biopsies and seeded in 50% Matrigel (BD Biosciences) in 24‐well plates (~10–30 crypts in three 10‐μl Matrigel droplets per well). Growth medium (IntestiCult^TM^ Human OGM, STEMCELL Technologies, CAN) was supplemented with Primocin (1:500; Invivogen). Vancomycin, gentamicin (both from Sigma) and Y‐27632 (ROCK pathway inhibitor) were added during the first week of culture. The medium was refreshed every 2–3 days, and resulting organoids were passaged ~1:3–5 every 7–10 days.

Lentivirus‐infected rectal organoids were plated in 96‐well culture plates in 4 μl of 50% Matrigel containing 20–80 organoids per well and immersed in 100 μl medium. CFTR rescue was achieved by adding modulators (3 µM VX661^+^ 3 µM VX445, Selleckchem, Houston, TX, US) in the medium 18–24 h prior to completing the FIS assay. For the FIS assay, organoids were incubated for 30 min with 3 μM of calcein green (Invitrogen) before stimulation with Fsk concentration 0.2–5.0 µM (Sigma Aldrich) in addition to 3 µM VX770 in rescued cultures, and directly analysed by confocal live cell microscopy at 37°C (Cellomics ArrayScan VTI HCS Reader, ThermoFisher Scientific). The increase in swelling over time was captured using a MATLAB‐based software program and the area under the curve (AUC, *t* = 60 min; 2 technical replicates) was calculated using GraphPad Prism.

#### Ussing chamber experiment

The method of measuring the CFTR‐dependent intestinal epithelial ion transport properties in rectal organoid‐derived monolayers have been described previously (Li *et al*, 2004; Molinski *et al*, 2017; Cao *et al*, 2018; Zomer‐van Ommen *et al*, 2018). Briefly, upon dissociation of the 3D intestinal organoid cultures, the cells were grown into the 2D monolayer on PureCol^®^‐coated Transwell (Costar™ 3470, Corning, Tewksbury, US). They were studied in a non‐perfused Ussing chamber (Physiologic Instruments, San Diego, CA). The buffer was maintained at pH 7.4 and 37°C and continuously gassed with 5% CO_2_/95% O_2_ mix. The transepithelial potential (Vte) was recorded and the baseline resistance (Rte) was measured following repeated, brief short‐circuit current pulses (1 μA every 30 s). The results are presented as equivalent transepithelial current (Ieq), which was calculated using Ohm’s law. CFTR function was determined after inhibition of the epithelial sodium channel (ENaC) with amiloride in the apical bath (100 μM, Spectrum Chemical, Gardena, CA) and cAMP activation with forskolin (10 μM, Sigma‐Aldrich, US). CFTR activity was quantified as Ieq difference following forskolin stimulation, CFTRInh‐172 (10 μM, EMD Millipore Corp. US) was applied to inhibit CFTR activity.

#### Validation strategy

Our validation strategy first involved re‐testing a large subset of 240 of the 447 CFTR interactors identified from the MaMTH‐HTS screens (Fig 2) using the traditional array‐based MaMTH assay. From there, we selected 26 interactors based on their interaction signal strength and consistency in their interaction profile between the two methods. Note that we included both cases where traditional and HTS interactors overlapped and where they did not, focusing largely on interactions confirmed in traditional MaMTH for both WT and mutant (but including some instances where this was not the case for sample diversity). The 26 interactors were then tested in the fluorescence‐based FLIPR assay to monitor their effect on the channel activity. Concomitantly, we performed the traffic‐based siRNA microscopy screen, targeting 208 genes (based on their availability in the siRNA library) from our original 447 interactors to investigate the trafficking of CFTR to the plasma membrane in the absence of these discovered interactors. This high‐content screen was combined with Western blot experiments for validating the post‐translational glycosylation pattern of CFTR. From these experiments, we performed final orthogonal validation on some of the most interesting interactors using a co‐IP approach and followed up with organoid work. A graphical overview of our validation approach is provided in Appendix Fig S10.

#### Statement of informed consent

Informed consent was obtained prior to rectal biopsies using standard endoscopic biopsy forceps.

## Author contributions

**Sang Hyun Lim:** Conceptualization; Formal analysis; Validation; Investigation; Writing—original draft; Writing—review & editing. **Jamie Snider:** Conceptualization; Software; Formal analysis; Validation; Investigation; Methodology; Writing—original draft; Writing—review & editing. **Liron Birimberg‐Schwartz:** Formal analysis; Investigation. **Wan Ip:** Formal analysis; Investigation. **Joana, C Serralha:** Formal analysis; Investigation. **Hugo, M. Botelho:** Formal analysis; Supervision; Investigation; Writing—original draft; Writing—review & editing. **Miquéias Lopes‐Pacheco:** Formal analysis; Investigation. **Madalena, C Pinto:** Investigation. **Mohamed, Taha Moutaoufik:** Formal analysis; Investigation. **Mara Zilocchi:** Investigation. **Onofrio Laselva:** Formal analysis; Investigation. **Mohsen Esmaeili:** Investigation. **Max Kotlyar:** Data curation; Formal analysis; Visualization; Writing—original draft; Writing—review & editing. **Anna Lyakisheva:** Formal analysis; Investigation. **Priscilla Tang:** Formal analysis; Investigation. **Lucía López Vázquez:** Investigation. **Indira Akula:** Investigation. **Farzaneh Aboualizadeh:** Investigation. **Victoria Wong:** Investigation. **Ingrid Grozavu:** Investigation. **Teuta Opacak‐Bernardi:** Investigation. **Zhong Yao:** Conceptualization; Methodology. **Meg Mendoza:** Investigation. **Mohan Babu:** Supervision; Writing—original draft. **Igor Jurisica:** Software; Formal analysis; Visualization; Writing—original draft; Writing—review & editing. **Tanja Gonska:** Formal analysis; Supervision; Investigation. **Christine, E Bear:** Conceptualization; Supervision. **Margarida, D Amaral:** Supervision; Writing—original draft; Writing—review & editing. **Igor Stagljar:** Conceptualization; Supervision; Writing—original draft; Project administration.

In addition to the CRediT author contributions listed above, the contributions in detail are:

SHL: performed experiments, analysed data and wrote the manuscript. JS: performed experiments, analysed data and wrote the manuscript. LB‐S: performed experiments and analysed data. WI: performed experiments and analysed data. JCS: performed experiments and analysed data. HMB: supervised research, planned, performed and analysed experiments. Contributed to final manuscript. ML‐P: performed experiments and analysed. MCP: performed experiments. MTM: performed experiments and analysed data. MZ: performed experiments. OL: performed experiments and analysed data. ME: performed experiments. MK: performed data analysis and interactome assembly. AL: performed and assisted with experiments, assisted with data analysis. PT: performed and assisted with experiments. LLV: performed and assisted with experiments. IA: performed and assisted with experiments. FA: performed and assisted with experiments. VW: performed and assisted with experiments. IG: performed and assisted with experiments. TO‐B: performed and assisted with experiments. ZY: provided assistance with experimental design and implementation. MM: performed experiments. MB: supervised research and contributed to final manuscript. IJ: performed data analysis and interactome assembly. TG: supervised and assisted with experimental design of organoid research. CEB: supervised research and assisted with experimental design. MDA: supervised research and contributed to final manuscript. IS: supervised research and contributed to final manuscript.

## Supporting information



Appendix

Table EV1

Table EV2

Table EV3

Dataset EV1

Dataset EV2

Dataset EV3

Dataset EV4

Dataset EV5

Dataset EV6

Dataset EV7

Dataset EV8

## Data Availability

The data produced in this study are available in the following databases: MaMTH‐HTS Interaction Data: IID Integrated Interaction Database (http://ophid.utoronto.ca/iid). Part of the IMEx Consortium, (Orchard *et al*, 2012; Porras *et al*, 2020). Deposited as MaMTH‐HTS‐2021, identifiable with PMID.

## References

[msb202110629-bib-0001] Aboualizadeh F , Yao Z , Guan J , Drecun L , Pathmanathan S , Snider J , Umapathy G , Kotlyar M , Jurisica I , Palmer R *et al* (2021) Mapping the phospho‐dependent ALK interactome to identify novel components in ALK signaling. J Mol Biol 433: 167294 34606829 10.1016/j.jmb.2021.167283

[msb202110629-bib-0002] Ahmadi S , Bozoky Z , Di Paola M , Xia S , Li C , Wong AP , Wellhauser L , Molinski SV , Ip W , Ouyang H *et al* (2017) Phenotypic profiling of CFTR modulators in patient‐derived respiratory epithelia. NPJ Genomic Med 2: 12 10.1038/s41525-017-0015-6 PMC5481189 28649446

[msb202110629-bib-0003] Almaça J , Faria D , Sousa M , Uliyakina I , Conrad C , Sirianant L , Clarke L , Martins J , Santos M , Heriché J‐K *et al* (2013) High‐content siRNA screen reveals global ENaC regulators and potential cystic fibrosis therapy targets. Cell 154: 1390 24034256 10.1016/j.cell.2013.08.045

[msb202110629-bib-0004] Amaral MD , de Boeck K , Amaral M , Davies JC , de Boeck K , Drevinek P , Elborn S , Kerem E , Lee T (2019) Theranostics by testing CFTR modulators in patient‐derived materials: the current status and a proposal for subjects with rare CFTR mutations. J Cyst Fibros 18: 685–692 31326274 10.1016/j.jcf.2019.06.010

[msb202110629-bib-0005] Amaral MD , Quaresma MC , Pankonien I (2020) What role does cftr play in development, differentiation, regeneration and cancer? Int J Mol Sci 21: 3133 10.3390/ijms21093133 PMC7246864 32365523

[msb202110629-bib-0006] Bear CE , Li C , Kartner N , Bridges RJ , Jensen TJ , Ramjeesingh M , Riordan JR (1992) Purification and functional reconstitution of the cystic fibrosis transmembrane conductance regulator (CFTR). Cell 68: 809–818 1371239 10.1016/0092-8674(92)90155-6

[msb202110629-bib-0007] Benjamini Y , Hochberg Y (1995) Controlling the false discovery rate: a practical and powerful approach to multiple testing. J R Stat Soc Ser B 57: 289–300

[msb202110629-bib-0008] Bézie S , Picarda E , Tesson L , Renaudin K , Durand J , Ménoret S , Mérieau E , Chiffoleau E , Guillonneau C , Caron L *et al* (2015) Fibrinogen‐like protein 2/fibroleukin induces long‐term allograft survival in a rat model through regulatory B cells. PLoS One 10: e0119686 25763980 10.1371/journal.pone.0119686 PMC4357433

[msb202110629-bib-0009] Botelho HM , Uliyakina I , Awatade NT , Proença MC , Tischer C , Sirianant L , Kunzelmann K , Pepperkok R , Amaral MD (2015) Protein traffic disorders: an effective high‐throughput fluorescence microscopy pipeline for drug discovery. Sci Rep 5: 9038 25762484 10.1038/srep09038 PMC4356983

[msb202110629-bib-0010] Boucher RC (2007) Cystic fibrosis: a disease of vulnerability to airway surface dehydration. Trends Mol Med 13: 231–240 17524805 10.1016/j.molmed.2007.05.001

[msb202110629-bib-0011] Breitling R , Armengaud P , Amtmann A , Herzyk P (2004) Rank products: a simple, yet powerful, new method to detect differentially regulated genes in replicated microarray experiments. FEBS Lett 573: 83–92 15327980 10.1016/j.febslet.2004.07.055

[msb202110629-bib-0012] Brown KR , Otasek D , Ali M , McGuffin MJ , Xie W , Devani B , van Toch IL , Jurisica I (2009) NAViGaTOR: network analysis, visualization and graphing Toronto. Bioinformatics 25: 3327–3329 19837718 10.1093/bioinformatics/btp595 PMC2788933

[msb202110629-bib-0013] Caballero I , Ringot‐Destrez B , Si‐Tahar M , Barbry P , Guillon A , Lantier I , Berri M , Chevaleyre C , Fleurot I , Barc C *et al* (2021) Evidence of early increased sialylation of airway mucins and defective mucociliary clearance in CFTR‐deficient piglets. J Cyst Fibros 20: 173–182 32978064 10.1016/j.jcf.2020.09.009

[msb202110629-bib-0014] Cao H , Ouyang H , Grasemann H , Bartlett C , Du K , Duan R , Shi F , Estrada M , Seigel KE , Coates AL *et al* (2018) Transducing airway basal cells with a helper‐dependent adenoviral vector for lung gene therapy. Hum Gene Ther 29: 643–652 29320887 10.1089/hum.2017.201

[msb202110629-bib-0015] Chan CWY , Kay LS , Khadaroo RG , Chan MWC , Lakatoo S , Young KJ , Zhang LI , Gorczynski RM , Cattral M , Rotstein O *et al* (2003) Soluble fibrinogen‐like protein 2/fibroleukin exhibits immunosuppressive properties: suppressing T cell proliferation and inhibiting maturation of bone marrow‐derived dendritic cells. J Immunol 170: 4036–4044 12682232 10.4049/jimmunol.170.8.4036

[msb202110629-bib-0016] Cheng SH , Gregory RJ , Marshall J , Paul S , Souza DW , White GA , O’Riordan CR , Smith AE (1990) Defective intracellular transport and processing of CFTR is the molecular basis of most cystic fibrosis. Cell 63: 827–834 1699669 10.1016/0092-8674(90)90148-8

[msb202110629-bib-0017] Cutting GR (2014) Cystic fibrosis genetics: from molecular understanding to clinical application. Nat Rev Genet 16: 45–56 25404111 10.1038/nrg3849 PMC4364438

[msb202110629-bib-0018] Cystic Fibrosis Foundation (2020) Cystic Fibrosis Foundation Patient Registry 2019 Annual Data Report, Bethesda Maryland

[msb202110629-bib-0019] Dekkers JF , Berkers G , Kruisselbrink E , Vonk A , de Jonge HR , Janssens HM , Bronsveld I , van de Graaf EA , Nieuwenhuis EES , Houwen RHJ *et al* (2016) Characterizing responses to CFTR‐modulating drugs using rectal organoids derived from subjects with cystic fibrosis. Sci Transl Med 8: 344ra84 10.1126/scitranslmed.aad8278 27334259

[msb202110629-bib-0020] Dekkers JF , Wiegerinck CL , de Jonge HR , Bronsveld I , Janssens HM , de Winter‐de Groot KM , Brandsma AM , de Jong NWM , Bijvelds MJC , Scholte BJ *et al* (2013) A functional CFTR assay using primary cystic fibrosis intestinal organoids. Nat Med 19: 939–945 23727931 10.1038/nm.3201

[msb202110629-bib-0021] del‐Toro N , Duesbury M , Koch M , Perfetto L , Shrivastava A , Ochoa D , Wagih O , Piñero J , Kotlyar M , Pastrello C *et al* (2019) Capturing variation impact on molecular interactions in the IMEx Consortium mutations data set. Nat Commun 10: 10 30602777 10.1038/s41467-018-07709-6 PMC6315030

[msb202110629-bib-0022] Denning GM , Anderson MP , Amara JF , Marshall J , Smith AE , Welsh MJ (1992) Processing of mutant cystic fibrosis transmembrane conductance regulator is temperature‐sensitive. Nature 358: 761–764 1380673 10.1038/358761a0

[msb202110629-bib-0023] Du K , Lukacs GL (2009) Cooperative assembly and misfolding of CFTR domains *in vivo* . Mol Biol Cell 20: 1903–1915 19176754 10.1091/mbc.E08-09-0950 PMC2663924

[msb202110629-bib-0024] Eckford P , Ramjeesingh M , Molinski S , Pasyk S , Dekkers JF , Li C , Ahmadi S , Ip W , Chung T , Du K *et al* (2014) VX‐809 and related corrector compounds exhibit secondary activity stabilizing active F508del‐CFTR after its partial rescue to the cell surface. Chem Biol 21: 666–678 24726831 10.1016/j.chembiol.2014.02.021

[msb202110629-bib-0025] Ernst WL , Shome K , Wu CC , Gong X , Frizzell RA , Aridor M (2016) VAMP‐associated proteins (VAP) as receptors that couple cystic fibrosis transmembrane conductance regulator (CFTR) proteostasis with lipid homeostasis. J Biol Chem 291: 5206–5220 26740627 10.1074/jbc.M115.692749 PMC4777854

[msb202110629-bib-0026] Fajac I , Wainwright CE (2017) New treatments targeting the basic defects in cystic fibrosis. Press Medicale 46: e165–e175 10.1016/j.lpm.2017.01.024 28554723

[msb202110629-bib-0027] Foerster K , Helmy A , Zhu YI , Khattar R , Adeyi OA , Wong KM , Shalev I , Clark DA , Wong P‐Y , Heathcote EJ *et al* (2010) The novel immunoregulatory molecule FGL2: a potential biomarker for severity of chronic hepatitis C virus infection. J Hepatol 53: 608–615 20615566 10.1016/j.jhep.2010.04.020

[msb202110629-bib-0028] Gabriel SE , Brigman KN , Koller BH , Boucher RC , Stutts MJ (1994) Cystic fibrosis heterozygote resistance to cholera toxin in the cystic fibrosis mouse model. Science 266: 107–109 7524148 10.1126/science.7524148

[msb202110629-bib-0029] Geisler M , Hegedűs T (2020) A twist in the ABC: regulation of ABC transporter trafficking and transport by FK506‐binding proteins. FEBS Lett 594: 3986–4000 33125703 10.1002/1873-3468.13983

[msb202110629-bib-0030] Gibson DG , Young L , Chuang R‐Y , Venter JC , Hutchison CA , Smith HO (2009) Enzymatic assembly of DNA molecules up to several hundred kilobases. Nat Methods 6: 343–345 19363495 10.1038/nmeth.1318

[msb202110629-bib-0031] Grozavu I , Stuart S , Lyakisheva A , Yao Z , Pathmanathan S , Ohh M , Stagljar I (2022) D154Q mutation does not alter KRAS dimerization. J Mol Biol 434: 167392 34896362 10.1016/j.jmb.2021.167392

[msb202110629-bib-0032] Guan D , Lazar M (2019) Shining light on dark matter in the genome. Proc Natl Acad Sci USA 116: 24919–24921 31740615 10.1073/pnas.1918894116 PMC6911184

[msb202110629-bib-0033] Guilbault C , Novak JP , Martin P , Boghdady ML , Saeed Z , Guiot MC , Hudson TJ , Radzioch D (2006) Distinct pattern of lung gene expression in the Cftr‐KO mice developing spontaneous lung disease compared with their littermate controls. Physiol Genomics 25: 179–193 16418321 10.1152/physiolgenomics.00206.2005

[msb202110629-bib-0034] Hagemeijer MC , Vonk AM , Awatade NT , Silva IAL , Tischer C , Hilsenstein V , Beekman JM , Amaral MD , Botelho HM (2020) An open‐source high‐content analysis workflow for CFTR function measurements using the forskolin‐induced swelling assay. Bioinformatics 36: 5686–5694 10.1093/bioinformatics/btaa1073 33367496

[msb202110629-bib-0035] Häsler SLA , Vallis Y , Pasche M , McMahon HT (2020) GRAF2, WDR44, and MICAL1 mediate Rab8/10/11–dependent export of E‐cadherin, MMP14, and CFTR ΔF508. J Cell Biol 219: e201811014 32344433 10.1083/jcb.201811014 PMC7199855

[msb202110629-bib-0036] Hotta A , Cheung AYL , Farra N , Garcha K , Chang WY , Pasceri P , Stanford WL , Ellis J (2009) EOS lentiviral vector selection system for human induced pluripotent stem cells. Nat Protoc 4: 1828–1844 20010937 10.1038/nprot.2009.201

[msb202110629-bib-0037] Huntley RP , Sawford T , Mutowo‐Meullenet P , Shypitsyna A , Bonilla C , Martin MJ , O’Donovan C (2015) The GOA database: gene Ontology annotation updates for 2015. Nucleic Acids Res 43: D1057–D1063 25378336 10.1093/nar/gku1113 PMC4383930

[msb202110629-bib-0038] Hutt DM , Herman D , Rodrigues APC , Noel S , Pilewski JM , Matteson J , Hoch B , Kellner W , Kelly JW , Schmidt A *et al* (2010) Reduced histone deacetylase 7 activity restores function to misfolded CFTR in cystic fibrosis. Nat Chem Biol 6: 25–33 19966789 10.1038/nchembio.275 PMC2901172

[msb202110629-bib-0039] Johnsson N , Varshavsky A (1994) Split ubiquitin as a sensor of protein interactions *in vivo* . Proc Natl Acad Sci USA 91: 10340–10344 7937952 10.1073/pnas.91.22.10340 PMC45015

[msb202110629-bib-0040] Knudson AG , Wayne L , Hallett WY (1967) On the selective advantage of cystic fibrosis heterozygotes. Am J Hum Genet 19: 388–392 6026931 PMC1706220

[msb202110629-bib-0041] Kotlyar M , Pastrello C , Malik Z , Jurisica I (2019) IID 2018 update: context‐specific physical protein–protein interactions in human, model organisms and domesticated species. Nucleic Acids Res 47: D581–D589 30407591 10.1093/nar/gky1037 PMC6323934

[msb202110629-bib-0042] Kotlyar M , Pastrello C , Ahmed Z , Chee J , Varyova Z , Jurisica I (2021) IID 2021: towards context‐specific protein interaction analyses by increased coverage, enhanced annotation and enrichment analysis. Nucleic Acids Res 1: 13–14 10.1093/nar/gkab1034 PMC8728267 34755877

[msb202110629-bib-0043] Langmead B , Salzberg SL (2012) Fast gapped‐read alignment with Bowtie 2. Nat Methods 9: 357–359 22388286 10.1038/nmeth.1923 PMC3322381

[msb202110629-bib-0044] Lérias JR , Pinto MC , Botelho HM , Awatade NT , Quaresma MC , Silva IAL , Wanitchakool P , Schreiber R , Pepperkok R , Kunzelmann K *et al* (2018) A novel microscopy‐based assay identifies extended synaptotagmin‐1 (ESYT1) as a positive regulator of anoctamin 1 traffic. Biochim Biophys Acta ‐ Mol Cell Res 1865: 421–431 29154949 10.1016/j.bbamcr.2017.11.009

[msb202110629-bib-0045] Levy GA , Liu M , Ding J , Yuwaraj S , Leibowitz J , Marsden PA , Ning Q , Kovalinka AA , Phillips MJ (2000) Molecular and functional analysis of the human prothrombinase gene (HFGL2) and its role in viral hepatitis. Am J Pathol 156: 1217–1225 10751347 10.1016/S0002-9440(10)64992-9 PMC1876871

[msb202110629-bib-0046] Li C , Naren AP (2010) CFTR chloride channel in the apical compartments: spatiotemporal coupling to its interacting partners. Integr Biol 2: 161–177 10.1039/b924455g PMC2989726 20473396

[msb202110629-bib-0047] Li H , Sheppard DN , Hug MJ (2004) Transepithelial electrical measurements with the Ussing chamber. J Cyst Fibros 3: 123–126 15463943 10.1016/j.jcf.2004.05.026

[msb202110629-bib-0048] Lim S , Legere E , Snider J , Stagljar I (2018) Recent progress in CFTR interactome mapping and its importance for cystic fibrosis. Front Pharmacol 8: 997 29403380 10.3389/fphar.2017.00997 PMC5785726

[msb202110629-bib-0049] Liu BQ , Bao ZY , Zhu JY , Liu H (2021) Fibrinogen‐like protein 2 promotes the accumulation of myeloid‐derived suppressor cells in the hepatocellular carcinoma tumor microenvironment. Oncol Lett 21: 1–10 33281958 10.3892/ol.2020.12308 PMC7709556

[msb202110629-bib-0050] Lopes‐Pacheco M (2020) CFTR modulators: the changing face of cystic fibrosis in the era of precision medicine. Front Pharmacol 10: 1662 32153386 10.3389/fphar.2019.01662 PMC7046560

[msb202110629-bib-0051] Lukacs GL , Verkmana S (2012) CFTR: folding, misfolding and correcting the ΔF508 conformational defect. Trends Mol Med 18: 81–91 22138491 10.1016/j.molmed.2011.10.003 PMC3643519

[msb202110629-bib-0052] Manns MP , Buti M , Gane E , Pawlotsky JM , Razavi H , Terrault N , Younossi Z (2017) Hepatitis C virus infection. Nat Rev Dis Prim 3: 17006 28252637 10.1038/nrdp.2017.6

[msb202110629-bib-0053] Marazzi S , Blum S , Hartmann R , Gundersen D , Schreyer M , Argraves S , Von Fliedner V , Pytela R , Rüegg C (1998) Characterization of human fibroleukin, a fibrinogen‐like protein secreted by T lymphocytes. J Immunol 161: 138–147 9647217

[msb202110629-bib-0054] McQuin C , Goodman A , Chernyshev V , Kamentsky L , Cimini BA , Karhohs KW , Doan M , Ding L , Rafelski SM , Thirstrup D *et al* (2018) Cell profiler 3.0: next‐generation image processing for biology. PLoS Biol 16: e2005970 29969450 10.1371/journal.pbio.2005970 PMC6029841

[msb202110629-bib-0055] Mehta G , Macek M , Mehta A (2010) Cystic fibrosis across Europe: EuroCareCF analysis of demographic data from 35 countries. J Cyst Fibros Suppl 2: S5–S21 10.1016/j.jcf.2010.08.002 21041121

[msb202110629-bib-0056] Molinski SV (2016) Biochemical interrogation of rare cystic fibrosis mutations informs strategies for future therapeutic intervention. ProQuest Diss Theses 10137480

[msb202110629-bib-0057] Molinski SV , Ahmadi S , Hung M , Bear CE (2015) Facilitating structure‐function studies of CFTR modulator sites with efficiencies in mutagenesis and functional screening. J Biomol Screen 20: 1204–1217 26385858 10.1177/1087057115605834

[msb202110629-bib-0058] Molinski SV , Ahmadi S , Ip W , Ouyang H , Villella A , Miller JP , Lee P , Kulleperuma K , Du K , Di Paola M *et al* (2017) Orkambi^®^ and amplifier co‐therapy improves function from a rare CFTR mutation in gene‐edited cells and patient tissue. EMBO Mol Med 9: 1224–1243 28667089 10.15252/emmm.201607137 PMC5582412

[msb202110629-bib-0059] Morral N , Bertranpetit J , Estivill X , Nunes V , Casals T , Giménez J , Reis A , Varon‐Mateeva R , Macek M , Kalaydjieva L *et al* (1994) The origin of the major cystic fibrosis mutation (ΔF508) in European populations. Nat Genet 7: 169–175 7920636 10.1038/ng0694-169

[msb202110629-bib-0060] Nakagawa K , Noguchi Y , Uenaka A , Sato S , Okumura H , Tanaka M , Shimono M , Ali Eldib AM , Ono T , Ohara N *et al* (2005) XAGE‐1 expression in non‐small cell lung cancer and antibody response in patients. Clin Cancer Res 11: 5496–5503 16061866 10.1158/1078-0432.CCR-05-0216

[msb202110629-bib-0061] Neumann B , Walter T , Hériché J‐K , Bulkescher J , Erfle H , Conrad C , Rogers P , Poser I , Held M , Liebel U *et al* (2010) Phenotypic profiling of the human genome by time‐lapse microscopy reveals cell division genes. Nature 464: 721–727 20360735 10.1038/nature08869 PMC3108885

[msb202110629-bib-0062] Ning Q , Sun YI , Han M , Zhang LI , Zhu C , Zhang W , Guo H , Li J , Yan W , Gong F *et al* (2005) Role of fibrinogen‐like protein 2 prothrombinase/fibroleukin in experimental and human allograft rejection. J Immunol 174: 7403–7411 15905589 10.4049/jimmunol.174.11.7403

[msb202110629-bib-0063] Okiyoneda T , Barrière H , Bagdány M , Rabeh WM , Du K , Höhfeld J , Young JC , Lukacs GL (2010) Peripheral protein quality control removes unfolded CFTR from the plasma membrane. Science 329: 805–810 20595578 10.1126/science.1191542 PMC5026491

[msb202110629-bib-0064] Orchard S , Kerrien S , Abbani S , Aranda B , Bhate J , Bidwell S , Bridge A , Briganti L , Brinkman FSL , Cesareni G *et al* (2012) Protein interaction data curation: the International Molecular Exchange (IMEx) consortium. Nat Methods 9: 345–350 22453911 10.1038/nmeth.1931 PMC3703241

[msb202110629-bib-0065] Pankow S , Bamberger C , Calzolari D , Martínez‐Bartolomé S , Lavallée‐Adam M , Balch WE , Yates JR (2015) ∆F508 CFTR interactome remodelling promotes rescue of cystic fibrosis. Nature 528: 510–516 26618866 10.1038/nature15729 PMC4826614

[msb202110629-bib-0066] Paumi CM , Chuk M , Chevelev I , Stagljar I , Michaelis S (2008) Negative regulation of the yeast ABC transporter Ycf1p by phosphorylation within its N‐terminal extension. J Biol Chem 283: 27079–27088 18667437 10.1074/jbc.M802569200 PMC2555997

[msb202110629-bib-0067] Petschnigg J , Groisman B , Kotlyar M , Taipale M , Zheng Y , Kurat CF , Sayad A , Sierra JR , Usaj MM , Snider J *et al* (2014) The mammalian‐membrane two‐hybrid assay (MaMTH) for probing membrane‐protein interactions in human cells. Nat Methods 11: 585–592 24658140 10.1038/nmeth.2895

[msb202110629-bib-0068] Petschnigg J , Kotlyar M , Blair L , Jurisica I , Stagljar I , Ketteler R (2017) Systematic identification of oncogenic EGFR interaction partners. J Mol Biol 429: 280–294 27956147 10.1016/j.jmb.2016.12.006 PMC5240790

[msb202110629-bib-0069] Piñero J , Ramírez‐Anguita JM , Saüch‐Pitarch J , Ronzano F , Centeno E , Sanz F , Furlong LI (2020) The DisGeNET knowledge platform for disease genomics: 2019 update. Nucleic Acids Res 48: D845–D855 31680165 10.1093/nar/gkz1021 PMC7145631

[msb202110629-bib-0070] Pitonzo D , Yang Z , Matsumura Y , Johnson AE , Skach WR (2009) Sequence‐specific retention and regulated integration of a nascent membrane protein by the endoplasmic reticulum Sec61 translocon. Mol Biol Cell 20: 685–698 19019984 10.1091/mbc.E08-09-0902 PMC2626564

[msb202110629-bib-0071] Porras P , Barrera E , Bridge A , del‐Toro N , Cesareni G , Duesbury M , Hermjakob H , Iannuccelli M , Jurisica I , Kotlyar M *et al* (2020) Towards a unified open access dataset of molecular interactions. Nat Commun 11: 6144 33262342 10.1038/s41467-020-19942-z PMC7708836

[msb202110629-bib-0072] Rahmati S , Abovsky M , Pastrello C , Kotlyar M , Lu R , Rahman P , Chandran V , Jurisica I (2019) PathDIP 4: an extended pathway annotations and enrichment resource for human, model organisms, and domesticated species. Nucleic Acids Res 48: D479–D488 10.1093/nar/gkz989 PMC7145646 31733064

[msb202110629-bib-0073] Ran FA , Hsu PD , Wright J , Agarwala V , Scott DA , Zhang F (2013) Genome engineering using the CRISPR‐Cas9 system. Nat Protoc 8: 2281–2308 24157548 10.1038/nprot.2013.143 PMC3969860

[msb202110629-bib-0074] de Ridder GG , Lundblad RL , Pizzo SV (2016) Actions of thrombin in the interstitium. J Thromb Haemost 14: 40–47 26564405 10.1111/jth.13191

[msb202110629-bib-0075] Riordan JR (2005) Assembly of functional CFTR chloride channels. Annu Rev Physiol 67: 701–718 15709975 10.1146/annurev.physiol.67.032003.154107

[msb202110629-bib-0076] Riordan JR (2008) CFTR function and prospects for therapy. Annu Rev Biochem 77: 701–726 18304008 10.1146/annurev.biochem.75.103004.142532

[msb202110629-bib-0077] Riordan JR , Rommens JM , Kerem B‐S , Alon N , Rozmahel R , Grzelczak Z , Zielenski J , Lok SI , Plavsic N , Chou J‐L *et al* (1989) Identification of the cystic fibrosis gene: cloning and characterization of complementary DNA. Science 245: 1066–1073 2475911 10.1126/science.2475911

[msb202110629-bib-0078] Santos JD , Pinto FR , Ferreira JF , Amaral MD , Zaccolo M , Farinha CM (2020) Cytoskeleton regulators CAPZA2 and INF2 associate with CFTR to control its plasma membrane levels under EPAC1 activation. Biochem J 477: 2561–2580 32573649 10.1042/BCJ20200287

[msb202110629-bib-0079] Saraon P , Grozavu I , Lim S , Snider J , Yao Z , Stagljar I (2017) Detecting membrane protein‐protein interactions using the mammalian membrane two‐hybrid (MaMTH) assay. Curr Protoc Chem Biol 9: 38–54 28253435 10.1002/cpch.15

[msb202110629-bib-0080] Saraon P , Snider J , Kalaidzidis Y , Wybenga‐Groot LE , Weiss K , Rai A , Radulovich N , Drecun L , Vučković N , Vučetić A *et al* (2020) A drug discovery platform to identify compounds that inhibit EGFR triple mutants. Nat Chem Biol 16: 577–586 32094923 10.1038/s41589-020-0484-2 PMC8123931

[msb202110629-bib-0081] Saraon P , Snider J , Schormann W , Rai A , Radulovich N , Sánchez‐Osuna M , Coulombe‐Huntington J , Huard C , Mohammed M , Lima‐Fernandes E *et al* (2021) Chemical genetics screen identifies COPB2 tool compounds that alters ER stress response and induces RTK dysregulation in lung cancer cells. J Mol Biol 433: 167294 34662547 10.1016/j.jmb.2021.167294

[msb202110629-bib-0082] Scheper W , Thaminy S , Kais S , Stagljar I , Römisch K (2003) Coordination of N‐glycosylation and protein translocation across the endoplasmic reticulum membrane by Sss1 protein. J Biol Chem 278: 37998–38003 12860997 10.1074/jbc.M300176200

[msb202110629-bib-0083] Schroeder SA , Gaughan DM , Swift M (1995) Protection against bronchial asthma by CFTR Δf508 mutation: a heterozygote advantage in cystic fibrosis. Nat Med 1: 703–705 7585155 10.1038/nm0795-703

[msb202110629-bib-0084] Simpson JC , Joggerst B , Laketa V , Verissimo F , Cetin C , Erfle H , Bexiga MG , Singan VR , Hériché J‐K , Neumann B *et al* (2012) Genome‐wide RNAi screening identifies human proteins with a regulatory function in the early secretory pathway. Nat Cell Biol 14: 764–774 22660414 10.1038/ncb2510

[msb202110629-bib-0085] Sokolina K , Kittanakom S , Snider J , Kotlyar M , Maurice P , Gandía J , Benleulmi‐Chaachoua A , Tadagaki K , Oishi A , Wong V *et al* (2017) Systematic protein‐protein interaction mapping for clinically relevant human GPCRs. Mol Syst Biol 13: 918 28298427 10.15252/msb.20167430 PMC5371730

[msb202110629-bib-0086] Stagljar I , Korostensky C , Johnsson N , te Heesen S (1998) A genetic system based on split‐ubiquitin for the analysis of interactions between membrane proteins *in vivo* . Proc Natl Acad Sci USA 95: 5187–5192 9560251 10.1073/pnas.95.9.5187 PMC20236

[msb202110629-bib-0087] Van Lidth de Jeude JF , Vermeulen JLM , Montenegro‐Miranda PS , Van den Brink GR , Heijmans J (2015) A protocol for lentiviral transduction and downstream analysis of intestinal organoids. J vis Exp 98: 52531 10.3791/52531 PMC4541580 25938265

[msb202110629-bib-0088] Wang X , Venable J , LaPointe P , Hutt DM , Koulov AV , Coppinger J , Gurkan C , Kellner W , Matteson J , Plutner H *et al* (2006) Hsp90 cochaperone Aha1 downregulation rescues misfolding of CFTR in cystic fibrosis. Cell 127: 803–815 17110338 10.1016/j.cell.2006.09.043

[msb202110629-bib-0089] Ward CL , Kopito RR (1994) Intracellular turnover of cystic fibrosis transmembrane conductance regulator. Inefficient processing and rapid degradation of wild‐type and mutant proteins. J Biol Chem 269: 25710–25718 7523390

[msb202110629-bib-0090] Wu S , Li M , Xu F , Li G‐Q , Han BO , He X‐D , Li S‐J , He Q‐H , Lai X‐Y , Zhou S *et al* (2020) Fibrinogen‐like protein 2 deficiency aggravates renal fibrosis by facilitating macrophage polarization. Biomed Pharmacother 130: 110468 32795921 10.1016/j.biopha.2020.110468

[msb202110629-bib-0091] Yang X , Boehm JS , Yang X , Salehi‐Ashtiani K , Hao T , Shen Y , Lubonja R , Thomas SR , Alkan O , Bhimdi T *et al* (2011) A public genome‐scale lentiviral expression library of human ORFs. Nat Methods 8: 659–661 21706014 10.1038/nmeth.1638 PMC3234135

[msb202110629-bib-0092] Yao Z , Darowski K , St‐Denis N , Wong V , Offensperger F , Villedieu A , Amin S , Malty R , Aoki H , Guo H *et al* (2017) A global analysis of the receptor tyrosine kinase‐protein phosphatase interactome. Mol Cell 65: 347–360 28065597 10.1016/j.molcel.2016.12.004 PMC5663465

[msb202110629-bib-0093] Zendman AJW , van Kraats AA , Weidle UH , Ruiter DJ , van Muijen GNP (2002) TheXAGE family of cancer/testis‐associated genes: Alignment and expression profile in normal tissues, melanoma lesions and Ewing’s sarcoma. Int J Cancer 99: 361–369 11992404 10.1002/ijc.10371

[msb202110629-bib-0094] Zielenski J , Tsui LC (1995) Cystic fibrosis: genotypic and phenotypic variations. Annu Rev Genet 29: 777–807 8825494 10.1146/annurev.ge.29.120195.004021

[msb202110629-bib-0095] Zolin A, Orenti A, Naerhlich L, Jung A, van Rense J (2020) ECFSPR Annual Report 2018

[msb202110629-bib-0096] Zomer‐van Ommen D , de Poel E , Kruisselbrink E , Oppelaar H , Vonk A , Janssens H , van der Ent C , Hagemeijer M , Beekman J (2018) Comparison of ex vivo and *in vitro* intestinal cystic fibrosis models to measure CFTR‐dependent ion channel activity. J Cyst Fibros 17: 316–324 29544685 10.1016/j.jcf.2018.02.007

