# Supplementary material for: CFTR interactome mapping using the mammalian membrane two‐hybrid high‐throughput screening system
Source: Mol Syst Biol. 2022 Feb 14;18(2):e10629. doi: 10.15252/msb.202110629 (PMC8842165; doi:10.15252/msb.202110629)
Supplement: Supplementary file 1 — Appendix [file MSB-18-e10629-s008.pdf]

Table of Contents

Appendix Figure S1– Overview of the MaMTH system. Integral membrane 'bait', fused to the C-terminus of ubiquitin (Cub) and an artificial transcription factor (TF), is expressed alongside cytosolic or membrane-bound 'prey' fused to the N-terminus of ubiquitin (Nub) in cells carrying a reporter expression system. Interaction of bait and prey leads to ubiquitin reconstitution, proteolytic cleavage by deubiquitinating enzymes (DUBs) and subsequent release of the TF, which then enters the nucleus of the cell and activates reporter (luciferase in the traditional MaMTH system or eGFP in MaMTH-HTS).

Appendix Figure S2 – MaMTH bait and prey cassettes/plasmids. A) Schematic overview of MaMTH bait cassette and other major plasmid features. B) Schematic overview of MaMTH prey cassette and other major plasmid features.

Appendix Figure S3 – Live HEK293 cell fluorescence microscopy of transiently expressed wt- and F508-del CFTR proteins fused to MaMTH bait tag (C-terminus of ubiquitin and artificial transcription factor) and GFP showing that expected localization is unaffected. Plasma membrane (PM Marker) was stained using CellMask (Invitrogen). Endoplasmic reticulum (ER marker) was stained using ER-Tracker (Invitrogen).

Appendix Figure S4 – Graphical summary of shared and differential interaction numbers across technical replicates and WT vs. F508del. A) Comparison of wt-CFTR interactions across technical replicates 1 and 2. B) Comparison of F508del-CFTR interactions across technical replicates 1 and 2. C) Comparison of interactions between wt-CFTR and F508del-CFTR.

Appendix Figure S5 – Annotated MaMTH-HTS interactome for wt- and F508del- CFTR overlapping with known experimental and predicted interactions from IID v 2021-05. Edge color signifies source of interactions (wt, del or IID), and thickness shows type of overlap with known interactions, according to legend. Node color represents Gene Ontology Biological process. Node label colouring highlights interactors functionally validated in this work.

Appendix Figure S6 – Summary of MaMTH luciferase analysis of selected interactors identified in MaMTH-HTS. A) Proportion of wt-CFTR interactors identified in MaMTH-HTS producing significant signal in traditional MaMTH. B) Proportion of F508del-CFTR interactors identified in MaMTH-HTS producing significant signal in traditional MaMTH. C) Proportion of wt-CFTR interactors identified in MaMTH-HTS that interact with F508del-CFTR in traditional MaMTH. D) Proportion of F508del-CFTR interactors identified in MaMTH-HTS that interact with wt-CFTR in traditional MaMTH.

Appendix Figure S7 – FLIPR membrane potential traces for interactors whose transient expression in HEK293 cells affects CFTR channel activity. A) Interactors affecting F508del-CFTR channel activity. B) Interactors affecting wt-CFTR channel

activity. Three select representative traces for interactors whose expression did not affect channel activity are also shown for each.

Appendix Figure S8 – Hit siRNAs in the high-content CFTR trafficking assay. Representative images of CFBE cells expressing the wt- or F508del- variants of the mCherry-Flag-CFTR traffic reporter. Hoechst stains nuclei, mCherry labels all CFTR molecules and immunostained Flag in unpermeabilized cells labels CFTR molecules located at the PM. Cells treated with the non-targeting Neg1 siRNA or F508del-CFTR correctors (VX-809, VX-661) were taken as negative or positive controls, respectively. Significant loss of CFTR expression in cells treated with a CFTR-targeting siRNA indicates high transfection efficiency. The remaining conditions represent cells treated with siRNAs which rescue F508del-CFTR PM localization (Z-score > 1), as seen by increased fluorescence intensity in the Flag channel versus the negative control. One of the siRNAs targeting FGL2, which did not meet the Z-score hit threshold in the traffic screen, is also shown. Corresponding channels are shown in the same lookup table, except for wt-CFTR expressing cells, where contrast required adjustment due to the overall larger fluorescence intensity. Scale bar: 50 μm.

Appendix Figure S9 – Validation of selected interactions via co-immunoprecipitation (Co-IP) in HEK293T cells. A) Co-IP experiments showing 17 successfully validated interactions (out of 30 tested). Co-IPs were performed using anti-FLAG antibody directed against overexpressed FLAG-tagged protein corresponding to identified interactors, followed by Western blotting using anti-FLAG antibody (top panels) and anti-V5 antibody directed against the V5-tagged wildtype CFTR (lower panels). Red asterisks indicate bands of the size corresponding to the FLAG-tagged interactor protein being tested. Images are representative of n=2 biological replicates. B) Co-IPs performed to confirm the FGL2/CFTR interaction. Pull-downs were performed using anti-V5 antibody, followed by Western blotting with anti-V5 and anti-FLAG antibody (top panel) or using anti-FLAG antibody, followed by Western blotting with anti-FLAG and anti-V5 antibody (bottom panel). C) Control co-IPs for all tested samples, performed using unrelated anti-GAPDH antibody, showing that over-expressed FLAG-tagged interactors do not non-specifically pull-down unrelated protein.

Appendix Figure S10 – Overview of the validation strategy used in this work. Created with BioRender.com.

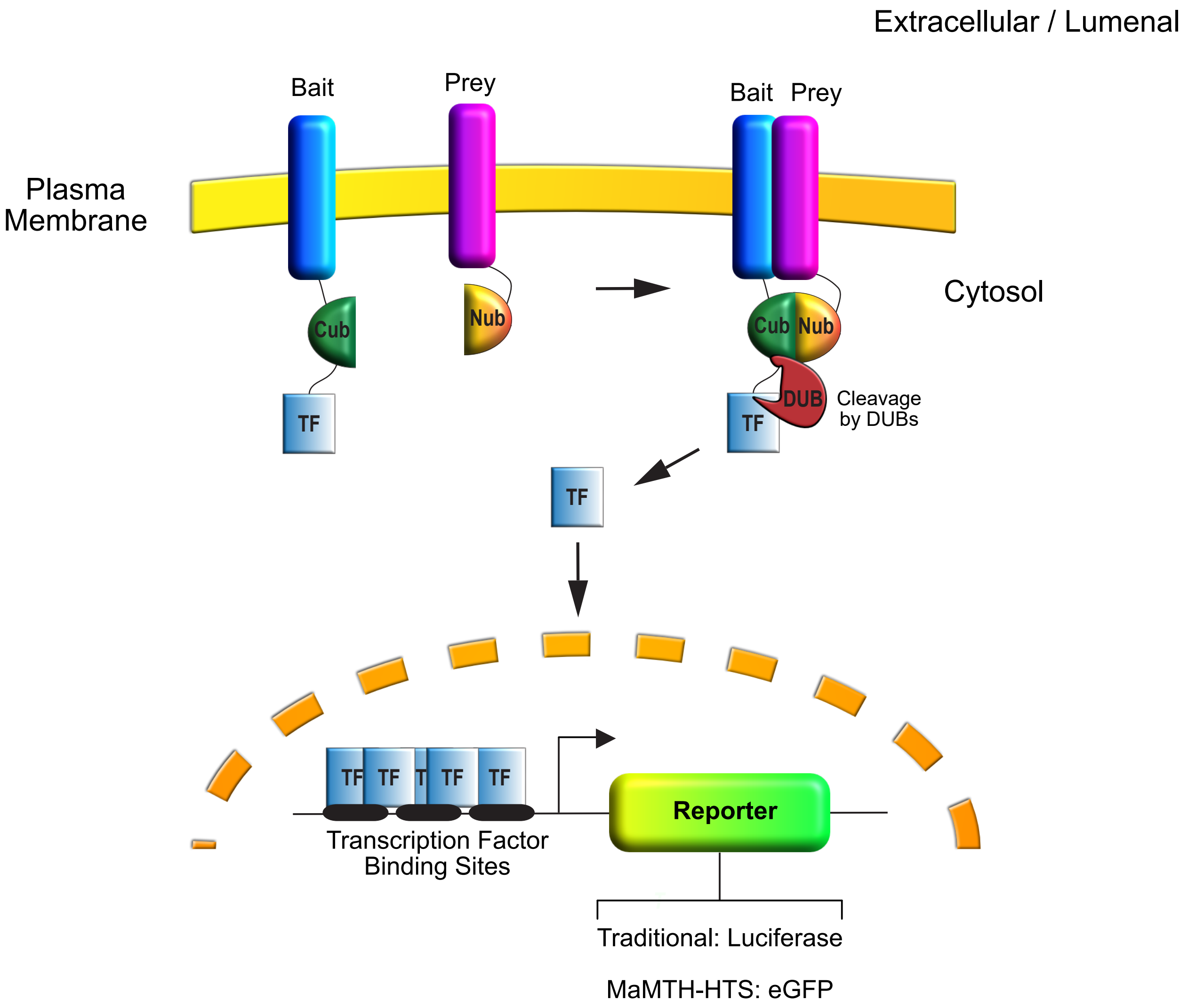

Appendix Figure S1– Overview of the MaMTH system. Integral membrane 'bait', fused to the C-terminus of ubiquitin (Cub) and an artificial transcription factor (TF), is expressed alongside cytosolic or membrane-bound 'prey' fused to the N-terminus of ubiquitin (Nub) in cells carrying a reporter expression system. Interaction of bait and prey leads to ubiquitin reconstitution, proteolytic cleavage by deubiquitinating enzymes (DUBs) and subsequent release of the TF, which then enters the nucleus of the cell and activates reporter (luciferase in the traditional MaMTH system or eGFP in MaMTH-HTS).

A)

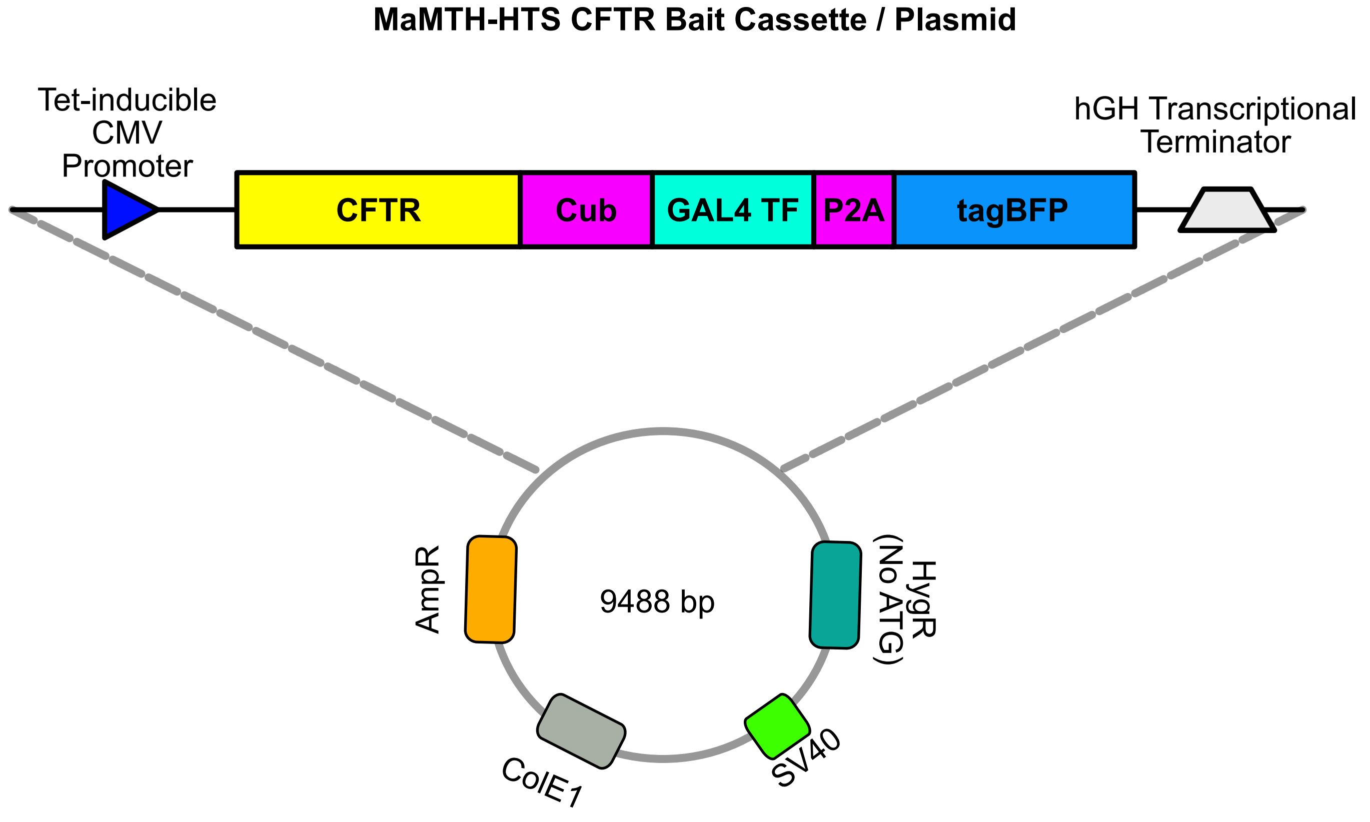

B)

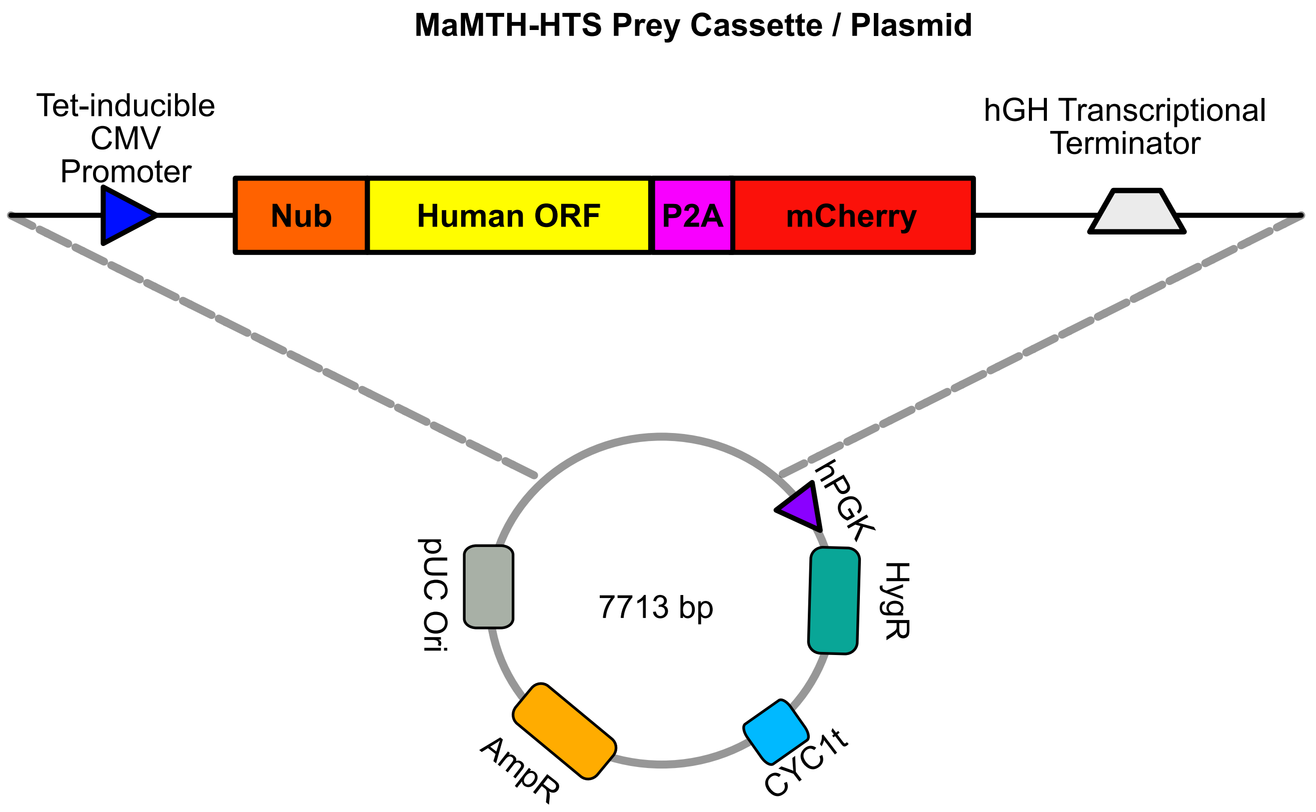

Appendix Figure S2 – MaMTH bait and prey cassettes/plasmids. A) Schematic overview of MaMTH bait cassette and other major plasmid features. B) Schematic overview of MaMTH prey cassette and other major plasmid features.

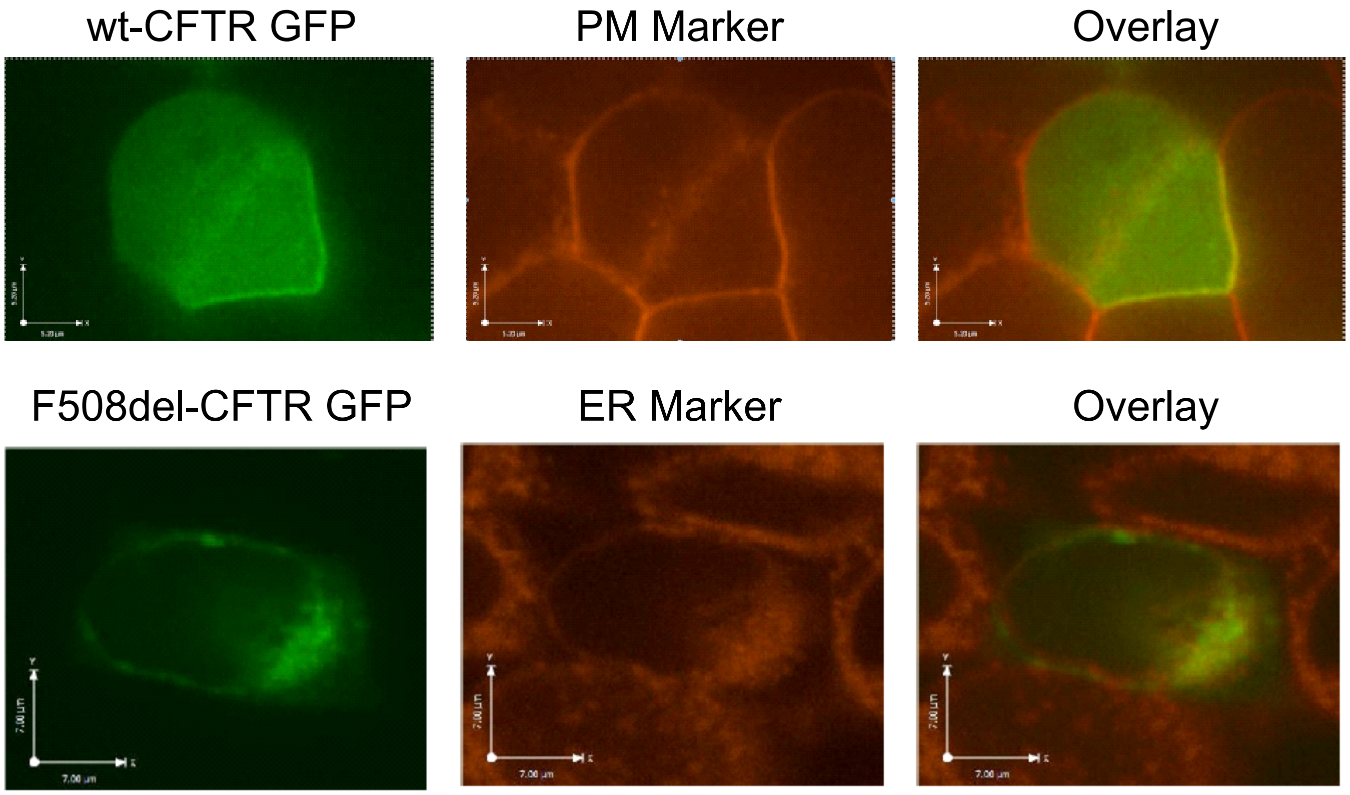

Appendix Figure S3 – Live HEK293 cell fluorescence microscopy of transiently expressed wt- and F508-del CFTR proteins fused to MaMTH bait tag (C-terminus of ubiquitin and artificial transcription factor) and GFP showing that expected localization is unaffected. Plasma membrane (PM Marker) was stained using CellMask (Invitrogen). Endoplasmic reticulum (ER marker) was stained using ER-Tracker (Invitrogen).

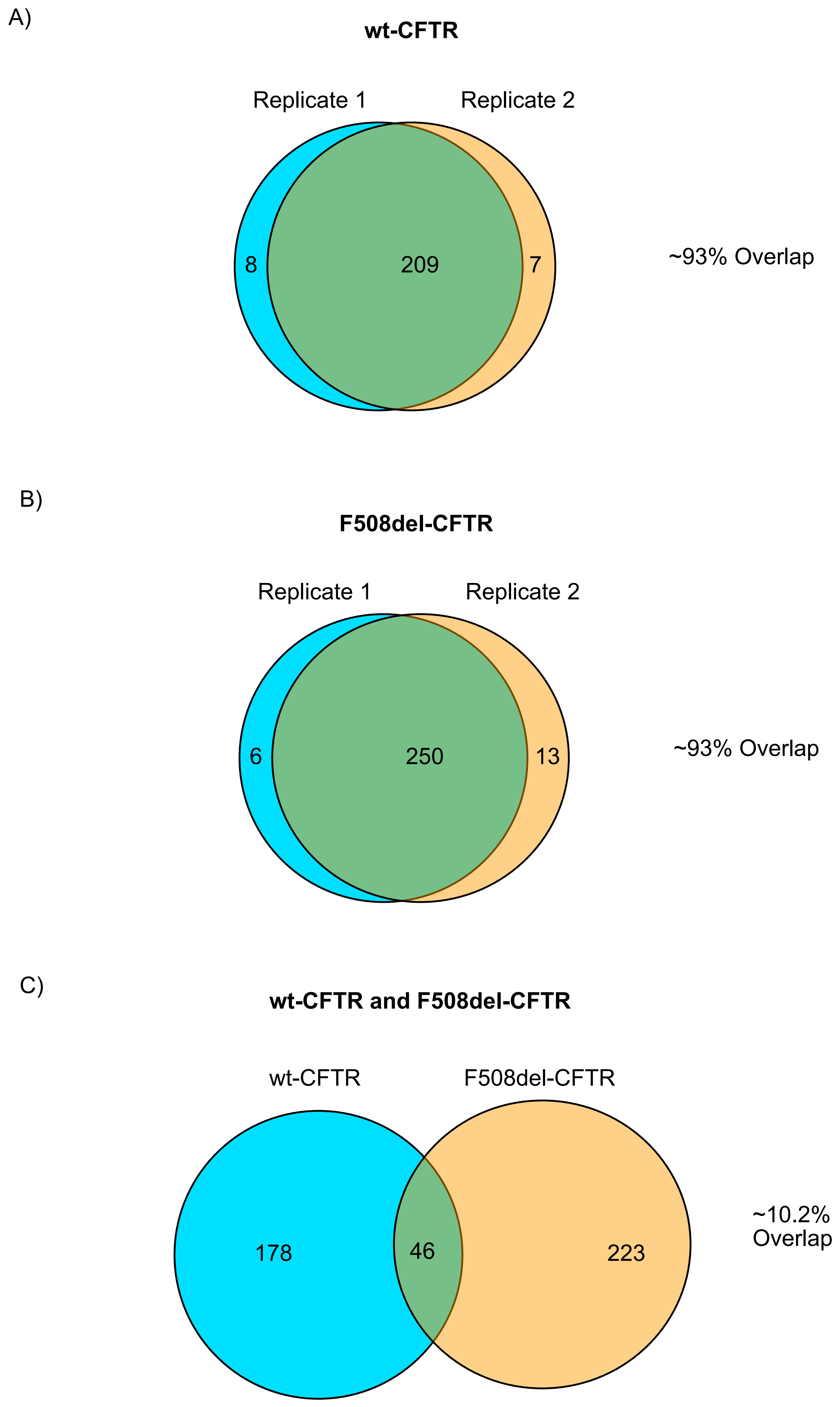

Appendix Figure S4 – Graphical summary of shared and differential interaction numbers across technical replicates and WT vs. F508del. A) Comparison of wt-CFTR interactions across technical replicates 1 and 2. B) Comparison of F508del-CFTR interactions across technical replicates 1 and 2. C) Comparison of interactions between wt-CFTR and F508del-CFTR.

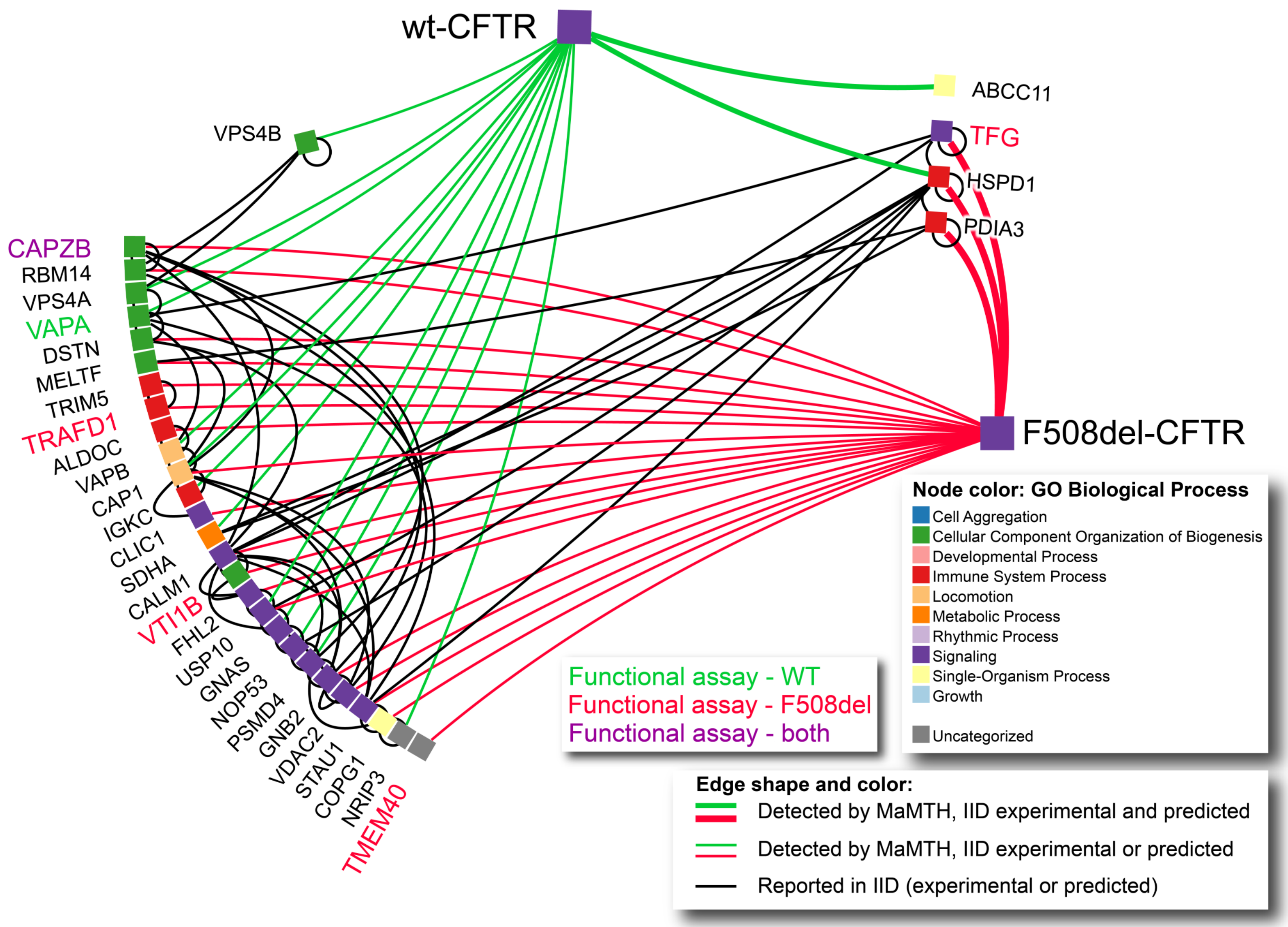

Appendix Figure S5 – Annotated MaMTH-HTS interactome for wt- and F508del- CFTR overlapping with known experimental and predicted interactions from IID v 2021-05. Edge color signifies source of interactions (wt, del or IID), and thickness shows type of overlap with known interactions, according to legend. Node color represents Gene Ontology Biological process. Node label colouring highlights interactors functionally validated in this work.

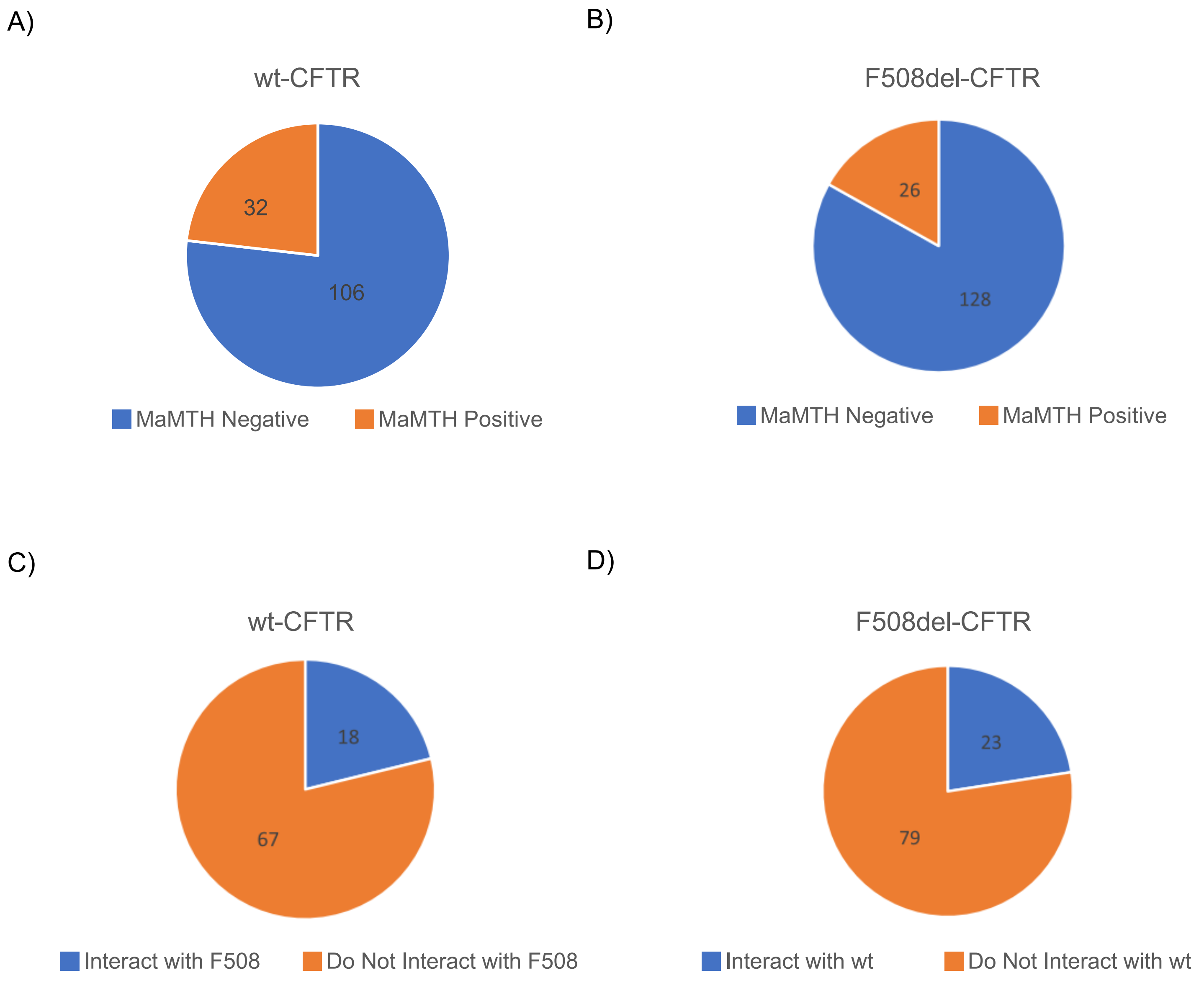

Appendix Figure S6 – Summary of MaMTH luciferase analysis of selected interactors identified in MaMTH-HTS. A) Proportion of wt-CFTR interactors identified in MaMTH-HTS producing significant signal in traditional MaMTH. B) Proportion of F508del-CFTR interactors identified in MaMTH-HTS producing significant signal in traditional MaMTH. C) Proportion of wt-CFTR interactors identified in MaMTH-HTS that interact with F508del-CFTR in traditional MaMTH. D) Proportion of F508del-CFTR interactors identified in MaMTH-HTS that interact with wt-CFTR in traditional MaMTH.

A)

B)

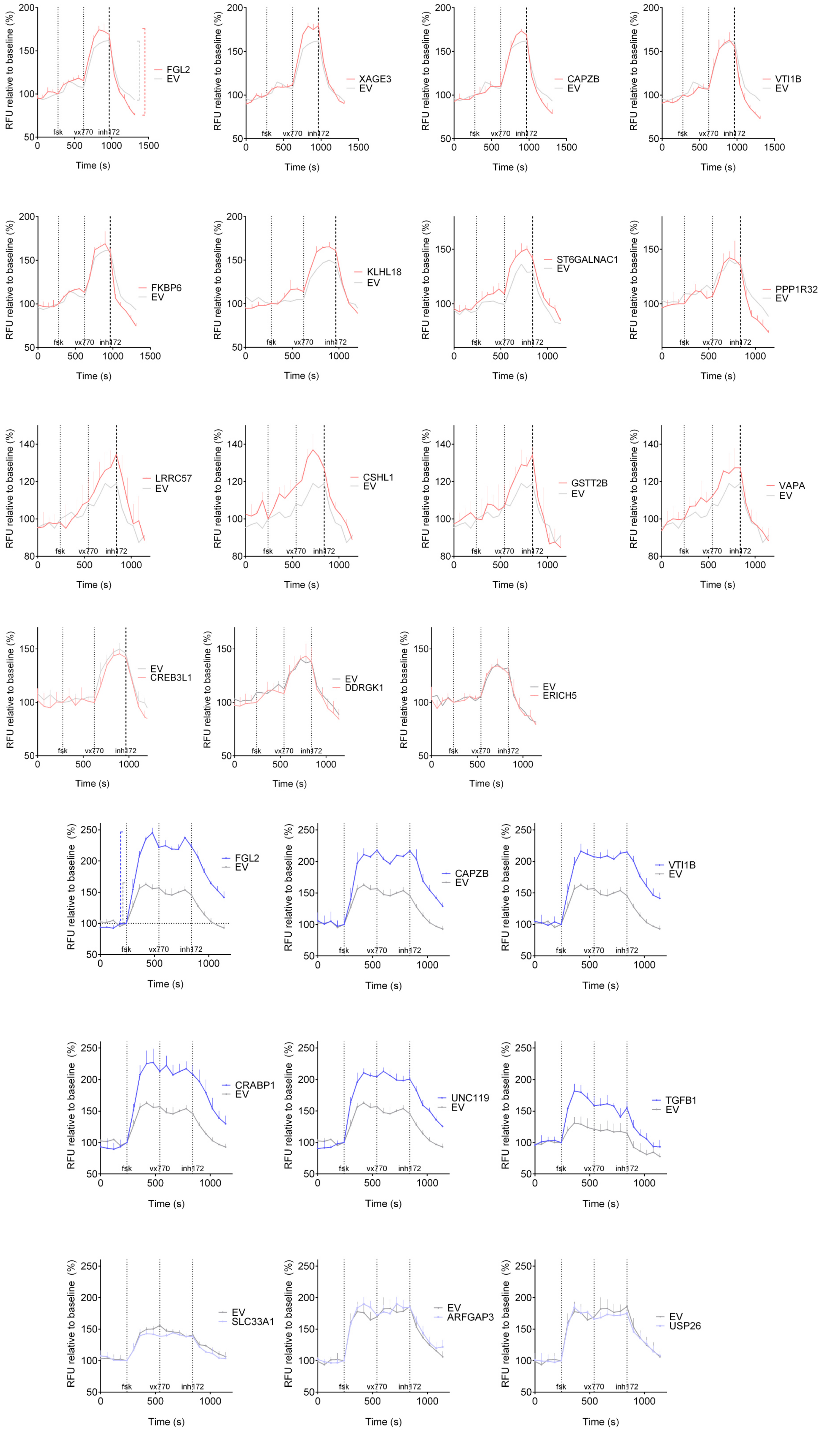

Appendix Figure S7 – FLIPR membrane potential traces for interactors whose transient expression in HEK293 cells affects CFTR channel activity. A) Interactors affecting F508del-CFTR channel activity. B) Interactors affecting wt-CFTR channel activity. Three select representative traces for interactors whose expression did not affect channel activity are also shown for each.

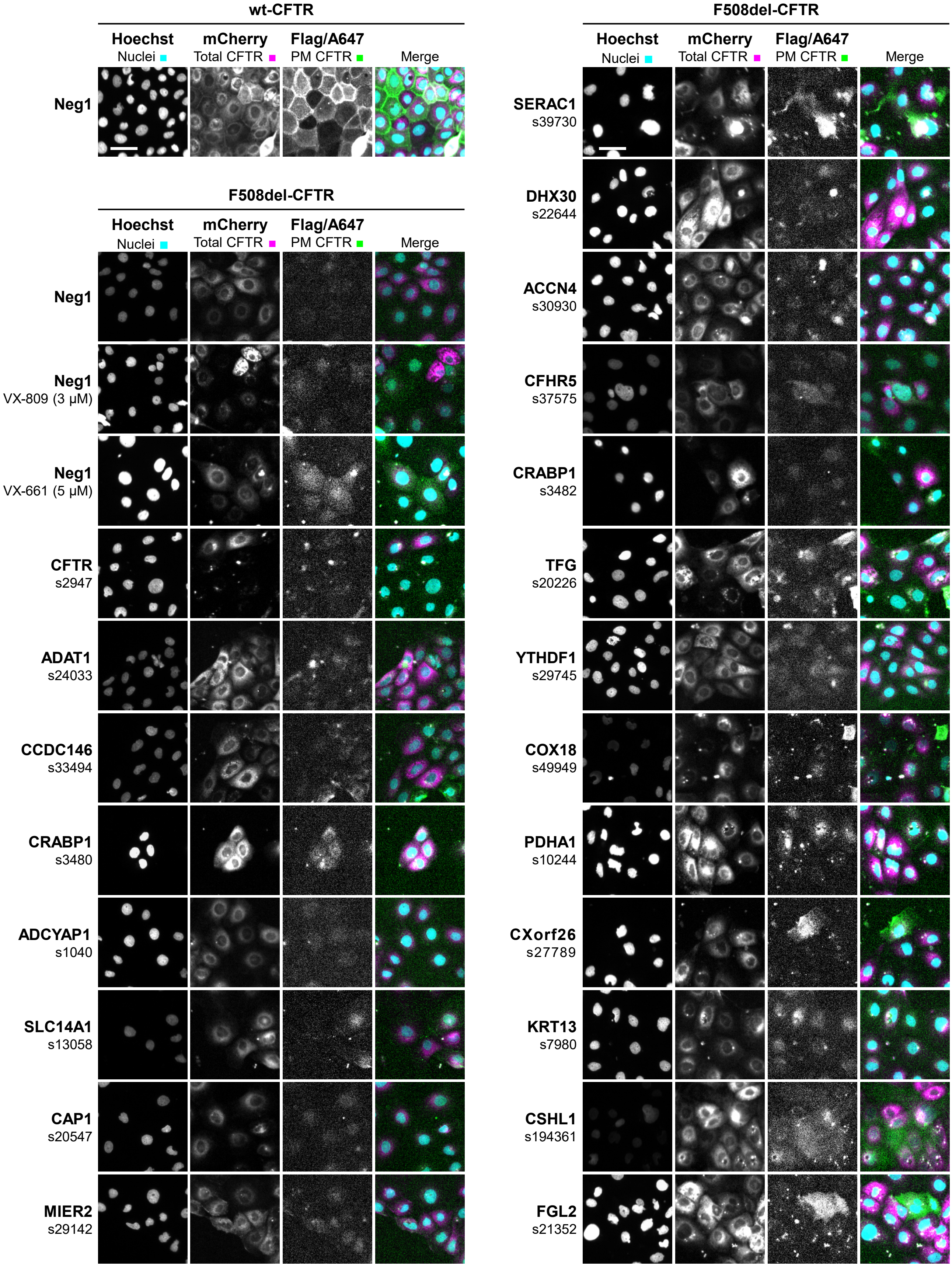

Appendix Figure S8 – **Hit siRNAs in the high-content CFTR trafficking assay.** Representative images of CFBE cells expressing the wt- or F508del- variants of the mCherry-Flag-CFTR traffic reporter. Hoechst stains nuclei, mCherry labels all CFTR molecules and immunostained Flag in unpermeabilized cells labels CFTR molecules located at the PM. Cells treated with the non-targeting Neg1 siRNA or F508del-CFTR correctors (VX-809, VX-661) were taken as negative or positive controls, respectively. Significant loss of CFTR expression in cells treated with a CFTR-targeting siRNA indicates high transfection efficiency. The remaining conditions represent cells treated with siRNAs which rescue F508del-CFTR PM localization (Z-score > 1), as seen by increased fluorescence intensity in the Flag channel versus the negative control. One of the siRNAs targeting FGL2, which did not meet the Z-score hit threshold in the traffic screen, is also shown. Corresponding channels are shown in the same lookup table, except for wt-CFTR expressing cells, where contrast required adjustment due to the overall larger fluorescence intensity. Scale bar: 50 µm.

A)

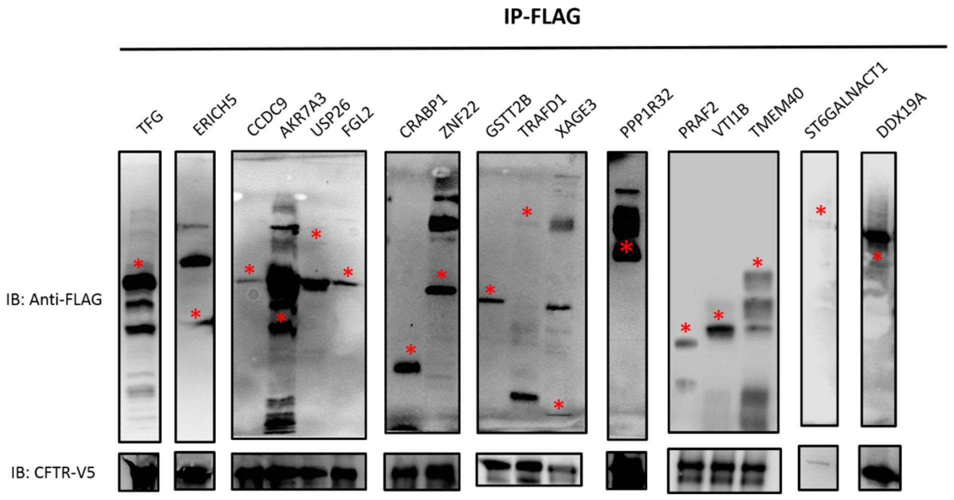

B)

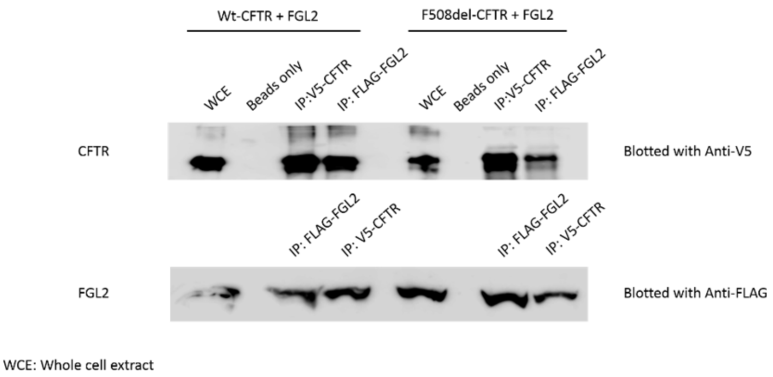

C)

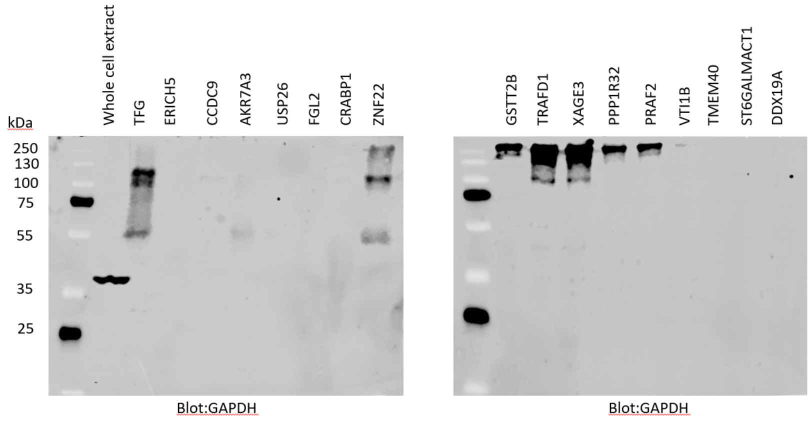

Appendix Figure S9 – Validation of selected interactions via co-immunoprecipitation (Co-IP) in HEK293T cells. A) Co-IP experiments showing 17 successfully validated interactions (out of 30 tested). Co-IPs were performed using anti-FLAG antibody directed against overexpressed FLAG-tagged protein corresponding to identified interactors, followed by Western blotting using anti-FLAG antibody (top panels) and anti-V5 antibody directed against the V5-tagged wildtype CFTR (lower panels). Red asterisks indicate bands of the size corresponding to the FLAG-tagged interactor protein being tested. Images are representative of n=2 biological replicates. B) Co-IPs performed to confirm the FGL2/CFTR interaction. Pull-downs were performed using anti-V5 antibody, followed by Western blotting with anti-V5 and anti-FLAG antibody or using anti-FLAG antibody, followed by Western blotting with anti-FLAG and anti-V5 antibody, as labelled. C) Control anti-FLAG co-IPs for all tested interactors, blotted with unrelated anti-GAPDH antibody, showing that over-expressed FLAG-tagged interactors do not non-specifically pull-down unrelated protein.

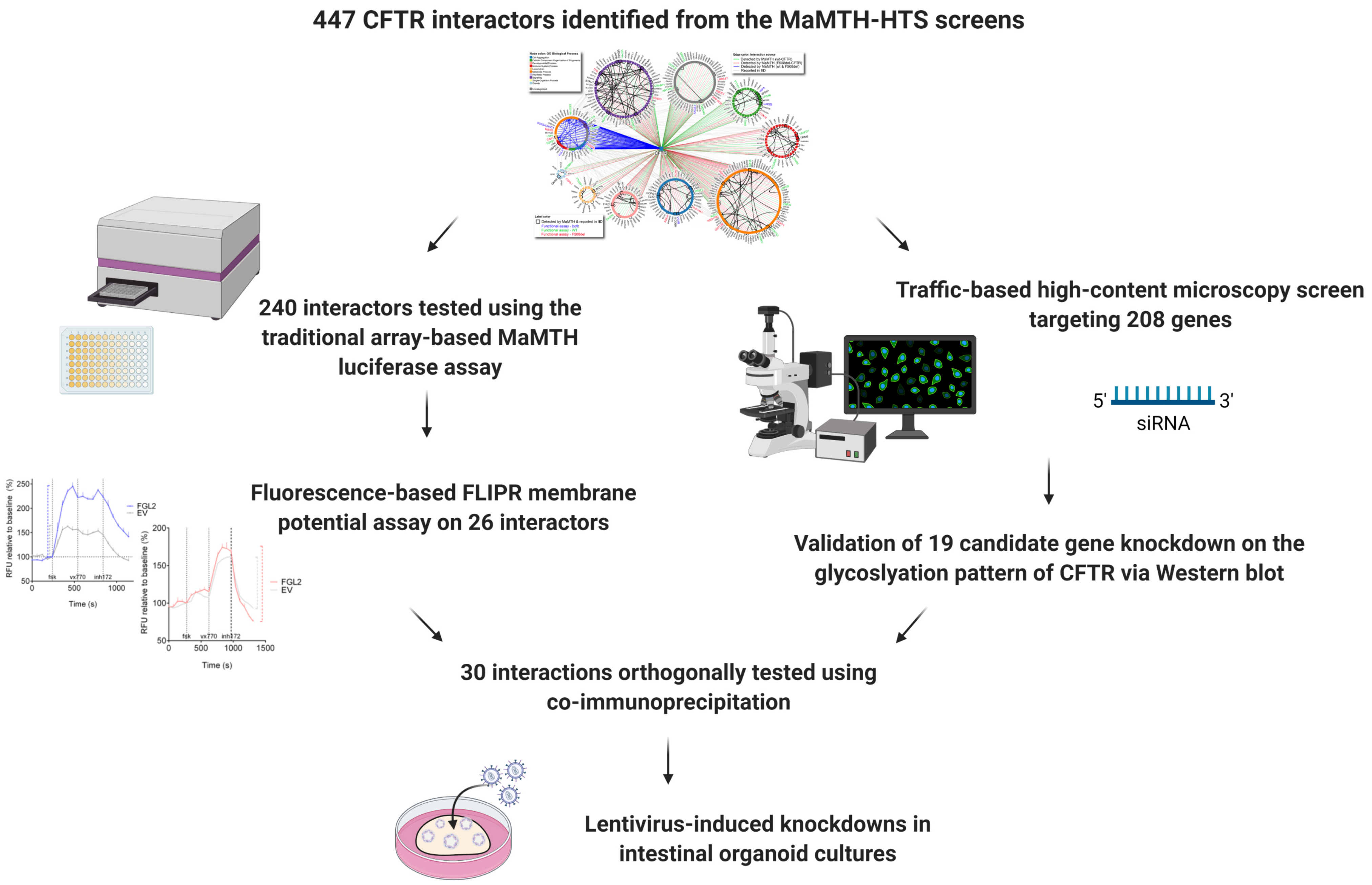

Appendix Figure S10 – Overview of the validation strategy used in this work. Created with BioRender.com.
