# Supplementary material for: CFTR interactome mapping using the mammalian membrane two‐hybrid high‐throughput screening system
Source: Mol Syst Biol. 2022 Feb 14;18(2):e10629. doi: 10.15252/msb.202110629 (PMC8842165; doi:10.15252/msb.202110629)
Supplement: Supplementary file 13 [file MSB-18-e10629-s013.pdf]

molecular systems biology

CFTR Interactome Mapping Using the Mammalian Membrane Two-Hybrid High-Throughput Screening System

Sang Hyun Lim, Jamie Snider, Liron Birimberg-Schwartz, Wan Ip, Joana Serralha, Hugo Botelho, Miquéias Lopes-Pacheco, Madalena Pinto, Mohamed Moutaoufik, Mara Zilocchi, Onofrio Laselva, Mohsen Esmaeili, Max Kotlyar, Anna Lyakisheva, Priscilla Tang, Lucía López Vázquez, Indira Akula, Farzaneh Aboualizadeh, Victoria Wong, Ingrid Grozavu, Teuta Opacak-Bernardi, Zhong Yao, Meg Mendoza, Mohan Babu, Igor Jurisica, Tanja Gonska, Christine Bear, Margarida Amaral, and Igor Stagljar
DOI: 10.15252/msb.202110629

Corresponding author(s): Igor Stagljar (igor.stagljar@utoronto.ca)

Review Timeline:

Submission Date:	5th Aug 21
Editorial Decision:	10th Sep 21
Revision Received:	21
Editorial Decision:	7th Jan 22
Revision Received:	16th Jan 22
Accepted:	19th Jan 22

Editor: Maria Polychronidou

Transaction Report:

(Note: With the exception of the correction of typographical or spelling errors that could be a source of ambiguity, letters and reports are not edited. Depending on transfer agreements, referee reports obtained elsewhere may or may not be included in this compilation. Referee reports are anonymous unless the Referee chooses to sign their reports.)

Thank you again for submitting your work to Molecular Systems Biology. We have now heard back from the three referees who agreed to evaluate your study. Overall, the reviewers acknowledge that the study is a relevant contribution to the field. They raise however a series of concerns, which we would ask you to address in a major revision.

I think that the reviewers' recommendations are clear and I therefore see no need to repeat the points listed below. Most issues refer to the need to include additional controls, some further experimental validations, as well as biological replicates, to better support the main conclusions. All issues raised by the reviewers need to be satisfactorily addressed. Please contact me in case you would like to discuss in further detail any of the issues raised.

On a more editorial level, we would ask you to address the following points:

Reviewer #1:

Review of Lim et al "CFTR Interactome Mapping Using the Mammalian Membrane Two-Hybrid High-Throughput Screening System"

This study describes an mammalian membrane two-hybrid (MaMTH) based protein focusing on interactions with the membrane protein CFTR and the most frequently found disease variant CFTR(F508del) that is causing Cystic fibrosis in about 70% of the patients.
For the first time MaMTH is applied on a proteome scale to screen a large human ORFeome libaray that well covers the human

proteome. To this end MaMTH was adapted to HTP, including the generation of a stable ORFeome prey library as a pool and switching to fluorescence readouts in order to FACS separate interacting from non-interacting "cells" that are subject to second generation sequencing for prey identification. An interaction network that involves 447 candidate interactions with 224 interacting with wt and 296 interacting with the mutant CFTR. The very surprising finding is that there is only a 10% overlap between CFTR and CFTR(F508del) mutant protein interactions. A series of validation experiments is performed on what appears to be more or less random sets of interaction partners. It involves channel activity assays, standard luciferase based MaMTH experiments, Co-IPs, RNAi based trafficking and CFTR stability/glycosylation assays and finally demonstrating the effects of interaction partner FGL2 shRNA mediated perturbation in intestinal organoids, altered swelling and transepithelial potential. As such I agree with the authors that they present preliminary functional characterization in a number of instances "providing evidence supporting interesting links between some interactors and CFTR function". The study is data rich, very original in its approach and will become a key resource for studying the effects of CFTR variants.
It is well known that many CFTR mutations influence processing, stability and localization of the receptor as well as altered channel activity and the hypothesis is that also protein interaction changes accompany or even modulate these effects. This may not only be relevant for fully modified properly membrane located CFTR. Therefore the finding that the interaction partner are substantially different between the two version is very interesting.
What remains unclear is were the differences in the MaMTH-HTP network between the two proteins come from considering that there is a one amino acid difference between the two. Generally speaking, in the validation experiments this specificity between wt and mutant is not seen. Just as example of what I mean: CAPZB is a mutant specific interactor and decreases trafficking of both wt and mutant. This kind of results are listed in data S2 (not in full as I believe), but need to be addressed comprehensively in the main body of the paper.

Points for consideration:
*) The localization of the two transiently expressing receptors is not presented. Are they actually localized at different compartments or in the membrane?
*) It is not well described how a interaction is called from the seqcounts. How is the enrichment against the background actually determined? Cf. "analyzed using in house bioinformatics software"
*) The Venn diagrams in Figure S3 look very impressive from the overlap point of view. However, it remains unclear what the replicas really represent, in the main text it says technical replicas. What is it and more importantly were biological replicas performed?
*) As mentioned above, interaction specificity has to be put in the context of the validation experiments. For example the FLIPR assay, what was tested, why do we see ARFGAP3, a F508delta interactor in the wt assay but not in the delta assay. There is no effect on the wt, which is also not annotated in data S2.
*) What was the validation strategy, which candidates have been tested (with either wt or mutant or both) and what is the role of wt vs mutant binding?
*) Data S2, critical to get on overview of all the data but I also think the separation in the two sets of interactors in this table is not very useful as the various proteins obviously have been tested across both CFTR wt and mutant receptors and therefore results like ARFGAP3 are not recorded in the table.
*) It looks as if the interaction specificity seen in the MaMTH-HTP is related to the assay, so therefore the question about biological replicas becomes a pivotal one (=> false negatives). This is not to criticize the approach as such, however the interpretation such as the presentation of the interaction network in Figure 2 that emphasizes this specificity may be strongly misleading.
*) Figure 4: Z scores are displayed, however what is the N and what is the N of the reference (siNeg1).
*) Figure 6 C: borders of the 4x2 images can not be recognized.

Reviewer #2:

Lim et. al. employ MaMTH-HTS to identify novel PPIs with wildtype CFTR as well as its most clinically-observed mutant, the CFTR F508del, which has been demonstrated to play a role in the pathophysiology of cystic fibrosis. The current variant of their previously published MaMTH method now uses transcriptionally-activated fluorescence-based sorting of live cells to identify populations wherein both bait and prey expression are verified, followed by a final sort documenting the interaction of the two proteins, which then can be deep sequenced to identify the candidate PPI partners. This adaptation of their original method allows for higher throughput and allowed the authors to probe the entire Human ORFome to identify candidate interactors with either wildtype or mutant CFTR. Once these PPIs were identified, the authors used a subset of the identified interactions to characterize their functional role in CFTR channel activity, localization, and confirm their interaction with CFTR using co-immunoprecipitation. Overall, the manuscript provides a technical advancement on their screening technique and the preliminary findings of several novel PPIs affecting CFTR (wildtype or mutant) function. However, the manuscript could be improved by addressing the following critiques:
1. While the introduction provides detailed information about the pathological features of cystic fibrosis, it lacks sufficient background about the MaMTH-HTS platform employed to explore the CF interactome. By adding the rationale of why this platform is superior to other PPI screening techniques, the reader would gain valuable knowledge upfront that supports the study design.
2. The authors might consider including the screening vin diagrams (SF3, as part of Figure 2) for clarity.
3. The study is actually weakened by the attempt to compare MaMTH-HTS with the original MaMTH assay. While the authors try

to address the variables that could lead to the observed differential results, this section of the manuscript adds confusion and ambiguity to the results that do not support the overall conclusion of the manuscript and may disparage the robustness of the approach for some readers. What is truth? Instead, it might be suggested to follow a similar approach to the presentation of the validation of HITs herein as was presented in Figure 5a of the original MaMTH manuscript (Petschnigg et al 2014); these data can be incorporated without needing to present the data as a head to head comparison in the main body, especially given the differences as discussed. Assay comparisons, while relevant, could then be moved to the Supplemental data section.
4. When addressing potential concerns between methodologies used to interrogate the CFTR interactome, the authors highlight differences in results and techniques, but do not provide a rationale or hypothesis for these discrepancies (specifically for mass spec analysis). The manuscript would be strengthened by the addition of this context.
5. The manuscript would be enhanced by the addition of representative images of CFTR localization altered by key PPIs (FGL2, etc.) identified during the traffic screening study and subsequent validation experiments.
6. The authors use the discussion to comment on several PPIs identified by MaMTH-HTS. While this information is interesting, it failed to support the thesis of the manuscript as presented. The authors might consider re-writing the discussion such that they highlight the potential of these novel interactions identified and then select a few key points to give specific examples and details, pulling in the connections already identified in the literature. Fundamentally, is the manuscript intended to be a report of the new method or a biology report on the novel interactions identified for the CFTR field or both? Seek a consistent tone throughout.
Minor points and grammatical suggestions:
1. The traditional MaMTH assay in the methods section is missing the complete reference.
2. Some text refers to the library as >10,000 ORFs and other places >12,000 ORFs. Correct to render uniformity.
3. Figure 6B-F, the naming strategy of the plots does not match previous studies. This should be checked for all figures.
4. Text on Figure S6 is very small. You could consider having the axis be consistent and then including larger text for just one panel.
5. Text related to Figure 6F. It would be more intuitive to refer to the tracing as current (as illustrated) rather than transmembrane potential in the text when discussing the Ussing chamber data.
6. Page 16, "...growing interest as a therapeutic "target" in treating autoimmune disorders and transplant rejection..."

Reviewer #3:

Lim, Snider et al. describe the development of a high throughput version of the Mammalian Membrane Two Hybrid (MaMTH-HTS) which they use to screen CFTR and a mutant variant of the protein (CFTR (F508del) for interaction partners. The introduction of fluorescent proteins to monitor bait and prey expression, and to evaluate the interaction between the partners enabled screening of the complete ORFEOME 8.1 collection by FACS and deep sequencing on the sorted cells. A series of downstream functional assays and an orthogonal validation test are performed to validate the candidate interactors. Although early stage, the data suggests new relevant biology with therapeutic potential. I appreciate the discussion which addresses the comparison between the HTS and the traditional format and which also addresses some of the issues with the validation (e.g. with the siRNA screen).
I have the following major comments:
As the authors point out, the overlap between the traditional MaMTH and this novel HTS version is not that high. While the authors provide some explanation for this, it does raise some questions. The manuscript mentions technical replicates of the MaMTH-HTS experiments. At what stage in the protocol are these replicates taken? This will allow a better assessment of the robustness of the experiments. Biological repeats will also allow a better assessment. Some more repeat experiments may help in understanding where this limited overlap comes from and may provide a clue on the size of the interactome as assessed by MaMTH and its HTS variant.
Furthermore, can the authors comment on a relation between the intensity of the MaMTH signal and the strength of an interaction. Is this a criterium for selection? In both reporter assays, i.e. in a classical luciferase read-out, and in this EGFP read-out? Related to this: EGFP is known to be highly stable, and is therefore likely not the best reporter because of this. Did the authors notice any accumulation problems with EGFP (background issues?) or were any steps taken to prevent that?
Perturbance by siRNA is indeed a powerful approach to assess a number of candidates coming from a PPI screen. While a number of candidates seem to have an effect when treated with siRNA, there is only one candidate where the effect is confirmed with two independent siRNAs for the same condition. While it is clear that not all siRNAs have the same efficiency (as pointed out also by the authors), this is still a striking observation. At least some of these siRNAs should show independent validation. The assay used to measure the siRNA effect appears to be specific, but it cannot be excluded that off-target effects of the siRNAs are actually observed here (rightfully discussed by the authors as a lack of specificity). The authors should therefore consider to add a third siRNA for (part of) the positive candidates to obtain more evidence for these candidates as to increase the evidence.
The Co-IP studies are a powerful orthogonal approach to complement the MaMTH assay and the number of confirmed interaction is high considering the limited overlap typically obtained with orthogonal approaches. However, controls without antibody (or with irrelevant antibody) for CFTR (i.e. V5 tag) appear missing (bead only). Some of the interactions may therefore be aspecific and it is best practice to include these controls to show a clear bait-dependent enrichment. While more controls have been included for the reciprocal Co-IP of FGL2 (not the bead control for V5 however), both partners were introduced by overexpression. Co-IP for the endogenous proteins would obviously be better and a very strong argument for the interaction but may be challenging because the endogenous CFTR needs to be extracted from the membrane fraction.

For the validation studies and the repeat experiments, it is also not always clear how the test sets were selected. For Co-IP 'a selected subset of 30 CFTR-interacting proteins was used', a 'large subset' was used for traditional MaMTH, a subset of 26 was selected based on performance for the FLIPR assays, and 208 genes were used for siRNA screening. This seems somewhat random and should be explained better. Related to this, an overview scheme for the manuscript would help to create some structure.
Taken together, this is definitely an interesting upgrade of the technology which is applied to an interesting biological question, but the differences between the different MaMTH variants combined with some questions around the validation data, keep me from accepting the manuscript at this stage. A more convincing case study around one of the partners or a more robust validation assessment of a test set is needed in my opinion.

Minor comments:
Check references! Some only contain first letter of the names.
It would be good to provide some images in suppl info that go with the high-content trafficking assay.
Add time dimension to schematic in Figure 6A.
The organoid experiments are an elegant approach to show the role of FGL2. Some clarification on these experiments would be good to allow a better assessment. Can the authors comment on the effects on FGL2 upon KD of CFTR (and vice versa; Fig 6B)? The higher levels seem to suggest some interplay at the transcriptional level.
Figure 6B: Indicate what EV stands for in figure legend (Empty Vector?!).

Reviewer #1:

*) The localization of the two transiently expressing receptors is not presented. Are they actually localized at different compartments or in the membrane?

The transiently expressing wildtype and F508del CFTR membrane proteins are localized at different compartments in their native states. The wildtype CFTR is mainly localized in the plasma membrane as an anion channel while the F508del mutant spends most of its lifetime in the ER membrane and becomes targeted for degradation by the quality control. We have now included a figure (**Appendix Figure S3**) showing that transiently expressed wt and mutant CFTR proteins fused to a MaMTH-bait tag and GFP are localized a s expected. The observed behaviour of the channel in our Fluorescence-based assays using MaMTH tagged CFTR baits further provides evidence of the proper localization of the transiently expressing CFTR proteins.

*) It is not well described how a interaction is called from the seqcounts. How is the enrichment against the background actually determined? Cf. "analyzed using in house bioinformatics software"

For the purpose of this study, we did not determine enrichment against background, but instead looked at what ORFs were present after sorting (based on alignment of sequencing results against the Human ORFeome v8.1 collection, removing any extremely poorly represented ORFs with total counts below 3 in both replicates). To eliminate/reduce background, non-specific 'frequent fliers' were then removed from these lists based on their occurrence across screens of a control set of diverse protein types. Note that we originally tried enrichment-based analysis but did not find this provided any notable advantage in data quality over the more straightforward approach used above. We have added additional detail to the methods section to clarify our analysis.

*) The Venn diagrams in Figure S3 look very impressive from the overlap point of view. However, it remains unclear what the replicas really represent, in the main text it says technical replicas. What is it and more importantly were biological replicas performed?

The replicates presented in Figure S3 (now **Appendix Figure S4**) represent PCR amplifications of genomic DNA from our sorted populations using two different sets of primers. These amplifications were then processed and deep sequenced separately. The purpose of this approach was to ensure that technical issues (PCR bias, inconsistency across sequencing runs etc.) were not affecting our results. We have clarified this in the text of the manuscript. In terms of biological replicates, while we aim to pursue this in the future, in the context of the resources available for the current study we were unfortunately unable to carry them out at this time. We acknowledge (both here and in our manuscript) that non-specific interactions may thus be present in our interactome and are something to take into consideration, although we do hope that our use

of a set of control bait screens to identify frequent fliers has helped minimize these. Our primary goal in this publication, however, was more to focus on the interesting biological results we uncovered with our approach, rather than on the method itself, noting that the method is still very new and has to be more extensively benchmarked using different classes of proteins (something which is the subject of continuing studies).

*) As mentioned above, interaction specificity has to be put in the context of the validation experiments. For example the FLIPR assay, what was tested, why do we see ARFGAP3, a F508delta interactor in the wt assay but not in the delta assay. There is no effect on the wt, which is also not annotated in data S2.

We agree with the reviewer's comment that interaction specificity has to be in the context of the subsequent validation experiments. However, we noticed that there were many interactors whose interaction specificity towards the two CFTR baits differed between the MaMTH-HTS dataset and the array-based traditional MaMTH assay results, something which could potentially be a result of technical differences between the two approaches. Thus, in the validation experiments such as the Fluorescence-based membrane potential assay we tested interactors across both CFTR variants in some cases. We regret that this was not presented more clearly in our S2 dataset and have now restructured the dataset (as **Dataset EV8**) to better integrate the results for WT and mutant CFTR.

*) What was the validation strategy, which candidates have been tested (with either wt or mutant or both) and what is the role of wt vs mutant binding?

Our validation strategy first involved re-testing a large subset (240) of the 447 CFTR interactors identified from the MaMTH-HTS screens (**Figure 2**) using the traditional array-based MaMTH assay. From there, we selected 26 interactors based on their interaction signal strength and consistency in their interaction profile between the two methods. Note that we included both cases where traditional and HTS interactors overlapped and where they did not, focusing largely on interactions confirmed in traditional MaMTH for both WT and mutant (but including some instances where this was not the case for sample diversity). The 26 interactors were then tested in the fluorescence-based FLIPR assay to monitor their effect on the channel activity. Concomitantly, we performed the traffic-based siRNA microscopy screen, targeting 208 genes (based on their availability in the siRNA library) from our original 447 interactors to investigate the trafficking of CFTR to the plasma membrane in the absence of these discovered interactors. This high-content screen was combined with Western blot experiments for validating the post-translational glycosylation pattern of CFTR. From these experiments, we performed final orthogonal validation on some of the most interesting interactors using a co-IP approach and followed up with organoid work. To better summarize our validation approach, we have now included the above description as a **'Validation Strategy'** section in the Methods, as well created a new **Appendix Figure S10**

providing a graphical overview. Note that our goal in this manuscript, which we believe we accomplished, was to identify interesting biological associations (as measured by effects on CFTR activity and regulation) suitable for investigation in future studies. As stated above, we have also now restructured our S2 dataset (as **Dataset EV8**) to provide a clearer summary and better overall view of this data in the context of each candidate interaction.

*) Data S2, critical to get on overview of all the data but I also think the separation in the two sets of interactors in this table is not very useful as the various proteins obviously have been tested across both CFTR wt and mutant receptors and therefore results like ARFGAP3 are not recorded in the table.

We have redone the S2 dataset (now **Dataset EV8**), integrating the WT and mutant results and making some descriptions more explicit in order to make things clearer.

*) It looks as if the interaction specificity seen in the MaMTH-HTP is related to the assay, so therefore the question about biological replicas becomes a pivotal one (=> false negatives). This is not to criticize the approach as such, however the interpretation such as the presentation of the interaction network in Figure 2 that emphasizes this specificity may be strongly misleading.

We appreciate the reviewer's comments and agree that our assay (like any other high throughput screen) does potentially have issues with false positives and negatives, particularly since it is very new and not yet extensively benchmarked, something which we freely acknowledge in the text of our manuscript (both in our initial and revised drafts). While this could be addressed through biological replication, we were unfortunately unable to perform this due to time and resource constraints. However, we did attempt to address these potential issues and demonstrate the quality of our approach through the use of a control set of baits to identify frequent fliers, comparison of our HTS approach to traditional MaMTH and extensive validations (whereby we were able to detect a range of biologically interesting candidate interactions). Upon careful consideration of the reviewer's comments, however, we do see how the emphasis on WT vs mutant specificity in **Figure 2** could be misleading. We have therefore generated a **revised Figure 2** reducing this emphasis. While all previous information is still provided, we hope this new layout presents a clearer, less-biased view of the interaction data.

*) Figure 4: Z scores are displayed, however what is the N and what is the N of the reference (siNeg1).

Four independent biological replicates were performed, as indicated in the Methods section ("CFTR traffic assay"). However, due to the quality control parameters (also reported in Methods subsection "High-content image acquisition and analysis"), not all siRNAs could be analyzed at all four replicas. The number of valid replicates has now been added to both Table S7 (now **Dataset**

EV7) and **Figure 4**. Each assay plate contained 7 or 18 reference wells (siNeg1) and this information has been added to the Methods ("Preparation of siRNA coated multi-well plates").

*) Figure 6 C: borders of the 4x2 images can not be recognized.

Correction has been made so that the borders can be recognized in the confocal images.

Reviewer #2:

1. While the introduction provides detailed information about the pathological features of cystic fibrosis, it lacks sufficient background about the MaMTH-HTS platform employed to explore the CF interactome. By adding the rationale of why this platform is superior to other PPI screening techniques, the reader would gain valuable knowledge upfront that supports the study design.

We thank the reviewer for their comment but want to stress that we are not making the claim that our current form of the MaMTH-HTS method is superior to other PPI screening techniques, as it is still very new and its performance has not yet been rigorously benchmarked with other human integral- and/or membrane-associated proteins. The goal of our current publication is to report some interesting (and potentially important) biological results we have obtained for CFTR during the development of our novel high-throughput screening platform for human membrane proteins. Following the reviewer's comment, however, we have now tried to better emphasize some *potential* advantages of the technology in the results section of the manuscript under the 'Design of the MaMTH-HTS Platform' heading.

2. The authors might consider including the screening vin diagrams (SF3, as part of Figure 2) for clarity.

We appreciate the reviewer's suggestion, however we are concerned that **Figure 2** (which we have also revised in this latest version of our manuscript) is already very busy and attempting to include additional data could make it harder to interpret. After careful consideration we therefore believe it is best to keep **Figure 2** and SF3 (now **Appendix Figure S4**) separate.

3. The study is actually weakened by the attempt to compare MaMTH-HTS with the original MaMTH assay. While the authors try to address the variables that could lead to the observed differential results, this section of the manuscript adds confusion and ambiguity to the results that do not support the overall conclusion of the manuscript and may disparage the robustness of the approach for some readers. What is truth? Instead, it might be suggested to follow a similar approach to the presentation of the validation of HITs herein as was presented in Figure 5a of the original MaMTH manuscript (Petschnigg et al 2014); these data can be incorporated without needing to present the data as a head to head comparison in the main body, especially given the

differences as discussed. Assay comparisons, while relevant, could then be moved to the Supplemental data section.

We appreciate the reviewer's comments and do understand the concerns they raise. However, we feel that the comparison to traditional MaMTH is important to focus on, as MaMTH-HTS is still very new and has not yet undergone extensive benchmarking, something we mentioned in the previous version of the manuscript but have now attempted to stress even more clearly in our revision. As such we want to be as transparent as possible about our new method and highlighting how it contrasts with our previous approach is part of that, particularly since both approaches have revealed biologically interesting interactions in this work. Importantly, we want to stress that our goal with this manuscript was not to focus primarily on MaMTH-HTS, but rather to highlight the interesting biological results relevant to CFTR/CF we have obtained during our development of the assay and demonstrate the *potential* of our new approach to find previously unreported interactions of biological relevance. We acknowledge that there is still clearly work to be done, and this will of course be the focus of future studies.

Additionally, we reviewed Figure 5 from our earlier manuscript, and while it worked well for our data there, the nature of the readout in MaMTH-HTS (i.e., in that we haven't really focused on a quantitative readout) and the number of interactions/overlaps makes it challenging. We have, however, revised our Data S2 (now **Dataset EV8**) to better summarize all of the relevant interaction details and the results obtained in traditional MaMTH and our other validation experiments, to make it more conveniently accessible to readers.

4. When addressing potential concerns between methodologies used to interrogate the CFTR interactome, the authors highlight differences in results and techniques, but do not provide a rationale or hypothesis for these discrepancies (specifically for mass spec analysis). The manuscript would be strengthened by the addition of this context.

We have tried to better explain in the discussion why we may see differences in our approaches vs mass spec analysis.

5. The manuscript would be enhanced by the addition of representative images of CFTR localization altered by key PPIs (FGL2, etc.) identified during the traffic screening study and subsequent validation experiments.

The revised manuscript now contains a new **Appendix Figure S8** containing representative images of the high-content screening results showing the sub-cellular localization of wt-CFTR, as well as F508del-CFTR after treatment with several siRNAs (i.e., those assayed by Western blot).

6. The authors use the discussion to comment on several PPIs identified by MaMTH-HTS. While this information is interesting, it failed to support the thesis of the manuscript as presented. The

authors might consider re-writing the discussion such that they highlight the potential of these novel interactions identified and then select a few key points to give specific examples and details, pulling in the connections already identified in the literature. Fundamentally, is the manuscript intended to be a report of the new method or a biology report on the novel interactions identified for the CFTR field or both? Seek a consistent tone throughout.

We thank the reviewer for their comments and are sorry if the thesis of our manuscript was unclear. While we have presented our new method, our goal here was not to focus on it extensively (as MaMTH-HTS is still new and in the process of being benchmarked) but rather to highlight some interesting CFTR interactions we identified with it which appear to be of biological importance. In our discussion, we believe we have accomplished this by highlighting select interesting hits, referencing key literature relating to their function and briefly discussing possible details of their roles in CFTR biology. While we would obviously like to pursue these in greater depth, more extensive experimental investigation of these hits is unfortunately beyond the scope of our current work and available resources (but will of course be the subject of future studies). In light of the reviewer's comment, however, we have tried to better clarify our goal in the manuscript.

Minor points and grammatical suggestions:

1. The traditional MaMTH assay in the methods section is missing the complete reference.

We have reviewed the references and made corrections throughout.

2. Some text refers to the library as >10,000 ORFs and other places >12,000 ORFs. Correct to render uniformity.

Note that although the original library contained approximately 12,000 ORFs, as mentioned in our section describing library generation, we were only able to clone a little over 10 000 of these (see **Dataset EV1**). Hence the use of both numbers at different points in the manuscript. We apologize for any confusion.

3. Figure 6B-F, the naming strategy of the plots does not match previous studies. This should be checked for all figures.

The naming strategy in **Figure 6** has been unified to be more consistent across the figure. Note that differences in nomenclature between the samples used in **Figure 6** and in our other data is a reflection of the unique nature of the organoids, which are freshly derived from patient samples.

4. Text on Figure S6 is very small. You could consider having the axis be consistent and then including larger text for just one panel.

We have modified the text size in Figure S6 (now **Appendix Figure S7**) to try and improve clarity.

5. Text related to Figure 6F. It would be more intuitive to refer to the tracing as current (as illustrated) rather than transmembrane potential in the text when discussing the Ussing chamber data.

We have changed in the text to refer the tracing as current.

6. Page 16, "...growing interest as a therapeutic "target" in treating autoimmune disorders and transplant rejection..."

Correction has been applied.

Reviewer #3:

I have the following major comments:
As the authors point out, the overlap between the traditional MaMTH and this novel HTS version is not that high. While the authors provide some explanation for this, it does raise some questions. The manuscript mentions technical replicates of the MaMTH-HTS experiments. At what stage in the protocol are these replicates taken? This will allow a better assessment of the robustness of the experiments. Biological repeats will also allow a better assessment. Some more repeat experiments may help in understanding where this limited overlap comes from and may provide a clue on the size of the interactome as assessed by MaMTH and its HTS variant.

We thank the reviewer for their comment and have now provided a bit more detail on the technical replicates in the text and methods. In terms of biological replicates, while we intend to pursue this in the future, in the context of the resources available for the current study we were unfortunately unable to carry them out at this time. We acknowledge, both here and in our manuscript, that non-specific interactions and potential false negatives may therefore be present in our interactome but have sought to address these using the results of control screens across a diverse set of 9 baits to identify frequent fliers, performing the comparison to traditional MaMTH and by carrying out numerous functional validations (identifying a range of interactions of potential biological relevance to CFTR/CF). Our approach in this manuscript is more to focus on the interesting biological results that our method was able to uncover (and demonstrate the potential of our method to find such interactions), rather than specifically on the method itself, noting that MaMTH-HTS is still new and has not yet been extensively benchmarked (which we freely acknowledge in the text). We hope and believe that the data as presented constitutes a useful

contribution to proteomics and CF research, and stress that further benchmarking and advancement of the MaMTH-HTS technology will be the goal of upcoming studies.

Furthermore, can the authors comment on a relation between the intensity of the MaMTH signal and the strength of an interaction. Is this a criterium for selection? In both reporter assays, i.e. in a classical luciferase read-out, and in this EGFP read-out? Related to this: EGFP is known to be highly stable, and is therefore likely not the best reporter because of this. Did the authors notice any accumulation problems with EGFP (background issues?) or were any steps taken to prevent that?

In both the case of luciferase and GFP read-outs signal intensity does have some correlation with interaction strength, although this is less robust in the case of GFP. The reviewer makes an excellent point about eGFP, however, and its stability does indeed tend to generate relatively high background in some cases, something which we sought to address during assay development through control of sorting parameters and selection of interactions with higher intensity signal (based on comparison to ‘negative’ controls). We feel that the issue has been manageable in most instances (such as with our CFTR screens), although as part of our actively ongoing development of MaMTH-HTS we are exploring alternative readouts (including an ‘unstable’ mutant of eGFP), in an effort to reduce background.

The authors should therefore consider to add a third siRNA for (part of) the positive candidates to obtain more evidence for these candidates as to increase the evidence.

We appreciate the reviewer’s comment, however we feel that the request for validation with a third siRNA is outside the scope of this work. The goal of an siRNA screen – such as the high-content CFTR traffic assay included in this manuscript – is to narrow down a screening library to a subset of relevant hits, even if that limits the information extracted from sub-optimal or non-hit conditions, and consequently increasing the rate of false negatives. As we stress in the discussion (and noted by the reviewer) the confirmation rate (~30%) is in line with that usually obtained in similar studies and thus is no reason for concern. We must point out that the siRNA screen was performed as a validation step for MaMTH-HTS hits, and it was followed up with Western blot validation, to provide extensive orthogonal validation to the original MaMTH-HTS dataset. Moreover, performing additional experiments would be very time and effort-consuming and, importantly, have no guarantee of better / different results.

The Co-IP studies are a powerful orthogonal approach to complement the MaMTH assay and the number of confirmed interaction is high considering the limited overlap typically obtained with orthogonal approaches. However, controls without antibody (or with irrelevant antibody) for CFTR (i.e. V5 tag) appear missing (bead only). Some of the interactions may therefore be aspecific and it is best practice to include these controls to show a clear bait-dependent enrichment. While more

controls have been included for the reciprocal Co-IP of FGL2 (not the bead control for V5 however), both partners were introduced by overexpression. Co-IP for the endogenous proteins would obviously be better and a very strong argument for the interaction but may be challenging because the endogenous CFTR needs to be extracted from the membrane fraction.

We apologize for any confusion presented by our figure. Please note that the samples in panels S7A and S7B (now **Appendix Figure S9A** and **S9B**) were separated to illustrate our slightly more in-depth analysis of the FGL2 interaction, however the experiments were performed in the same way, and therefore the 'Beads Only' control presented in S7B (now **S9B**) is applicable to the prey pull-downs in S7A (now **S9A**), demonstrating they do not non-specifically pull down CFTR. We have also now included a new panel (**Appendix Figure S9C**) using unrelated GAPDH antibody to illustrate that the overexpressed preys are not non-specifically pulling down unrelated proteins. As the reviewer mentioned, co-IP for the endogenous CFTR proteins is challenging, but remains a goal of our future studies, especially for examining the FLG2 interaction.

For the validation studies and the repeat experiments, it is also not always clear how the test sets were selected. For Co-IP 'a selected subset of 30 CFTR-interacting proteins was used', a 'large subset' was used for traditional MaMTH, a subset of 26 was selected based on performance for the FLIPR assays, and 208 genes were used for siRNA screening. This seems somewhat random and should be explained better. Related to this, an overview scheme for the manuscript would help to create some structure.

For our validation strategy we retested a large subset of the 447 CFTR interactors (240/447) we identified in our MaMTH-HTS screen using our traditional MaMTH-assay. Here we aimed to select as large a number of interactions as could be reasonably managed based on availability. From there, we then chose 26 interactors based on their interaction signal strength and consistency in their interaction profile between the two methods. We included both cases where traditional and HTS interactors overlapped and where they did not, focusing primarily on interactions confirmed for both wt and mutant in traditional MaMTH (though we included some instances where this was not the case for sample diversity). The 26 interactors were then tested in the fluorescence-based FLIPR assay to monitor their effect on the channel activity. At the same time, we performed the traffic-based siRNA microscopy screen, targeting 208 genes (based on their availability in the siRNA library) from our original 447 interactors to investigate the trafficking of CFTR to the plasma membrane in the absence of our interactors. We combined this high-content screen with Western blot experiments to validate the post-translational glycosylation pattern of CFTR. From these experiments, we performed final orthogonal validation on some of the most interesting interactors using a co-IP approach and followed up with organoid work. To better clarify this, we have now added a **'Validation Strategy'** section to the Methods, as well as a new **Appendix Figure S10** summarizing our approach.

Minor comments:

Check references! Some only contain first letter of the names.

We have reviewed the references and made corrections throughout.

It would be good to provide some images in suppl info that go with the high-content trafficking assay.

Representative images have now been included in our new **Appendix Figure S8**.

Add time dimension to schematic in Figure 6A.

Time dimension has been added to **Figure 6A**.

Can the authors comment on the effects on FGL2 upon KD of CFTR (and vice versa; Fig 6B)? The higher levels seem to suggest some interplay at the transcriptional level.

The increased transcript level of CFTR upon knockdown of FGL2 (and vice versa) in the patient-derived organoid experiments is indeed very interesting and demands further research to elucidate the exact mechanism of action behind the uncovered protein-protein interaction. It is puzzling to us how the knockdown of FGL2 in the healthy organoids leads to modest increase (~150%) in the CFTR mRNA transcript level yet results in substantially decreased CFTR activity as measured in both FIS assay and Ussing traces. The FGL2 mRNA transcript level in the healthy control also increases (~200%) in response to the CFTR knockdown.

This increase in the transcript level by the knockdown is amplified in the diseased organoids obtained from the F508del homozygous patient. Here we observe a significant increase (~400%) in the CFTR mRNA level upon FGL2 knockdown which may explain how some of the resulting mutant protein (with the help of the Trikafta treatment as well) makes it out to the plasma membrane to be functional and produce significant signals captured by either assay. We definitely agree with the reviewer that there is some interplay at the transcriptional level that requires further research, however we think that this is beyond the scope of our current manuscript.

It is interesting to note that the overexpression of FGL2, which was used in the MaMTH interaction screen and FLIPR functional assay, appears to augment the functional expression of both wildtype and F508del CFTR. Nevertheless, we observed enhanced functional expression of the F508del CFTR in the severe CF phenotype organoids with FGL2 knockdown. This demonstrates that the latest FDA-approved TrikaftaTM treatment may still benefit from synergistic therapeutic interventions such as modulation of FGL2 expression.

Figure 6B: Indicate what EV stands for in figure legend (Empty Vector?!).

The requested information has been added to the legend for **Figure 6B**.

Thank you again for sending us your revised study. We have now heard back from the two reviewers who were asked to evaluate the revised manuscript. As you will see below, while reviewer #1 is supportive, reviewer #3 remains somewhat skeptical about the level of validation of the MaMTH-HTS method and feels that the study would benefit from additional analyses further supporting the method's performance. They do nevertheless mention that the method is likely to be widely useful for future applications. Given that reviewer #3 acknowledges the potential value of the method and that reviewer #1 is quite supportive, we have decided to proceed with publishing the study in its current form. However, we would ask you to address the remaining concerns of reviewer #3 by ensuring that potential pitfalls etc. are clearly described in the manuscript. Of course, in case in the meantime you have acquired additional data further supporting the performance of MaMTH-HTS, we would strongly encourage you to include them in the manuscript.

Before we can formally accept the study for publication we would also ask you to address some minor editorial issues listed below.

Reviewer #1:

Lim et al. provided additional material including the localization of bait proteins and more extensive supplemental material summarizing the data. The focus on uncovering potential biological interactions using wt and mutant receptor is worked out more clearly now, putting less emphasis on PPI switches between the two version. I really do think that revised figure 2 is a much better representation of the interaction data in the light of the validation schema. Congratulations to this outstanding study!

Reviewer #3:

I thank the authors for clarifying the different points that I have raised and the manuscript has improved substantially. While I can follow the argumentation that the focus is more on the biology rather than on the method, the biological validation aspect has to be very robust and exciting, which actually then also implicitly supports the method. At this point I am not completely convinced of this. Based on this work, I would not perform the HTS-MaMTH method as it is not sufficiently supported by experimental data (as the authors agree themselves) and in addition has a different outcome when compared to the classical format. While I appreciate the honest presentation of the method as performed now, it does raise the issue on the value of the different candidates. For this reason, I believe that some extra work would really benefit the manuscript, either on the method or the validation side. siRNA experiments can be worthwhile as they can provide more genetic evidence and thus indirectly provide more support for the method. Biological repeats would directly show robustness of the method and take away all hesitation. Having said this, the use of such differential screening for mutated variant baits is a powerful feature that will find many applications.

2nd Authors' Response to Reviewers 16th Jan 2022

2nd Revision - Editorial Decision 19th Jan 2022

Thank you again for sending us your revised manuscript. We are now satisfied with the modifications made and I am pleased to inform you that your paper has been accepted for publication.

NOTE:

EMBO PRESS

YOU MUST COMPLETE ALL CELLS WITH A PINK BACKGROUND ↓

PLEASE NOTE THAT THIS CHECKLIST WILL BE PUBLISHED ALONGSIDE YOUR PAPER

Corresponding Author Name: Igor Stagljar

Journal Submitted to: Molecular Systems Biology

Manuscript Number: MSB-2021-10629R

Reporting Checklist For Life Sciences Articles (Rev. June 2017)

This checklist is used to ensure good reporting standards and to improve the reproducibility of published results. These guidelines are consistent with the Principles and Guidelines for Reporting Preclinical Research issued by the NIH in 2014. Please follow the journal's authorship guidelines in preparing your manuscript.

A- Figures
1. Data
The data shown in figures should satisfy the following conditions:

- the data were obtained and processed according to the field's best practice and are presented to reflect the results of the experiments in an accurate and unbiased manner.
- figure panels include only data points, measurements or observations that can be compared to each other in a scientifically meaningful way.
- graphs include clearly labeled error bars for independent experiments and sample sizes. Unless justified, error bars should not be shown for technical replicates.
- if n< 5, the individual data points from each experiment should be plotted and any statistical test employed should be justified
- Source Data should be included to report the data underlying graphs. Please follow the guidelines set out in the author ship guidelines on Data Presentation.

2. Captions
Each figure caption should contain the following information, for each panel where they are relevant:

- a specification of the experimental system investigated (eg cell line, species name).
- the assay(s) and method(s) used to carry out the reported observations and measurements
- an explicit mention of the biological and chemical entity(ies) that are being measured.
- an explicit mention of the biological and chemical entity(ies) that are altered/varied/perturbed in a controlled manner.

- the exact sample size (n) for each experimental group/condition, given as a number, not a range;
- a description of the sample collection allowing the reader to understand whether the samples represent technical or biological replicates (including how many animals, litters, cultures, etc.).
- a statement of how many times the experiment shown was independently replicated in the laboratory.
- definitions of statistical methods and measures:
  - common tests, such as t-test (please specify whether paired vs. unpaired), simple $\chi 2$ tests, Wilcoxon and Mann-Whitney tests, can be unambiguously identified by name only, but more complex techniques should be described in the methods section;
  - are tests one-sided or two-sided?
  - are there adjustments for multiple comparisons?
  - exact statistical test results, e.g., P values = x but not P values < x;
  - definition of 'center values' as median or average;
  - definition of error bars as s.d. or s.e.m.

Any descriptions too long for the figure legend should be included in the methods section and/or with the source data.

In the pink boxes below, please ensure that the answers to the following questions are reported in the manuscript itself. Every question should be answered. If the question is not relevant to your research, please write NA (non applicable). We encourage you to include a specific subsection in the methods section for statistics, reagents, animal models and human subjects.

USEFUL LINKS FOR COMPLETING THIS FORM

http://www.antibodypedia.com
http://1degreebio.org
http://www.equator-network.org/reporting-guidelines/improving-bioscience-research-repor
http://grants.nih.gov/grants/olaw/olaw.htm
http://www.mrc.ac.uk/Ourresearch/Ethicsresearchguidance/Useofanimals/index.htm
http://ClinicalTrials.gov
http://www.consort-statement.org
http://www.consort-statement.org/checklists/view/32-consort/66-title
http://www.equator-network.org/reporting-guidelines/reporting-recommendations-for-tumo
http://datadryad.org
http://figshare.com
http://www.ncbi.nlm.nih.gov/gap
http://www.ebi.ac.uk/ega
http://biomodels.net/
http://biomodels.net/miriam/
http://jjj.biochem.sun.ac.za
https://osp.od.nih.gov/biosafety-biosecurity-and-emerging-biotechnology/
http://www.selectagents.gov/

B- Statistics and general methods

Please fill out these boxes ↓ (Do not worry if you cannot see all your text once you press return)

1.a. How was the sample size chosen to ensure adequate power to detect a pre-specified effect size?	Sample sizes were selected based on common practices and sample availability.
1.b. For animal studies, include a statement about sample size estimate even if no statistical methods were used.	N/A
2. Describe inclusion/exclusion criteria if samples or animals were excluded from the analysis. Were the criteria pre-established?	N/A
3. Were any steps taken to minimize the effects of subjective bias when allocating animals/samples to treatment (e.g. randomization procedure)? If yes, please describe.	N/A
For animal studies, include a statement about randomization even if no randomization was used.	N/A
4.a. Were any steps taken to minimize the effects of subjective bias during group allocation or/and when assessing results (e.g. blinding of the investigator)? If yes please describe.	N/A
4.b. For animal studies, include a statement about blinding even if no blinding was done	N/A
5. For every figure, are statistical tests justified as appropriate?	Statistical tests are justified based on common practices.
Do the data meet the assumptions of the tests (e.g., normal distribution)? Describe any methods used to assess it.	N/A
Is there an estimate of variation within each group of data?	Yes, the variations are presented as appropriate.

Is the variance similar between the groups that are being statistically compared?	Yes

C- Reagents

6. To show that antibodies were profiled for use in the system under study (assay and species), provide a citation, catalog number and/or clone number, supplementary information or reference to an antibody validation profile. e.g., Antibodypedia (see link list at top right), 1DegreeBio (see link list at top right).	The information pertaining to all antibodies used in this manuscript is presented in the 'Reagents and Tools' table.
7. Identify the source of cell lines and report if they were recently authenticated (e.g., by STR profiling) and tested for mycoplasma contamination.	The information pertaining to all cell lines used in this manuscript is presented in the 'Reagents and Tools' table. Cell lines were tested to verify that they were not contaminated with mycoplasma.

* for all hyperlinks, please see the table at the top right of the document

D- Animal Models

8. Report species, strain, gender, age of animals and genetic modification status where applicable. Please detail housing and husbandry conditions and the source of animals.	N/A
9. For experiments involving live vertebrates, include a statement of compliance with ethical regulations and identify the committee(s) approving the experiments.	N/A
10. We recommend consulting the ARRIVE guidelines (see link list at top right) (PLoS Biol. 8(6), e1000412, 2010) to ensure that other relevant aspects of animal studies are adequately reported. See author guidelines, under 'Reporting Guidelines'. See also: NIH (see link list at top right) and MRC (see link list at top right) recommendations. Please confirm compliance.	N/A

E- Human Subjects

11. Identify the committee(s) approving the study protocol.	The organoid study was approved by the research ethics review board (REB) of the Hospital for Sick Children, Toronto, Canada (REB # 1000058992).
12. Include a statement confirming that informed consent was obtained from all subjects and that the experiments conformed to the principles set out in the WMA Declaration of Helsinki and the Department of Health and Human Services Belmont Report.	Informed consent was obtained prior to rectal biopsies using standard endoscopic biopsy forceps.
13. For publication of patient photos, include a statement confirming that consent to publish was obtained.	N/A
14. Report any restrictions on the availability (and/or on the use) of human data or samples.	No restriction on the use of available data.
15. Report the clinical trial registration number (at ClinicalTrials.gov or equivalent), where applicable.	N/A
16. For phase II and III randomized controlled trials, please refer to the CONSORT flow diagram (see link list at top right) and submit the CONSORT checklist (see link list at top right) with your submission. See author guidelines, under 'Reporting Guidelines'. Please confirm you have submitted this list.	N/A
17. For tumor marker prognostic studies, we recommend that you follow the REMARK reporting guidelines (see link list at top right). See author guidelines, under 'Reporting Guidelines'. Please confirm you have followed these guidelines.	N/A

F- Data Accessibility

18: Provide a "Data Availability" section at the end of the Materials & Methods, listing the accession codes for data generated in this study and deposited in a public database (e.g. RNA-Seq data: Gene Expression Omnibus GSE39462, Proteomics data: PRIDE PXD000208 etc.) Please refer to our author guidelines for 'Data Deposition'.<br><br>Data deposition in a public repository is mandatory for:<br>a. Protein, DNA and RNA sequences<br>b. Macromolecular structures<br>c. Crystallographic data for small molecules<br>d. Functional genomics data<br>e. Proteomics and molecular interactions	We have provided a data availability section in the manuscript.
19. Deposition is strongly recommended for any datasets that are central and integral to the study; please consider the journal's data policy. If no structured public repository exists for a given data type, we encourage the provision of datasets in the manuscript as a Supplementary Document (see author guidelines under 'Expanded View' or in unstructured repositories such as Dryad (see link list at top right) or Figshare (see link list at top right).	The interactome data is being deposited in the IID database as described in the manuscript.
20. Access to human clinical and genomic datasets should be provided with as few restrictions as possible while respecting ethical obligations to the patients and relevant medical and legal issues. If practically possible and compatible with the individual consent agreement used in the study, such data should be deposited in one of the major public access-controlled repositories such as dbGAP (see link list at top right) or EGA (see link list at top right).	N/A
21. Computational models that are central and integral to a study should be shared without restrictions and provided in a machine-readable form. The relevant accession numbers or links should be provided. When possible, standardized format (SBML, CellML) should be used instead of scripts (e.g. MATLAB). Authors are strongly encouraged to follow the MIRIAM guidelines (see link list at top right) and deposit their model in a public database such as Biomodels (see link list at top right) or JWS Online (see link list at top right). If computer source code is provided with the paper, it should be deposited in a public repository or included in supplementary information.	N/A

G- Dual use research of concern

22. Could your study fall under dual use research restrictions? Please check biosecurity documents (see link list at top right) and list of select agents and toxins (APHIS/CDC) (see link list at top right). According to our biosecurity guidelines, provide a statement only if it could.	N/A
